# A translational roadmap for transcranial magnetic and direct current stimulation in stroke rehabilitation: Consensus-based core recommendations from the third stroke recovery and rehabilitation roundtable

**DOI:** 10.1177/15459683231209136

**Published:** 2023-10-14

**Authors:** Jodi D Edwards, Adan Ulises Dominguez-Vargas, Charlotte Rosso, Meret Branscheidt, Lisa Sheehy, Fanny Quandt, Simon A Zamora, Melanie K Fleming, Valentina Azzollini, Ronan A Mooney, Charlotte J Stagg, Chiristian Gerloff, Simone Rossi, Leonardo G Cohen, Pablo Celnik, Michael A Nitsche, Cathrin M Buetefisch, Numa Dancause

**Affiliations:** 1 University of Ottawa Heart Institute, Ottawa, ON, Canada; 2 School of Epidemiology and Public Health, University of Ottawa, Ottawa, ON, Canada; 3 Université de Montréal, Montréal, QC, Canada; 4 Institut du Cerveau et de la Moelle épinière, Paris, France; 5 Cereneo Center for Neurology and Rehabilitation, Vitznau, Switzerland; 6 Bruyére Research Institute, Ottawa, ON, Canada; 7 University Medical Center Hamburg-Eppendorf, Hamburg, Germany; 8 University of Oxford, Oxford, UK; 9 National Institutes of Health, Bethesda, MD, USA; 10 Johns Hopkins University, Baltimore, MD, USA; 11 University of Siena, Siena, Italy; 12 Leibniz Research Center for Working Environment and Human Factors, Dortmund, Germany; 13 Emory University, Atlanta, GA, USA

**Keywords:** Stroke, rehabilitation, non-invasive brain stimulation, transcranial magnetic stimulation, transcranial direct current stimulation, stroke recovery and rehabilitation roundtable

## Abstract

**Background and Aims:**

The purpose of this Third Stroke Recovery and Rehabilitation Roundtable (SRRR3) was to develop consensus recommendations to address outstanding barriers for the translation of preclinical and clinical research using the non-invasive brain stimulation (NIBS) techniques Transcranial Magnetic Stimulation (TMS) and Transcranial Direct Current Stimulation (tDCS) and provide a roadmap for the integration of these techniques into clinical practice.

**Methods:**

International NIBS and stroke recovery experts (N = 18) contributed to the consensus process. Using a nominal group technique, recommendations were reached via a five-stage process, involving a thematic survey, two priority ranking surveys, a literature review and an in-person meeting.

**Results and Conclusions:**

Results of our consensus process yielded five key evidence-based and feasibility barriers for the translation of preclinical and clinical NIBS research, which were formulated into five core consensus recommendations. Recommendations highlight an urgent need for (1) increased understanding of NIBS mechanisms, (2) improved methodological rigor in both preclinical and clinical NIBS studies, (3) standardization of outcome measures, (4) increased clinical relevance in preclinical animal models, and (5) greater optimization and individualization of NIBS protocols. To facilitate the implementation of these recommendations, the expert panel developed a new SRRR3 Unified NIBS Research Checklist. These recommendations represent a translational pathway for the use of NIBS in stroke rehabilitation research and practice.

## Graphical abstract

**Graphical Abstract:**
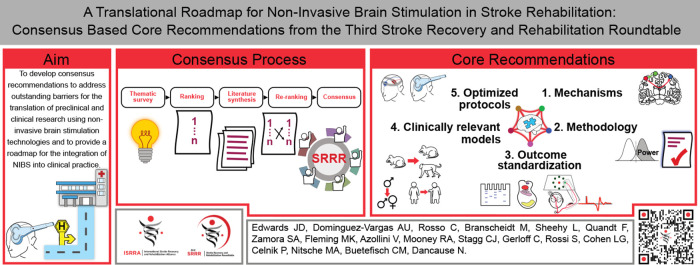
This is a visual representation of the abstract.

## Introduction

Numerous studies have explored non-invasive brain stimulation (NIBS) technologies as a method of modulating human brain activity to gain a deeper understanding of neural circuitry and function in healthy individuals.^1^ They have led to the development of new therapeutic approaches to promote recovery for various neurological conditions, including stroke.^2^ In stroke survivors, transcranial direct current stimulation (tDCS) and repetitive transcranial magnetic stimulation (rTMS) are the two most common NIBS methods, with established safety profiles.^3^ tDCS delivers weak (about 0.5–2.0 mA) currents to the cortex via two polarizing (anodal, cathodal) electrodes to modulate cortical excitability,^4^ while rTMS delivers repetitive magnetic pulses at varying rates, intensities, and frequencies to induce changes in the stimulated neurons and remotely, in interconnected brain regions.^5^ The present recommendations focus on evidence from both preclinical (animal stroke model and healthy adults) and clinical (stroke populations) research to support the therapeutic use of tDCS and rTMS.

Over 30 years of research using NIBS in animal models and healthy adults has established that both tDCS and rTMS induce controllable synaptic changes akin to long-term potentiation and depression ^6^ and can generate lasting alterations in cortical excitability, promoting brain plasticity.^7^ In stroke populations, hundreds of interventional studies and randomized controlled trials have examined the efficacy of NIBS interventions to optimize rehabilitation treatment outcome for multiple post-stroke deficits, including motor impairment, aphasia, dysphagia and neglect at various phases of recovery.^8^–^10^ While these findings have influenced clinical guidelines (e.g. Level B evidence, tDCS for motor rehabilitation;^11^ Level A evidence, low-frequency rTMS (LF-rTMS) for hand function; Level C evidence, tDCS for post-stroke aphasia),^12^ they have been insufficient to change rehabilitation practice. Although NIBS is an approved stand-alone therapeutic intervention for major depression and pain in several jurisdictions,^13^ stroke rehabilitation guidelines are just beginning to acknowledge the potential of NIBS for post-stroke recovery.^14,15^ There are thus several major barriers to advancement of TMS and tDCS as therapeutic tools that urgently need to be addressed to accelerate translation. Foremost, we need to identify critical evidence-based and feasibility barriers that have limited the development of optimized protocols and the ability to conduct large, definitive phase 3 trials. The purpose of this Third Stroke Recovery and Rehabilitation Roundtable (SRRR3) was to develop consensus recommendations to identify and address outstanding translational barriers and provide a roadmap for the use of TMS and tDCS for stroke rehabilitation.

## Methodology

**Figure 1.**
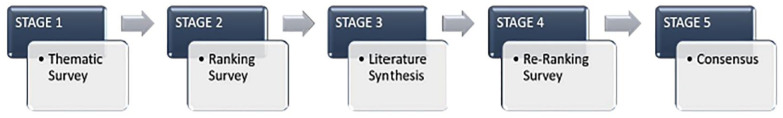
Five-stage consensus building process.

A total of 18 basic and clinical scientists with expertise in NIBS and stroke recovery contributed to the consensus process from January 2022 to June 2023. In accordance with SRRR guidelines, panelist selection included approximately equal sex representation, with individuals across career stages and broad geographical locations. Each expert was also encouraged to include one trainee with experience in NIBS to support consensus activities. A full description of the methodology is provided in Supplemental Appendix 1a. Consensus recommendations were reached following a five-stage nominal group process^16^ (Figure 1). In a thematic survey, experts first identified up to 10 evidence gaps, barriers, and needs for the translation of preclinical and clinical NIBS research (Supplemental Appendix 1b). Evidence gaps and barriers were then combined and ranked by *priority* and *feasibility* to address the needs. To ensure consensus recommendations reflected current knowledge and prevent unconscious bias, we conducted a review of recent (last 10 years) preclinical and clinical NIBS research (Supplemental Appendix 1c–g; Figure S1). After this review, barriers were re-ranked and the five highest were brought to the consensus discussion (Supplemental Table S1).

## Results summary and recommendations

We identified 12 evidence-based and seven feasibility barriers for the translation of preclinical and clinical findings (Supplemental Table S1). As several barriers were common across fields, they were grouped into five major knowledge gaps. Sections below describe the evidence-based and feasibility barriers for each knowledge gap, and the five consensus recommendations formulated to address them (Supplemental Table S2).

### Knowledge gap 1: NIBS mechanisms

The greatest knowledge gap limiting the translation of preclinical NIBS findings was a lack of mechanistic understanding of NIBS, and this remained the highest priority after re-ranking. This includes potential effects on mechanisms such as gene expression, neurotransmission and cellular excitability that can be affected locally at the stimulation site, but also distally, across areas of the targeted network (e.g. sensorimotor or language). The literature review revealed two main evidence-based translational barriers for this gap, including a lack of (1) studies systematically comparing stimulation parameters within and across stimulation modalities and, as such, a lack of evidence for the mechanisms of response to NIBS interventions and (2) studies providing justification for the stimulation target within a neurobiological framework and target engagement.

Previous recommendations: Prior SRRR2 trial development recommendations highlighted the need for systematic preclinical and clinical dose studies stroke recovery interventions;^17^ however, no prior recommendations have addressed systematic testing or target engagement specifically for NIBS and this gap has previously been noted in tDCS guidelines.^11^

Evidence-based translational barriers: *(a) Systematic NIBS parameter testing*: For both tDCS and rTMS, the combination of parameter settings is large. To date, systematic studies of stimulation parameters have been mostly limited to healthy young adults, targeting M1, and using only physiological outcome measures (e.g. motor outputs).^18^ In the post-stroke population, there are numerous considerations that limit the extrapolation of findings from the young healthy literature directly, including aging, post-lesion plasticity, vascular burden, lesion characteristics (size/location), post-stroke inflammatory processes or medication. In our literature synthesis, the most studied parameters were electrode polarity for tDCS and pulse frequency for rTMS. However, less than 10% of animal studies and clinical studies/trials compared stimulation parameters or dosing within or across modalities. The investigation of the effects of parameters on the molecular and cellular responses in the brain (e.g. neuronal excitability, inflammation, etc.) and to behavioral outcomes was scarce. This lack of systematic testing limits our understanding of the effect of specific parameters on the targeted brain area, precluding comparisons across studies using different parameters and the optimization of stimulation protocols.

*(b) Target engagement*: The second major translational barrier is related to the choice/location of the stimulation target. Most studies (>90%) targeted a single cortical area, without confirmation of its involvement in impairment or recovery. For example, most post-stroke motor recovery trials targeted either the ipsilesional or contralesional M1, regardless of the level of impairment, lesion characteristics or confirmation of atypical activity in this area during behavior. However, these factors can affect the pattern of brain activation during behavior, and accordingly the NIBS target and after-effects. To enable the design of effective RCTs in stroke populations, preclinical studies (both in animals and healthy adults) must justify the selection of the cortical target using evidence-based biological frameworks that allow for primary hypothesis testing and include evaluation of causal links between stimulation, target engagement and behavioral effects. For future clinical trials, inclusion of lesion characteristics (e.g. volume, structures and pathways involved) is critical. Ideally, trials should include physiological/imaging measures (e.g. motor evoked potentials (MEPs), functional magnetic resonance imaging (fMRI) or electroencephalography (EEG)) to quantify target engagement beyond behavioral effects. As these data accumulate, for example through shared repositories allowing retrospective studies, we will be able to refine our mechanistic hypotheses about the effects of the lesion on brain reorganization and individualize the choice of target brain area and stimulation parameters. Although practically, this must be balanced with effective recruitment and integration of stimulation into clinical workflow. Multi-center trials may need to focus either on mechanisms using physiological/imaging methods in more moderate sample sizes or clinical outcomes and treatment efficacy in larger phase-III trials, each of which has a different purpose and design considerations.

Feasibility translational barriers: Under the current peer-reviewed funding system, studies systematically testing NIBS parameters and providing incremental knowledge about mechanisms and dose/effect relationships have difficulty achieving a high priority. Thus, consistent with prior general stroke recovery recommendations,^17^ we also strongly recommend that funding agencies offer increased and dedicated support for these studies in NIBS to provide key evidence for the design of effective large RCTs.

**Table:** 

Recommendation 1
Preclinical and clinical studies/trials should: (a) systematically compare stimulation parameters within and across modalities and quantify the effects of these parameters on the brain and behavior, and; (b) use an evidence-based biological framework for target selection and confirm the intervention effect at the level of the target.

### Knowledge gap 2: methodological rigor

The second highly ranked gap concerned the methodological rigor of existing studies. The main translational barriers regarding study methodology were: (1) limitations in design, (2) a lack of adequate sample sizes and statistical power to test the therapeutic benefits of NIBS, and (3) a lack of transparency in reporting. Notably, while initially receiving a lower priority ranking, this item increased after the literature synthesis, with major concerns regarding clinical trial methodology emerging.

Previous recommendations: Prior SRRR recommendations emphasized the importance of methodological rigor and adequate reporting in stroke recovery research^19^ including the use of TIDierR^20^ for interventions and CONSORT^21^ flow diagrams for reporting, and ARRIVE guidelines^22^ for preclinical studies. However, there are no recommendations specific to the use of NIBS in preclinical research. There is only one prior set of recommendations for methodology and reporting of NIBS for clinical research,^23^ with an accompanying checklist for single or paired pulse TMS that was also adapted to tDCS.^24^ However, there is no comprehensive, unified cross-modality checklist for both preclinical and clinical NIBS research.

Evidence-based translational barriers: *(a) NIBS methodology*: For preclinical studies, almost all were single center and very few involved pre-registration to ensure the internal and external validity of findings. In addition, across studies, there was a lack of inclusion of sham stimulation (~1/3 of studies) and poor reporting of critical stimulation parameters. For clinical trials, most trials (>85%) were single center, and while almost half of tDCS trials were pre-registered, only ~20% of TMS trials reported pre-registration. Many trials reported eligibility criteria, but the inclusion of important clinical selection criteria (i.e. physiology, lesion location/size, recovery phase) was highly variable and almost none reported how patients were excluded based on neurophysiological findings. Although most trials included some form of blinding, the majority were single blinded (65%) and very few reported testing the efficacy of blinding. The majority of tDCS trials involved the use of sham stimulation. However, only ~75% of TMS trials included a sham. Across modalities there was a complete lack of standardization of sham procedures, with tDCS sham durations ranging from 5 s to 2 min and TMS sham involving a broad range of methods including varying coil types, orientation, and distance from the target location. Finally, while many trials reported pairing stimulation with some form of therapy, almost 1/3 did not and the details of the rehabilitation therapy were poorly reported. These limitations represent a major translational barrier that aside from a small number of well reported trials^25^ resulted in an overall low quality of evidence and substantial heterogeneity in results.^12,14^

*(b) Sample size and statistical power*: Preclinical studies with animal models largely comprised multiple different subgroups, each with small sample sizes. Consequently, very few studies (<5%) were sufficiently powered to test the primary hypothesis, with many reporting non-significant effects or small effects in only one or few subgroups, based on very limited data, and none reporting power analyses. In humans, the majority of RCTs were small, with over 70% reporting sample sizes of <N = 50 and only a few trials of N > 100 across all modalities and deficit types. Critically, less than 1/3 of trials reported power analyses to test the efficacy of primary endpoints. Reporting for both the statistical methodology used and observed effect sizes was very sparse.

*(c) Transparency of reporting*: Across both preclinical and clinical studies and all NIBS modalities, reporting of patient characteristics (lesion size/location, severity), stimulation and sham parameters, paired rehabilitation, and statistical methods and power was very poor, limiting our ability to interpret and synthesize evidence for the efficacy of NIBS. The consistent use of reporting checklists specific to NIBS would facilitate replication of NIBS protocols and meta-analyses and improve translation of NIBS study results from preclinical to clinical domains. To address this gap, we developed a Unified Checklist for NIBS Research (Table 1) for use in either preclinical or clinical studies involving tDCS and rTMS and including a comprehensive reporting of stimulation parameters and targets. We strongly recommend the use of this checklist in conjunction with other recommended design and reporting checklists (i.e. ARRIVE, CONSORT, and TIDieR) in all future NIBS preclinical and clinical stroke studies.

Feasibility barriers: Many studies did not adhere to current reporting guidelines, resulting in major challenges with the interpretation and translation of findings. *Stroke* (https://www.ahajournals.org/journal/str) has established requirements to improve transparent reporting practices. We recommend that more journals require strict adherence to recommended design and reporting checklists before acceptance, for both preclinical studies and clinical trials. In addition, multiple feasibility challenges were identified that limit the integration of NIBS techniques into clinical workflow, including the cost of equipment, need and availability of trained operators, and protocol length and cheaper, automated systems may be required for widespread uptake of these technologies.

**Table:** 

Recommendation 2
Preclinical and clinical studies/trials should: (a) include pre-registration and use of appropriate patient eligibility criteria, blinding and sham stimulation protocols, and appropriate paired therapies; (b) conduct and report prospective power analysis to determine samples sizes appropriate to test primary hypotheses, and; (c) use the SRRR3 Unified Checklist for NIBS Research and adhere to current recommended design and reporting guidelines.

### Knowledge gap 3: outcome standardization

The next highly ranked gap related to the standardization of outcomes measures. The main translational barriers to address this gap were a lack of: (1) preclinical studies including behavioral in addition to physiological and cellular-molecular outcome measures to provide a complete assessment across domains; (2) standardized preclinical outcome assessment tools for domains other than sensorimotor; and (3) studies/clinical trials that reported minimally important clinical differences for standardized outcomes that were aligned with International Classification of Functioning, Disability, and Health (ICF) categories or study hypotheses.

Previous recommendations: Previous SRRR recommendations have focused on the standardized measurement of core tissue and behavioral outcomes for preclinical stroke recovery research^26^ and both sensorimotor recovery^27^ and upper limb movement quality^28^ in stroke recovery clinical trials. There are currently no recommendations specific to outcomes for NIBS research.

Evidence-based translational barriers: *(a) Multi-domain and standardized preclinical outcomes.* NIBS studies in animal models were largely biased toward post-mortem evaluations of the cellular and molecular impacts of stimulation (approximately 60%). The most commonly reported outcome across animal studies was the modified neurological severity score, a standardized tool that provides a global behavioral assessment. Other common but non-standardized measures were changes in neurogenesis, cell migration, and neuroprotection assessed by immunohistochemistry and western blot techniques. Very few studies provided detailed behavioral or physiological assessments. There is thus a lack of comparable outcome measures between animal and human studies, creating a major limitation for the translation of preclinical findings. Future studies in animal models should include behavioral assessments, such as kinematics and other biomarkers (e.g. brain imaging) that are paralleled in human studies and better characterize the physiological properties and NIBS-induced alterations of the stimulation target.

*(b) Minimally important differences*. Our review of human NIBS RCTs for motor recovery generally showed good use of standardized outcome measures. For studies targeting the therapeutic use of TMS or tDCS for recovery of the upper extremity, the Fugl-Meyer (FMA-UE) and Action Research Arm Test (ARAT) were the most used measures (>80%). Both have acceptable intra- and inter-rater variability and have been previously recommended.^29^ They also have the important advantage of involving a standardized minimal clinically important difference. For the lower extremity, FMA-LE, another standardized measure, was most frequently reported (~50%). Few studies included activity outcomes for walking (e.g. 10-m walk test gait speed) and there was a lack of assessment of movement quality with kinetic and kinematic measures, despite its use to distinguish true neural restitution from behavioral compensation during recovery.^27^ Finally, few studies reported selecting and aligning outcomes with ICF categories^30^ and several studies did not use outcome measures that directly aligned with the study hypothesis (e.g. hypothesis specific to skilled hand function but outcome not a direct measure).^31^

Unlike the motor domain, there was a striking lack of standardization of outcomes in studies in other domains, including aphasia, dysphagia, and cognition. Most trials included non-standardized measures with unclear psychometric properties (e.g. author-created questionnaires or tests), resulting in concerns about outcome utility and reduced generalizability. Finally, while studies included multiple behavioral outcomes (e.g. motor function, activity and kinematics), few included *both* physiological and behavioral measures, which is crucial to link stimulation protocols to physiological mechanisms and behavior.

Feasibility barriers: No feasibility barriers were identified with respect to outcomes.

**Table:** 

Recommendation 3
(a) Preclinical studies should: (i) conduct complete outcome assessments across cellular-molecular, physiological and behavioral domains, and; (ii) include standardized behavioral outcomes common to human studies (b) Clinical studies/trials should: (i) use standardized assessments with established psychometric properties, and; (ii) report the minimal clinically important difference for outcomes that align with ICF categories and/or study hypotheses.

**Table 1.** SRRR3 unified checklist for NIBS research.

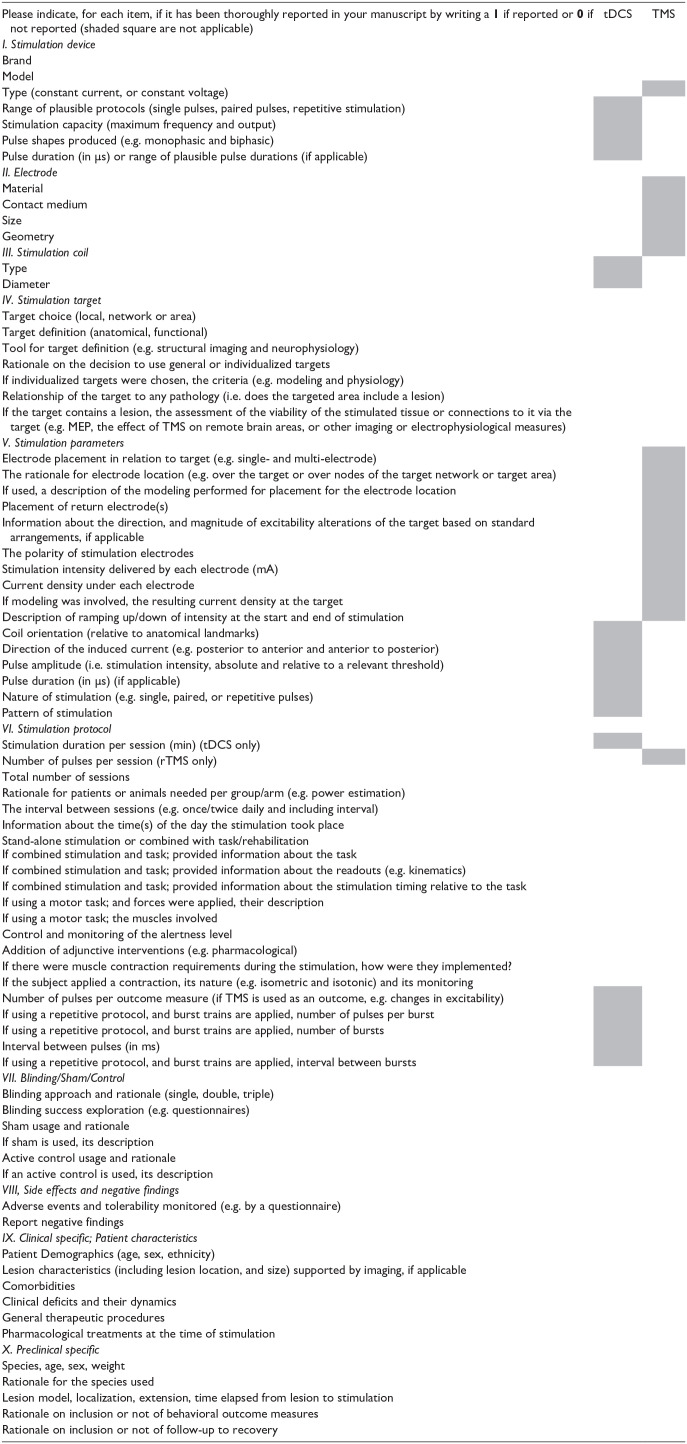

TMS: transcranial magnetic stimulation; MEP: motor evoked potential.

### Knowledge gap 4: clinically relevant preclinical animal models

The next gap emerging from the survey was the need for preclinical models with increased clinical relevance. The main translational barriers were a lack of (1) measures to test interactions between the effects of NIBS on the brain and behavior; (2) studies that directly align NIBS stimulation parameters with those commonly used in humans and that closely replicate the effects that commonly used protocols for humans have on the brain (e.g. considering brain size and/or cortical architecture); (3) studies that test NIBS effects according to lesion characteristics (i.e. lesion location and size); and (4) animal models with comorbidities (i.e. aged animals or animal strains with spontaneous diseases, such as vasculopathies and diabetes).

Previous recommendations: The first set of SRRR recommendations stressed the urgency for the development of robust and diverse preclinical models that more accurately reflect patient profiles.^32^ There are currently no recommendations specific to preclinical models for NIBS research.

Evidence-based translational barriers: *(a) Brain–behavior Interactions.* In our literature review, no study involving preclinical stroke models specifically tested interactions between the effects of NIBS on the brain and behavioral outcomes. This is a missed opportunity, considering that relevant post-stroke variables (i.e. lesion location, age, and comorbidities) can be more easily controlled in these models and facilitate the evaluation of interactions. This evidence is critical to delineate potential joint and cumulative effects of NIBS on behavior across different lesion profiles and clinical phenotypes.

*(b) Aligning stimulation parameters and replicating effects of NIBS in humans.* Other key limitations of NIBS preclinical animal studies included the divergence from stimulation parameters typically used in human studies and the high degree of variability in stimulation equipment. For example, many tDCS studies have reported neuroprotective or anti-inflammatory effects using stimulation parameters not currently used in practice in human studies.^33^ Several rTMS studies in rodents use large commercial coils designed for humans^34^ or small custom-made coils with limited information about the relative volume of the stimulation in relation to brain size.^35^ Yet, there is evidence that small coils still result in relatively non-focal stimulation^36^ and thus the modulation of very large areas in proportion to the size of small rodent brains.

To facilitate direct comparisons with humans some neuromodulation studies with other modalities (e.g. deep brain stimulation (DBS)) are increasingly using pig models. The pig brain is gyrencephalic, has a similar white to gray matter ratio and is large enough to accommodate human DBS electrodes, increasing the translational value of findings, such as the characterization of the effects of different stimulation protocols on local excitability or circuit function.^37^ For NIBS, our literature review highlighted that most animal NIBS studies used rodent models (75%), with only a small proportion in other mammals with larger brains, including non-human primates (NHP). Alternative animal models with cortical architecture more similar to humans (e.g. pigs, sheep, or NHP) should be considered for experimental questions regarding NIBS efficacy or mechanisms of action. To facilitate translation to clinical populations, care should be taken in all models to align and approximate effects that can be safely delivered in human stroke population.

*(c) Lesion characteristics and models with comorbidities.* The majority of studies investigating the effects of NIBS on preclinical models of stroke focused on otherwise healthy young adult male rodents (~70%) after middle cerebral artery occlusion. There is thus a lack of studies testing the effects of NIBS across various lesions profiles, including in aged animals, animals with and without comorbidities, and across sexes.^26^

Feasibility barriers: Although there are a few readily available TMS coil technologies suitable for animal models adjusted to the relative size of the brain, there is still a lack of accessibility of specifications to inform researchers on how these compare to human coils in regard to the characteristics of the electric fields, ideally using both modeling and physiological assessments. This poses a major translational barrier for the interpretation of preclinical NIBS research. There is an urgent need for standardized equipment and stimulation protocols in animals that are better aligned with those used in human studies. We recommend that neurostimulation equipment manufacturers prioritize the development of coil models and the sharing of their coil validation processes using models or simulations with appropriate brain morphologies and topographies.^38^ As these new, well-characterized tools become more accessible, they should be prioritized in animal studies designed to study the effects of TMS in the brain.

**Table:** 

Recommendation 4
Preclinical studies should use stroke animal models that: (a) include head-equipment size relationship, aged animals, comorbidities, behavioral assessments, and the clinical trajectory of recovery in humans.

### Knowledge gap 5: optimized and individualized NIBS protocols

The final gap identified was a need to accelerate the identification of NIBS stimulation parameters/protocols with high potential for efficacy, NIBS treatment response phenotypes, and biomarkers for the development of individualized NIBS protocols. The main translational barriers for this gap were a lack of (1) animal studies testing the effect of NIBS on functional recovery across domains; (2) clinical studies with sufficiently broad inclusion/exclusion criteria and sufficient sample sizes to enable subgroup analyses for the identification of NIBS treatment response phenotypes; and (3) evidence for biomarkers predictive of NIBS treatment response.

Previous recommendations: The first SRRR made recommendations to advance preclinical to clinical pipelines for stroke recovery research^26^ and identify biomarkers of stroke recovery.^39^ However, to date, there are no recommendations for protocol optimization or biomarker identification specific to NIBS.

Evidence-based translational barriers: *(a) Preclinical response domains*. Most preclinical studies (approximately 60%) focused on the use of tDCS or rTMS to promote neuroprotection and plasticity after stroke in relation to motor recovery,^40^ with few studies testing functional recovery across multiple domains. These studies have provided crucial information about glial differentiation, neuronal gene regulation, and other molecular changes induced by NIBS. However, more preclinical evidence specifically linking the effects of NIBS to behavior across recovery domains (e.g. motor, sensory, cognition, etc.) is required to improve clinical translation.

*(b) Identification of NIBS response phenotypes*: As previously highlighted, most trials included in our literature synthesis were small (over 70% with N < 50), precluding the analysis of treatment benefits for subgroups with varying characteristics. While some trials included broad criteria with both cortical and subcortical patients from 6 months to many years post-stroke and even sometimes a mixture of ischemic and hemorrhagic etiologies, the majority were too small to enable meaningful subgroup analyses. Conversely, other trials reported narrow inclusion criteria, enrolling patients only within certain age ranges, time window post-stroke, or with specific deficit severity, resulting in low recruitment potential and again low sample sizes. This latter approach can reduce variability due to heterogeneous patient characteristics, but also limits the disaggregation and generalizability of findings by subgroups. Importantly, meta-analyses based on numerous small trials cannot mitigate biases inherent in the original trials. Thus, larger trials across a broad range of inclusion criteria sufficiently powered for subgroup analyses are needed for the identification of response phenotypes.

*(c) Biomarkers of NIBS treatment response*: There is currently a lack of evidence for biomarkers predictive of NIBS response. In the reviewed studies, patient enrollment was rarely based on a specific biomarker. However, some trials included patients based on neurophysiological characteristics, such as the presence or absence of MEPs. Demographic factors, such as age and sex, do not show clear utility in predicting response to NIBS protocols^41^ and while time post-stroke may influence response magnitude,^42^ most clinical studies identified in our literature synthesis involved either chronic or mixed and not acute/subacute patient populations. White matter connectivity or measures of neuronal oscillations have previously been identified as biomarkers with potential for stroke recovery^39,43^ and prior tDCS work suggests that EEG connectivity may explain variability in corticospinal excitability changes.^44^ Furthermore, the presence of Val66Met brain-derived neurotrophic factor (BDNF) polymorphisms influence motor cortical excitability in stroke patients,^45^ as well as responses to TMS outside motor cortex.^46^

A major limitation of existing biomarker studies is the variable use of statistical methods appropriate to establish predictive utility and the lack of calibration and internal validation of prediction models. For example, prior predictive algorithms for NIBS response based on a combination of motor strength, corticospinal tract integrity and imaging parameters have been proposed.^47^ However, subsequent simulation studies have shown that scale properties dramatically impact model fit^48^ and these algorithms did not involve methods for model calibration and validation.^49^ To facilitate the development of individualized NIBS protocols, future studies assessing the predictive utility of potential biomarkers should employ best practices for model development and calibration and follow TRIPOD guidelines for reporting.^50^

Feasibility barriers: A major challenge with reproducibility is a lack of data repositories to consolidate existing preclinical and clinical evidence that enable meta-analyses. Large-scale repositories, such as VISTA-Rehab^51^ are good initial forays into large-scale repositories. However, they are not focused on NIBS interventions. Increased international coordination for publicly available harmonized NIBS repositories is required.

The identification of response phenotypes and predictive biomarkers is limited by the design of traditional clinical trials, of which few have sufficient sample sizes to support analyses of response subtypes. Increased funding for and use of innovative adaptive trial designs^52^ with flexibility to integrate new knowledge of protocols with high potential for efficacy and individualized NIBS prescriptions is a promising new translational avenue for NIBS. These designs are particularly advantageous when considering the number of experimental surrogates needed to account for the stratification of stroke patient subpopulations, and the need to differentiate putative contributions of multiple response predictors in NIBS stroke models, ranging from electrophysiology, to imaging, behavior, and omics. The adoption of adaptive designs can prove critical, as preclinical studies involving recovery might otherwise become unfeasible. Long-lasting behavioral and electrophysiological testing necessary to assess functional changes after NIBS could be performed in late phases of mechanistic studies and only with small subsets of animals. This would broaden the comprehension of protocol efficacy without compromising the viability of the study’s execution.

**Table:** 

Recommendation 5
Preclinical and clinical studies/trials should: (a) test multi-domain NIBS response; (b) have sufficient sample sizes to identify response phenotypes; and (c) use appropriate statistical methodology to identify predictive biomarkers.

## Conclusion

Non-invasive brain stimulation technologies, and specifically rTMS and tDCS as the focus of this current consensus exercise, have a long history of experimental and clinical evidence supporting they are safe and can induce rapid and reproducible effects on the brain. Importantly, they show promising potential therapeutic benefits for the improvement of multiple post-stroke deficits. However, several major translational barriers have limited their advancement as a clinical tool for stroke recovery. The consensus recommendations and SRRR3 Unified NIBS Checklist developed by this roundtable are designed to address these outstanding barriers and provide a roadmap for the integration of TMS and tDCS technologies into clinical practice for stroke rehabilitation.

## Supplemental Material

sj-docx-1-nnr-10.1177_15459683231209136 – Supplemental material for A translational roadmap for transcranial magnetic and direct current stimulation in stroke rehabilitation: Consensus-based core recommendations from the third stroke recovery and rehabilitation roundtable Click here for additional data file. Supplemental material, sj-docx-1-nnr-10.1177_15459683231209136 for A translational roadmap for transcranial magnetic and direct current stimulation in stroke rehabilitation: Consensus-based core recommendations from the third stroke recovery and rehabilitation roundtable by Jodi D Edwards, Adan Ulises Dominguez-Vargas, Charlotte Rosso, Meret Branscheidt, Lisa Sheehy, Fanny Quandt, Simon A Zamora, Melanie K Fleming, Valentina Azzollini, Ronan A Mooney, Charlotte J Stagg, Chiristian Gerloff, Simone Rossi, Leonardo G Cohen, Pablo Celnik, Michael A Nitsche, Cathrin M Buetefisch and Numa Dancause in Neurorehabilitation and Neural Repair

sj-docx-2-nnr-10.1177_15459683231209136 – Supplemental material for A translational roadmap for transcranial magnetic and direct current stimulation in stroke rehabilitation: Consensus-based core recommendations from the third stroke recovery and rehabilitation roundtable Click here for additional data file. Supplemental material, sj-docx-2-nnr-10.1177_15459683231209136 for A translational roadmap for transcranial magnetic and direct current stimulation in stroke rehabilitation: Consensus-based core recommendations from the third stroke recovery and rehabilitation roundtable by Jodi D Edwards, Adan Ulises Dominguez-Vargas, Charlotte Rosso, Meret Branscheidt, Lisa Sheehy, Fanny Quandt, Simon A Zamora, Melanie K Fleming, Valentina Azzollini, Ronan A Mooney, Charlotte J Stagg, Chiristian Gerloff, Simone Rossi, Leonardo G Cohen, Pablo Celnik, Michael A Nitsche, Cathrin M Buetefisch and Numa Dancause in Neurorehabilitation and Neural Repair

## References

[1] Siebner HR Funke K Aberra AS , et al. Transcranial magnetic stimulation of the brain: what is stimulated?—a consensus and critical position paper. Clin Neurophysiol 2022; 140: 59–97. 35738037 10.1016/j.clinph.2022.04.022 PMC9753778

[2] Guggisberg AG Koch PJ Hummel FC Buetefisch CM . Brain networks and their relevance for stroke rehabilitation. Clin Neurophysiol 2019; 130: 1098–1124. 31082786 10.1016/j.clinph.2019.04.004 PMC6603430

[3] Rossi S Antal A Bestmann S , et al. Safety and recommendations for TMS use in healthy subjects and patient populations, with updates on training, ethical and regulatory issues: expert guidelines. Clin Neurophysiol 2021; 132: 269–306. 33243615 10.1016/j.clinph.2020.10.003 PMC9094636

[4] Nitsche MA Doemkes S Karaköse T , et al. Shaping the effects of transcranial direct current stimulation of the human motor cortex. J Neurophysiol 2007; 97: 3109–3117. 17251360 10.1152/jn.01312.2006

[5] Hoogendam JM Ramakers GM Di Lazzaro V . Physiology of repetitive transcranial magnetic stimulation of the human brain. Brain Stimul 2010; 3: 95–118. 20633438 10.1016/j.brs.2009.10.005

[6] Huerta PT Volpe BT . Transcranial magnetic stimulation, synaptic plasticity and network oscillations. J Neuroeng Rehabil 2009; 6: 7. 19254380 10.1186/1743-0003-6-7 PMC2653496

[7] Di Lazzaro V Rothwell JC . Corticospinal activity evoked and modulated by non-invasive stimulation of the intact human motor cortex. J Physiol 2014; 592: 4115–4128. 25172954 10.1113/jphysiol.2014.274316 PMC4215763

[8] He Y Li K Chen Q Yin J Bai D . Repetitive transcranial magnetic stimulation on motor recovery for patients with stroke: a PRISMA compliant systematic review and meta-analysis. Am J Phys Med Rehabil 2020; 99: 99–108. 31361620 10.1097/PHM.0000000000001277

[9] Kielar A Patterson D Chou YH . Efficacy of repetitive transcranial magnetic stimulation in treating stroke aphasia: systematic review and meta-analysis. Clin Neurophysiol 2022; 140: 196–227. 35606322 10.1016/j.clinph.2022.04.017

[10] Wang T Dong L Cong X , et al. Comparative efficacy of non-invasive neurostimulation therapies for poststroke dysphagia: a systematic review and meta-analysis. Neurophysiol Clin 2021; 51: 493–506. 34535361 10.1016/j.neucli.2021.02.006

[11] Fregni F El-Hagrassy MM Pacheco-Barrios K , et al. Evidence-based guidelines and secondary meta-analysis for the use of transcranial direct current stimulation in neurological and psychiatric disorders. Int J Neuropsychopharmacol 2021; 24: 256–313. 32710772 10.1093/ijnp/pyaa051 PMC8059493

[12] Lefaucheur J-P Aleman A Baeken C , et al. Evidence-based guidelines on the therapeutic use of repetitive transcranial magnetic stimulation (rTMS): an update (2014–2018). Clin Neurophysiol: Off J Int Feder Clin Neurophysiol 2020; 131: 474–528. 10.1016/j.clinph.2019.11.002 31901449

[13] Antal A Alekseichuk I Bikson M , et al. Low intensity transcranial electric stimulation: safety, ethical, legal regulatory and application guidelines. Clin Neurophysiol 2017; 128: 1774–1809. 28709880 10.1016/j.clinph.2017.06.001 PMC5985830

[14] Teasell R Salbach NM Foley N , et al. Canadian stroke best practice recommendations: rehabilitation, recovery, and community participation following stroke—part one: rehabilitation and recovery following stroke; 6th edition update 2019. Int J Stroke 2020; 15: 763–788. 31983296 10.1177/1747493019897843

[15] Winstein CJ Stein J Arena R , et al. Guidelines for adult stroke rehabilitation and recovery: a guideline for healthcare professionals from the American Heart Association/American Stroke Association. Stroke 2016; 47: e98–e169. 10.1161/STR.0000000000000098 27145936

[16] McMillan SS King M Tully MP . How to use the nominal group and Delphi techniques. Int J Clin Pharm 2016; 38: 655–662. 26846316 10.1007/s11096-016-0257-x PMC4909789

[17] Bernhardt J Hayward KS Dancause N , et al. A stroke recovery trial development framework: consensus-based core recommendations from the Second Stroke Recovery and Rehabilitation Roundtable. Int J Stroke 2019; 14: 792–802. 31658893 10.1177/1747493019879657

[18] Ngomo S Leonard G Moffet H Mercier C . Comparison of transcranial magnetic stimulation measures obtained at rest and under active conditions and their reliability. J Neurosci Methods 2012; 205: 65–71. 22227444 10.1016/j.jneumeth.2011.12.012

[19] Walker MF Hoffmann TC Brady MC , et al. Improving the development, monitoring and reporting of stroke rehabilitation research: consensus-based core recommendations from the Stroke Recovery and Rehabilitation Roundtable. Neurorehabil Neural Repair 2017; 31: 877–884. 29233072 10.1177/1545968317732686

[20] Hoffmann TC Glasziou PP Boutron I , et al. Better reporting of interventions: template for intervention description and replication (TIDieR) checklist and guide. BMJ 2014; 348: g1687. 10.1136/bmj.g1687 24609605

[21] Hopewell S Clarke M Moher D , et al. CONSORT for reporting randomised trials in journal and conference abstracts. Lancet 2008; 371: 281–283. 10.1016/S0140-6736(07)61835-2 18221781

[22] Kilkenny C Browne WJ Cuthill IC Emerson M Altman DG . Improving bioscience research reporting: the ARRIVE guidelines for reporting animal research. Osteoarthr Cartil 2012; 20: 256–260. 10.1016/j.joca.2012.02.010 22424462

[23] Chipchase L Schabrun S Cohen L , et al. A checklist for assessing the methodological quality of studies using transcranial magnetic stimulation to study the motor system: an international consensus study. Clin Neurophysiol 2012; 123: 1698–1704. 22647458 10.1016/j.clinph.2012.05.003 PMC4884647

[24] Buch ER Santarnecchi E Antal A , et al. Effects of tDCS on motor learning and memory formation: a consensus and critical position paper. Clin Neurophysiol 2017; 128: 589–603. 28231477 10.1016/j.clinph.2017.01.004

[25] Fridriksson J Rorden C Elm J Sen S George MS Bonilha L . Transcranial direct current stimulation vs Sham stimulation to treat Aphasia after stroke: a randomized clinical trial. JAMA Neurology 2018; 75: 1470–1476. 30128538 10.1001/jamaneurol.2018.2287 PMC6583191

[26] Corbett D Carmichael ST Murphy TH , et al. Enhancing the alignment of the preclinical and clinical stroke recovery research pipeline: consensus-based core recommendations from the Stroke Recovery and Rehabilitation Roundtable translational working group. Int J Stroke 2017; 12: 462–471. 28697710 10.1177/1747493017711814

[27] Kwakkel G Lannin NA Borschmann K , et al. Standardized measurement of sensorimotor recovery in stroke trials: consensus-based core recommendations from the Stroke Recovery and Rehabilitation Roundtable. Int J Stroke 2017; 12: 451–461. 28697709 10.1177/1747493017711813

[28] Kwakkel G Van Wegen E Burridge JH , et al. Standardized measurement of quality of upper limb movement after stroke: consensus-based core recommendations from the Second Stroke Recovery and Rehabilitation Roundtable. Int J Stroke 2019; 14: 783–791. 31510885 10.1177/1747493019873519

[29] Gladstone DJ Danells CJ Black SE . The Fugl-Meyer assessment of motor recovery after stroke: a critical review of its measurement properties. Neurorehabil Neural Repair 2002; 16: 232–240. 12234086 10.1177/154596802401105171

[30] International classification of functioning, disability and health 2001. https://www.who.int/standards/classifications/international-classification-of-functioning-disability-and-health

[31] Chen G Lin T Wu M , et al. Effects of repetitive transcranial magnetic stimulation on upper-limb and finger function in stroke patients: a systematic review and meta-analysis of randomized controlled trials. Front Neurol 2022; 13: 940467. 35968309 10.3389/fneur.2022.940467 PMC9372362

[32] Bernhardt J Borschmann K Boyd L , et al. Moving rehabilitation research forward: developing consensus statements for rehabilitation and recovery research. Neurorehabil Neural Repair 2017; 31: 694–698. 28803534 10.1177/1545968317724290

[33] Kaviannejad R Karimian SM Riahi E Ashabi G . Using dual polarities of transcranial direct current stimulation in global cerebral ischemia and its following reperfusion period attenuates neuronal injury. Metab Brain Dis 2022; 37: 1503–1516. 35499797 10.1007/s11011-022-00985-8

[34] Kloosterboer E Funke K . Repetitive transcranial magnetic stimulation recovers cortical map plasticity induced by sensory deprivation due to deafferentiation. J Physiol 2019; 597: 4025–4051. 31145483 10.1113/JP277507 PMC6852264

[35] Zong X Li Y Liu C , et al. Theta-burst transcranial magnetic stimulation promotes stroke recovery by vascular protection and neovascularization. Theranostics 2020; 10: 12090–12110. 33204331 10.7150/thno.51573 PMC7667689

[36] Parthoens J Verhaeghe J Servaes S Miranda A Stroobants S Staelens S . Performance characterization of an actively cooled repetitive transcranial magnetic stimulation coil for the rat. Neuromodulation 2016; 19: 459–468. 26846605 10.1111/ner.12387

[37] Chang SJ Santamaria AJ Sanchez FJ , et al. Deep brain stimulation of midbrain locomotor circuits in the freely moving pig. Brain Stimul 2021; 14: 467–476. 33652130 10.1016/j.brs.2021.02.017 PMC9097921

[38] Boonzaier J Petrov PI Otte WM Smirnov N Neggers SFW Dijkhuizen RM . Design and evaluation of a rodent-specific transcranial magnetic stimulation coil: an in silico and in vivo validation study. Neuromodulation 2020; 23: 324–334. 31353780 10.1111/ner.13025 PMC7216963

[39] Boyd LA Hayward KS Ward NS , et al. Biomarkers of stroke recovery: consensus-based core recommendations from the Stroke Recovery and Rehabilitation Roundtable. Int J Stroke 2017; 12: 480–493. 28697711 10.1177/1747493017714176 PMC6791523

[40] Buetefisch CM Wei L Gu X Epstein CM Yu SP . Neuroprotection of low-frequency repetitive transcranial magnetic stimulation after ischemic stroke in rats. Ann Neurol 2023; 93: 336–347. 36097798 10.1002/ana.26509 PMC10042643

[41] Hildesheim FE Silver AN Dominguez-Vargas AU , et al. Predicting individual treatment response to rTMS for motor recovery after stroke: a review and the CanStim perspective. Front Rehabil Sci 2022; 3: 795335. 36188894 10.3389/fresc.2022.795335 PMC9397689

[42] Van Lieshout ECC van der Worp HB Visser-Meily JMA Dijkhuizen RM . Timing of repetitive transcranial magnetic stimulation onset for upper limb function after stroke: a systematic review and meta-analysis. Front Neurol 2019; 10: 1269. 10.3389/fneur.2019.01269 PMC6901630 31849827

[43] Bönstrup M Krawinkel L Schulz R , et al. Low-frequency brain oscillations track motor recovery in human stroke. Ann Neurol 2019; 86: 853–865. 31604371 10.1002/ana.25615

[44] Hordacre B Moezzi B Ridding MC . Towards targeted brain stimulation in stroke: connectivity as a biomarker of response. J Exp Neurosci 2018; 12: 809060. 10.1177/1179069518809060 PMC6236477 30450005

[45] Di Lazzaro V Pellegrino G Di Pino G , et al. Val66Met BDNF gene polymorphism influences human motor cortex plasticity in acute stroke. Brain Stimul 2015; 8: 92–96. 25241287 10.1016/j.brs.2014.08.006 PMC4813754

[46] Abellaneda-Perez K Martin-Trias P Casse-Perrot C , et al. BDNF Val66Met gene polymorphism modulates brain activity following rTMS-induced memory impairment. Sci Rep 2022; 12: 176. 34997117 10.1038/s41598-021-04175-x PMC8741781

[47] Stinear CM Barber PA Petoe M Anwar S Byblow WD . The PREP algorithm predicts potential for upper limb recovery after stroke. Brain 2012; 135: 2527–2535. 22689909 10.1093/brain/aws146

[48] Hawe RL Scott SH Dukelow SP . Taking proportional out of stroke recovery. Stroke 2018; 50: 204–211. 30580742 10.1161/STROKEAHA.118.023006

[49] Steyerberg EW Harrell FE Jr. Prediction models need appropriate internal, internal-external, and external validation. J Clin Epidemiol 2016; 69: 245–247. 25981519 10.1016/j.jclinepi.2015.04.005 PMC5578404

[50] Collins GS Reitsma JB Altman DG Moons KG . Transparent reporting of a multivariable prediction model for individual prognosis or diagnosis (TRIPOD): the TRIPOD statement. BMJ 2015; 350: g7594. 10.1136/bmj.g7594 25569120

[51] Ali M Ashburn A Bowen A , et al. VISTA-Rehab: a resource for stroke rehabilitation trials. Int J Stroke 2010; 5: 447–452. 21050399 10.1111/j.1747-4949.2010.00485.x

[52] Pallmann P Bedding AW Choodari-Oskooei B , et al. Adaptive designs in clinical trials: why use them, and how to run and report them. BMC Med 2018; 16: 29. 29490655 10.1186/s12916-018-1017-7 PMC5830330

