# Supplementary material for: A translational roadmap for transcranial magnetic and direct current stimulation in stroke rehabilitation: Consensus-based core recommendations from the third stroke recovery and rehabilitation roundtable
Source: Neurorehabil Neural Repair. 2023 Oct 14;38(1):19–29. doi: 10.1177/15459683231209136 (PMC10860359; doi:10.1177/15459683231209136)
Supplement: sj-docx-1-nnr-10.1177_15459683231209136 – Supplemental material for A translational roadmap for transcranial magnetic and direct current stimulation in stroke rehabilitation: Consensus-based core recommendations from the third stroke recovery and rehabilitation roundtable [file sj-docx-1-nnr-10.1177_15459683231209136.docx]

**Supplemental Figures and Tables**

**Figure S1.** Article selection flowchart for review of (a) preclinical NIBS studies and (b) NIBS clinical trials from the last 10 years

a.

*See Appendix 1c,d for search criteria for preclinical studies

**See Appendix 1f for full reference list of included preclinical studies

b.

*See Appendix 1e for search criteria for clinical trials

**See Appendix 1g for full reference list of included preclinical studies

**Figure S2.** Structured decision-making process used for in-person consensus meeting*

| Stage Setting |
| --- |
| 1. Facilitator explains process 2. Present clear outcome requirements 3. Questions to clarify  -  |
| Level 1 (Breakout Groups) |
| 1. Present knowledge gaps by priority 2. Open discussion 3. Develop up to 5 recommendation statement(s) per gap |
| Level 2 (Full Roundtable) |
| 1. Present recommendation statements to full roundtable 2. Consensus vote to identify maximum 3 statements per gap |
| If consensus reached, move to next knowledge gap |
| 1. Identify concerns 2. Open discussion to resolve concerns 3. Consensus vote to identify maximum 3 statements per group |
| Consensus Reached |

* Based on methods from *On Conflict and Consensus A Handbook on Formal Consensus Decision making*by C. T. Lawrence Butler and Amy Rothstein, 1987-2007

**Table S1.** Preclinical and clinical barriers (evidence based and feasibility) and weighted rankings

|  |  |  |
| --- | --- | --- |
| **Preclinical Evidence-Based Barriers** | **Initial Ranking** | **Weighted Re-Ranking*** |
| P1. Mechanistic Understanding of NIBS | 1 | **1.6** |
| P2. Lack of standardized outcome measures | 2 | **3.5** |
| P3. Methodological Issues | 3 | **2.3** |
| P4. Lack of clinically relevant animal models | 4 | **3.8** |
| P5. Lack of statistically powered studies | 5 | **3.4** |
| **Feasibility Barriers** |  |  |
| P6. Funding for systematic mechanistic studies | 1 | **1.3** |
| P7. Study Conduct and Reporting guidelines | 2 | **3.1** |
| P8. Publication of negative findings/data repositories | 3 | **3.1** |
| P9. Standardization / harmonization of protocols | 4 | **2.4** |
|  |  |  |
| **Clinical Evidence-Based Barriers** | **Initial Ranking** | **Weighted Re-Ranking*** |
| C1. Lack of individualized NIBS protocols | 1 | **2.6** |
| C2. Lack of standardized outcome measures | 2 | **3.8** |
| C3. Low sample sizes and statistical power | 3 | **2.3** |
| C4. Methodological Issues | 4 | **2.8** |
| C5. Lack of systematic testing/comparisons | 5 | **4.4** |
| C6. Generalizability of preclinical studies to clinical trials | 6 | **6.4** |
| C7. Evidence that stroke rehab changes care costs | 7 | **5.8** |
| **Feasibility Barriers** |  |  |
| C8. Standardized individualized protocols | 1 | **1.2** |
| C9.Treatment and Reporting guidelines | 2 | **2.2** |
| C10. Training on NIBS techniques and integration into clinical workflow | 3 | **2.5** |

*re-rankings conducted after literature review and synthesis

**Table S2.** Core recommendations for the translation of future NIBS research in stroke recovery

| Evidence gap | Recommendation(s) |
| --- | --- |
| NIBS Mechanisms | Preclinical and clinical studies/trials should:   1. systematically compare stimulation parameters within and across modalities and quantify the effects of these parameters on the cortical target 2. use an evidence-based biological framework for target selection and confirm the intervention effect at the level of the target |
| Methodology | Preclinical and clinical studies/trials should include:   1. pre-registration and use of appropriate patient eligibility criteria, blinding and sham stimulation protocols, and appropriate paired therapies 2. conduct and report prospective power analysis to determine samples sizes appropriate to test primary hypotheses 3. use the SRRR3 Unified Checklist for NIBS Research and adhere to current recommended design and reporting guidelines |
| Outcome Standardization | Preclinical studies should:   1. conduct complete outcome assessments across cellular-molecular, physiological and behavioural domains 2. include standardized behavioural outcomes common to human studies   Clinical studies/trials should:   1. use standardized assessments with established psychometric properties 2. report the minimally important clinical difference for outcomes that align with ICF categories and/or study hypotheses |
| Clinically Relevant Preclinical Models | Preclinical studies should use stroke animal models that:   1. include head-equipment size relationship, aged animals, comorbidities, behavioural assessments, and the clinical trajectory of recovery in humans |
| Optimized and Individualized NIBS Protocols | Preclinical and clinical studies should:   1. test multi-domain NIBS response 2. have sufficient sample sizes to identify response phenotypes 3. use appropriate statistical methodology to identify predictive biomarkers |
