# Supplementary material for: A translational roadmap for transcranial magnetic and direct current stimulation in stroke rehabilitation: Consensus-based core recommendations from the third stroke recovery and rehabilitation roundtable
Source: Neurorehabil Neural Repair. 2023 Oct 14;38(1):19–29. doi: 10.1177/15459683231209136 (PMC10860359; doi:10.1177/15459683231209136)
Supplement: sj-docx-2-nnr-10.1177_15459683231209136 – Supplemental material for A translational roadmap for transcranial magnetic and direct current stimulation in stroke rehabilitation: Consensus-based core recommendations from the third stroke recovery and rehabilitation roundtable [file sj-docx-2-nnr-10.1177_15459683231209136.docx]

**Supplemental Appendices**

**Appendix 1a. Description of 5-Stage Nominal Group Consensus Methodology**

*Priority Setting:* Two surveys were developed and circulated electronically (Qualtrics,Version 1.3, Provo, Utah). The first was a thematic idea generation survey (Supplemental Appendix 1b) to identify up to 10 evidence gaps/flaws, barriers and needs for the translation of preclinical and clinical NIBS research. For this consensus exercise, “preclinical” was defined as studies involving animal stroke models and healthy adult participants and “clinical” as studies and/or trials involving stroke participants. Because our group comprised fewer preclinical animal modeling experts, an additional three external members contributed to the idea generation survey (See Acknowledgements). For each question, responses were collated into the top 10 items/concepts for preclinical and clinical, respectively.

*Ranking Survey*: Expert members were then provided with a ranking survey to rank the top 10 items separately according to *priority* and *feasibility to address* and the top 5 ranked preclinical and clinical high priority and high feasibility items were identified (Supplemental Table 1).

*Literature Review:* To ensure consensus recommendations reflected current knowledge and prevent unconscious bias, we conducted a review of recent (last 10 years) preclinical and clinical NIBS research. A medical librarian (SV) conducted three Medline searches to identify: a) preclinical (animal) studies involving NIBS in a stroke model; b) reviews/meta-analyses involving NIBS in healthy adult participants; and c) randomized clinical trials (RCTs) involving NIBS and stroke participants (Supplemental Appendices 1c-e). The expert panel was split into 2 working groups (preclinical and clinical). Articles were screened and all duplicates and those not meeting inclusion criteria were removed (Supplemental Figure 1). Each reviewer was assigned a subset of included studies (Supplemental Appendix 1f-g) and used a data extraction tool to identify key study features, based on the priority and feasibility rankings. Literature extracts for each group were synthesized and presented in a summary meeting to the full expert panel.

*Re-Ranking Survey:* Expert members were then provided with a 2^nd^ ranking survey to re-rank the top 10 items separately according to *priority* and *feasibility to address*. Weighted rankings were then calculated to identify the top 5 preclinical and clinical high priority and high feasibility items to guide the in-person discussion (Supplemental Table 1).

*Consensus Meeting:* A 2-day consensus meeting was held in conjunction with the World Congress of Neurorehabilitation meeting in Vienna, Austria in December 2022. Consensus was achieved via a structured decision-making process (Supplemental Figure 2) and voting to identify a maximum of 3 recommendations per priority item. Feasibility considerations for each recommendation were discussed separately. In-person consensus recommendations were then combined to reduce overlap across preclinical and clinical working groups.

**Appendix 1b. Item Generation Survey**

NIBS Roundtable Survey #1 (Item Generation Survey)

Preamble and Instructions

Thank you for participating in our survey to identify the NIBS Roundtable consensus topics (Survey #1). Please **READ THE FOLLOWING PREAMBLE/INSTRUCTIONS CAREFULLY** prior to completing the survey:

**Preamble:**

- The goal of this survey (Survey #1 – Topics) is to identify the most relevant evidence gaps/flaws, barriers and needs for the translation of preclinical and clinical NIBS research into clinical practice and the role of NIBS biomarkers.
- This survey will be circulated to ALL core members of the NIBS roundtable and an additional 3 leading preclinical researchers in the field.

**Instructions:**

- Please answer each question, providing **UP TO** (but not necessarily) 10 responses to each question OR a response of N/A if the question is outside your area of expertise.

Q1 Please identify **up to** 10 evidence gaps and/or flaws in in-vivo (animal and human) preclinical NIBS research that have limited translation into guideline changing clinical research in stroke patient populations.

- Click to write Form Field 1 (1) ________________________________________________
- Click to write Form Field 2 (2) ________________________________________________
- Click to write Form Field 3 (3) ________________________________________________
- Click to write Form Field 4 (4) ________________________________________________
- Click to write Form Field 5 (5) ________________________________________________
- Click to write Form Field 6 (6) ________________________________________________
- Click to write Form Field 7 (7) ________________________________________________
- Click to write Form Field 8 (8) ________________________________________________
- Click to write Form Field 9 (9) ________________________________________________
- Click to write Form Field 10 (10) ________________________________________________

End of Block: Default Question Block

Q2 Please identify **up to** 10 barriers (logistic, paradigmatic, infrastructure or otherwise) limiting the translation of evidence from in-vivo (animal and human) preclinical NIBS research into guideline changing clinical research in stroke patient populations.

- Click to write Form Field 1 (1) ________________________________________________
- Click to write Form Field 2 (2) ________________________________________________
- Click to write Form Field 3 (3) ________________________________________________
- Click to write Form Field 4 (4) ________________________________________________
- Click to write Form Field 5 (5) ________________________________________________
- Click to write Form Field 6 (6) ________________________________________________
- Click to write Form Field 7 (7) ________________________________________________
- Click to write Form Field 8 (8) ________________________________________________
- Click to write Form Field 9 (9) ________________________________________________
- Click to write Form Field 10 (10) ________________________________________________

End of Block: Default Question Block

Q3 Please identify **up to** 10 needs (logistic, paradigmatic, infrastructure or otherwise) that are required to improve the translation of in-vivo (animal and human) preclinical NIBS data into guideline changing clinical research in stroke patient populations.

- Click to write Form Field 1 (1) ________________________________________________
- Click to write Form Field 2 (2) ________________________________________________
- Click to write Form Field 3 (3) ________________________________________________
- Click to write Form Field 4 (4) ________________________________________________
- Click to write Form Field 5 (5) ________________________________________________
- Click to write Form Field 6 (6) ________________________________________________
- Click to write Form Field 7 (7) ________________________________________________
- Click to write Form Field 8 (8) ________________________________________________
- Click to write Form Field 9 (9) ________________________________________________
- Click to write Form Field 10 (10) ________________________________________________

End of Block: Default Question Block

Q4 Please identify **up to** 10 evidence gaps and/or flaws of clinical NIBS research in stroke patient populations that has limited its translation into clinical practice.

- Click to write Form Field 1 (1) ________________________________________________
- Click to write Form Field 2 (2) ________________________________________________
- Click to write Form Field 3 (3) ________________________________________________
- Click to write Form Field 4 (4) ________________________________________________
- Click to write Form Field 5 (5) ________________________________________________
- Click to write Form Field 6 (6) ________________________________________________
- Click to write Form Field 7 (7) ________________________________________________
- Click to write Form Field 8 (8) ________________________________________________
- Click to write Form Field 9 (9) ________________________________________________
- Click to write Form Field 10 (10) ________________________________________________

End of Block: Default Question Block

Q5 Please identify **up to** 10 barriers (logistic, paradigmatic, infrastructure or otherwise) limiting the translation of evidence from clinical NIBS research in stroke patient populations into clinical practice.

- Click to write Form Field 1 (1) ________________________________________________
- Click to write Form Field 2 (2) ________________________________________________
- Click to write Form Field 3 (3) ________________________________________________
- Click to write Form Field 4 (4) ________________________________________________
- Click to write Form Field 5 (5) ________________________________________________
- Click to write Form Field 6 (6) ________________________________________________
- Click to write Form Field 7 (7) ________________________________________________
- Click to write Form Field 8 (8) ________________________________________________
- Click to write Form Field 9 (9) ________________________________________________
- Click to write Form Field 10 (10) ________________________________________________

End of Block: Default Question Block

Q6 Please identify **up to** 10 needs (logistic, paradigmatic, infrastructure or otherwise) required to improve the translation of clinical NIBS research in stroke patient populations into clinical practice.

- Click to write Form Field 1 (1) ________________________________________________
- Click to write Form Field 2 (2) ________________________________________________
- Click to write Form Field 3 (3) ________________________________________________
- Click to write Form Field 4 (4) ________________________________________________
- Click to write Form Field 5 (5) ________________________________________________
- Click to write Form Field 6 (6) ________________________________________________
- Click to write Form Field 7 (7) ________________________________________________
- Click to write Form Field 8 (8) ________________________________________________
- Click to write Form Field 9 (9) ________________________________________________
- Click to write Form Field 10 (10) ________________________________________________

End of Block: Default Question Block

Q7 Please identify **up to** 10 biomarkers (blood-based, imaging, neurophysiological etc.) that are relevant for preclinical and clinical NIBS research. If you don't consider biomarkers relevant, please state N/A.

- Click to write Form Field 1 (1) ________________________________________________
- Click to write Form Field 2 (2) ________________________________________________
- Click to write Form Field 3 (3) ________________________________________________
- Click to write Form Field 4 (4) ________________________________________________
- Click to write Form Field 5 (5) ________________________________________________
- Click to write Form Field 6 (6) ________________________________________________
- Click to write Form Field 7 (7) ________________________________________________
- Click to write Form Field 8 (8) ________________________________________________
- Click to write Form Field 9 (9) ________________________________________________
- Click to write Form Field 10 (10) ________________________________________________

End of Block: Default Question Block

**Appendix 1c. Search Strategy for Preclinical (Animal) NIBS Research**

**Non-Invasive Brain Stimulation for Stroke in Pre-Clinical Research**

September 27, 2022

| **Searcher: Sarah Visintini**  **Contact:** [**berkmanlibrary@ottawaheart.ca**](mailto:berkmanlibrary@ottawaheart.ca) | **Requestor:** Dr. Jodi Edwards  **Contact:** [**jedwards@ottawaheart.ca**](mailto:jedwards@ottawaheart.ca) |
| --- | --- |
| **Database(s) searched:** Medline  **Limits:** Animal studies only, Last 10 years, Last 5 years, Reviews only  **Search question:** non-invasive brain stimulation in preclinical literature (preclinical = animals + humans non-clinical samples) | |
|  | |

**If a full text article is not free or available to you, please contact me at** [***berkmanlibrary@ottawaheart.ca***](mailto:berkmanlibrary@ottawaheart.ca)

Contents

[Search Results (237) 2](#_Toc115171161)

[Search Results (162) 2](#_Toc115171162)

[Search History 2](#_Toc115171163)

[Accessing Articles 8](#_Toc115171164)

[Affiliation 8](#_Toc115171165)

[Finding Articles 9](#_Toc115171166)

# Search Results (162)

Limited to last 5 years (all publication types)

2022-09-27-LitSearch-Edwards-PreClin-162.xls

2022-09-27-LitSearch-Edwards-PreClin-162.ris

# Search Results (237)

Limited to last 10 years (all publication types)

2022-09-27-LitSearch-Edwards-PreClin-237.xls

2022-09-27-LitSearch-Edwards-PreClin-237.ris

# Search History

Database(s): **Ovid MEDLINE(R) ALL**1946 to September 26, 2022
Search Strategy:

| **#** | **Searches** | **Results** |
| --- | --- | --- |
| 1 | deep brain stimulation/ | 10688 |
| 2 | ((transcranial or brain) adj3 stimulat*).ti. | 22087 |
| 3 | (TMS or TDCS or TACS or NIBS).ti. | 3822 |
| 4 | or/1-3 | 29619 |
| 5 | exp stroke/ | 163726 |
| 6 | (stroke or cerebrovasc$ or brain vasc$ or cerebral vasc$ or cva$ or apoplex$).tw. | 345614 |
| 7 | ((brain$ or cerebr$ or cerebell$ or vertebrobasilar or hemispher$ or intracran$ or intracerebral or infratentorial or supratentorial or MCA or anterior circulation or posterior circulation or basal ganglia) adj3 (isch?emi$ or infarct$ or thrombo$ or emboli$)).tw. | 103186 |
| 8 | ((brain$ or cerebr$ or cerebell$ or intracerebral or intracran$ or parenchymal or intraventricular or infratentorial or supratentorial or basal gangli$) adj3 (haemorrhage$ or hemorrhage$ or haematoma$ or hematoma$ or bleed$)).tw. | 59117 |
| 9 | or/5-8 | 467681 |
| 10 | 4 and 9 | 1985 |
| 11 | exp animal experimentation/ or exp models, animal/ or Animals/ or exp animal population groups/ or chordata/ or vertebrates/ or exp amphibians/ or exp birds/ or exp fishes/ or exp reptiles/ or mammals/ or primates/ or eutheria/ or exp artiodactyla/ or exp carnivora/ or exp cephalopoda/ or exp cetacea/ or chiroptera/ or elephants/ or hyraxes/ or exp eulipotyphla/ or exp lagomorpha/ or exp marsupialia/ or exp monotremata/ or exp perissodactyla/ or proboscidea mammal/ or exp rodentia/ or exp scandentia/ or exp sirenia/ or exp xenarthra/ or haplorhini/ or exp strepsirhini/ or exp platyrrhini/ or exp tarsii/ or catarrhine/ or exp cercopithecidae/ or exp hylobatidae/ or hominidae/ or gorilla gorilla/ or pan paniscus/ or pan troglodytes/ or exp pongo/ or (rat or rats or animal or animals or mice or in vivo or mouse or rabbit or rabbits or murine or pig or pigs or dog or dogs or bovine or fish or vertebrate or vertebrates or cat or cats or rodent or rodents or mammal or mammals or chicken or chickens or monkey or monkeys or sheep or canine or canines or porcine or cattle or bird or birds or hamster or hamsters or primate or primates or cow or cows or chick or horse or horses or avian or avians or calf or swine or swines or xenopus or turkeys or bear or bears or frog or frogs or zebrafish or goat or goats or equine or calves or poultry or macaque or macaques or mole or moles or ovine or lamb or lambs or fishes or diptera or amphibian or amphibians or snake or snakes or ruminant or ruminants or hen or hens or piglet or piglets or feline or felines or simian or simians or laevis or trout or trouts or teleost or teleosts or salmon or salmons or seal or seals or bull or bulls or ewe or ewes or hedgehog or hedgehogs or macaca or macacas or proteus or pigeon or pigeons or bat or bats or duck or ducks or chimpanzee or chimpanzees or baboon or baboons or deer or rana or ranas or carp or carps or heifer or swallow or swallows or lizard or lizards or canis or sow or sows or cynomolgus or quail or quails or reptile or reptiles or turtle or turtles or buffalo or gerbil or gerbils or boar or boars or squirrel or squirrels or oncorhynchus or mus or toad or toads or fowl or fowls or rerio or danio or ara or aras or musculus or tadpole or tadpoles or mulatta or salmo or ram or eagle or eagles or ferret or ferrets or goldfish or catfish or whale or whales or fox or foxes or ape or apes or elephant or elephants or bos or marmoset or marmosets or cod or cods or shark or sharks or wolf or eel or eels or auratus or rattus or zebra or zebras or tilapia or tilapias or gilt or camel or camels or squid or gallus or marsupial or marsupials or vole or voles or fascicularis or ovis or salmonid or salmonids or tiger or tigers or dolphin or dolphins or robin or robins or carpio or opossum or opossums or cyprinus or salamander or salamanders or felis or mink or minks or swan or swans or norvegicus or bufo or torpedo or bass or lamprey or lampreys or sus or python or pythons or tetrapod or tetrapods or shrew or shrews or lion or lions or hog or hogs or songbird or songbirds or oreochromis or starling or starlings or caprine or carassius or owl or owls or newt or newts or papio or scrofa or hare or hares or gorilla or gorillas or flounder or flounders or goose or herring or herrings or therian or buffaloes or canary or sparrow or sparrows or microtus or octopus or troglodytes or tuna or amphibia or chinchilla or chinchillas or ide or oryzias or cervus or kangaroo or kangaroos or armadillo or armadillos or callithrix or pan troglodytes or saimiri or cichlid or cichlids or donkey or donkeys or bream or char or chars or finch or raccoon or raccoons or bothrops or anguilla or perch or cricetus or seabird or seabirds or buck or bucks or naja or coturnix or salmonids or geese or minnow or minnows or raptor or raptors or merione or meriones or rodentia or elaphus or amniote or amniotes or elasmobranch or emu or emus or peromyscus or hominid or hominids or bubalus or crotalus or gull or gulls or anas or anura or lemur or lemurs or crow or crows or camelus or gibbon or gibbons or waterfowl or parrot or parrots or eels or cob or stickleback or sticklebacks or columba or mesocricetus or ambystoma or raven or ravens or gadus or penguin or penguins or orangutan or orangutans or sturgeon or sturgeons or cuniculus or aves or virginianus or cephalopod or cephalopods or cebus or sparus or tortoise or tortoises or guttata or morhua or unguiculatus or dogfish or vulpes or mallard or mallards or apodemus or alligator or alligators or oryctolagus or llama or llamas or reindeer or mustela or duckling or ducklings or wolves or sander or amazona or zebu or badger or badgers or dove or doves or ictalurus or capra or capras or equus or camelid or camelids or poecilia or mule or mules or perciformes or salvelinus or labrax or cyprinidae or ariidae or crocodile or crocodiles or fundulus or dicentrarchus or clarias or cercopithecus or chiroptera or alpaca or alpacas or pike or pikes or paralichthys or puma or pumas or didelphis or pisces or macropus or triturus or bison or bisons or epinephelus or gasterosteus or panthera or acipenser or mackerel or mackerels or tamarin or tamarins or ostrich or anolis or vervet or vervets or wallaby or glareolus or beaver or beavers or dromedary or catus or killifish or pimephales or promelas or aotus or phoca or panda or pandas or porpoise or porpoises or myotis or yak or yaks or agkistrodon or vipera or otter or otters or turbot or turbots or squamate or carnivora or mullet or mullets or hawk or hawks or taeniopygia or seahorse or seahorses or poecilia reticulata or falcon or falcons or prosimian or prosimians or parus or perca or fingerling or fingerlings or antelope or antelopes or tupaia or passeriformes or sepia or saguinus or coyote or coyotes or pongo or meleagris or reptilia or lepus or psittacine or hagfish or warbler or warblers or russell's viper or russell's vipers or smolt or smolts or budgerigar or sardine or sardines or cavia or cavias or hyla or pleurodeles or siluriformes or great tit or great tits or guppy or bonobo or bonobos or rutilus or trichosurus or muridae or phodopus or channa or squalus or lynx or sturnus or petromyzon or vitulina or monodelphis or cuttlefish or adder or adders or lepomis or canaria or gambusia or guppies or xiphophorus or flatfish or koala or koalas or labeo or stingray or stingrays or chelonia or lampetra or spermophilus or crocodilian or passer domesticus or sciurus or artiodactyla or ranidae or corvus or necturus or platypus or canaries or bovid or lagopus or trimeresurus or gariepinus or marten or martens or drosophilidae or mugil or sunfish or porcellus or cypriniformes or alouatta or scophthalmus or anser or electrophorus or putorius or iguana or iguanas or lama or lamas or takifugu or circus or eptesicus or flycatcher or galago or galagos or trachemys or lungfish or characiformes or shorebird or shorebirds or giraffe or giraffes or micropterus or scyliorhinus or cichlidae or loligo or porcupine or porcupines or chub or chubs or solea or pleuronectes or hylidae or viperidae or echis or sorex or anchovy or lagomorph or ostriches or vulture or vultures or whitefish or araneus or jird or jirds or tern or esox or drake or drakes or elapidae or gallopavo or chordata or myodes or caretta or serinus or grouse or misgurnus or meles or blackbird or blackbirds or coregonus or bobwhite or bobwhites or heteropneustes or mammoth or mammoths or turdus or rhinella or ateles or characidae or clupea or bungarus or brill or struthio camelus or sloth or sloths or pteropus or sculpin or anthropoids or pollock or pollocks or morone or pan paniscus or litoria or chipmunk or chipmunks or balaenoptera or marmota or melopsittacus or hyrax or lemming or lemmings or halibut or hylobates or lates or caiman or caimans or sigmodon or stenella or barbel or barbels or sterna or parakeet or parakeets or phocoena or leptodactylus or canidae or buteo or harengus or gopher or gophers or marmot or marmots or gosling or goslings or platichthys or gar or gars or sebastes or marsupialia or notophthalmus or gazelle or gazelles or insectivora or paridae or felidae or russula or galliformes or bombina or colobus or echidna or echidnas or seabass or syncerus or plaice or blue tit or blue tits or pagrus or catfishes or cetacea or barbus or cygnus or ficedula or chamois or colubridae or perches or coelacanth or fitch or urodela or cynops or martes or halichoerus or aix or salmonidae or leuciscus or magpie or magpies or silurus or whiting or whitings or anseriformes or colinus or rhea or chlorocebus or octodon or acinonyx or mouflon or mouflons or ibex or tetraodon or bufonidae or equidae or jackal or cephalopoda or dendroaspis or glama or muskrat or muskrats or sable or sables or wildebeest or streptopelia or albifrons or vespertilionidae or woodpecker or woodpeckers or muntjac or muntjacs or archosaur or branta or cricetulus or megalobrama or poeciliidae or desmodus or snakehead or snakeheads or tench or teal or teals or bandicoot or bandicoots or apteronotus or phyllostomidae or crocidura or buzzard or buzzards or larimichthys or cercocebus or pipistrellus or erithacus or impala or impalas or rousettus or haddock or haddocks or tinca or ratite or calidris or cynoglossus or hypophthalmichthys or bullock or bullocks or dromedaries or alectoris or filly or salamandra or cingulata or bitis or grus or ammodytes or macaw or macaws or hypoleuca or sapajus or cyprinodontiformes or hippopotamus or pelophylax or capybara or capybaras or weasel or weasels or cairina or cynomys or lutra or cockatoo or cockatoos or lachesis or lagomorpha or rupicapra or daboia or orang utan or orang utans or platyrrhini or charadriiformes or micrurus or psittaciformes or spalax or loris or mustelidae or sylvilagus or vitticeps or cockatiel or mustelus or cottus or erythrocebus or dipodomys or platessa or callicebus or loricariidae or catostomus or cuneata or cyanistes or cyprinodon or sigmodontinae or elasmobranchii or trichechus or sauropsid or xenarthra or dormouse or perissodactyla or nautilus or cirrhinus or gulo or tragelaphus or merula or numida or sciaenidae or cerastes or sciuridae or gibbosus or octopuses or eland or elands or phyllomedusa or pogona or walrus or agamidae or leptodactylidae or ridibundus or leontopithecus or anteater or anteaters or pelodiscus or cebidae or columbianus or pelteobagrus fulvidraco or hominoidea or mandrillus or zonotrichia leucophrys or agama or gobiocypris or bearded dragon or bearded dragons or sarotherodon or talpa or discoglossus or hagfishes or sphenodon or gudgeon or amphiuma or aythya or tenrec or tenrec or hominidae or risoria or salamandridae or camelidae or columbiformes or latimeria or plover or plovers or afrotheria or falco sparverius or polecat or polecats or crotalinae or salvadora or tarsier or lucioperca or anchovies or lungfishes or terrapin or dromaius novaehollandiae or lateolabrax or eigenmannia or pelamis or theropithecus or murinae or gander or gymnotus or pseudacris or gymnophiona or gymnotiformes or laticauda or falconiformes or dugong or dugongs or pintail or pintails or rook or rooks or lasiurus or catshark or catsharks or micropogonias or red junglefowl or paddlefish or ophiophagus or hollandicus or nymphicus or pimelodidae or aepyceros or cobitidae or strigiformes or cobitis or dormice or alytes or calloselasma or guanaco or phasianidae or round goby or trichogaster or catarrhini or eelpout or eelpouts or galaxias or gaur or pungitius or suslik or susliks or flatfishes or percidae or caprinae or todarodes or osmerus or ameiurus or anthropoidea or castor canadensis or pouting or poutings or tetraodontiformes or arvicolinae or siamang or siamangs or castor fiber or nomascus or red knot or red knots or syngnathidae or iguanidae or eretmochelys or ursidae or callimico or columbidae or microhylidae or anaxyrus or menidia or pipistrelle or greylag or pipidae or scandentia or bowfin or bowfins or dendrobatidae or zenaida or bushbaby or harrier or harriers or macropodidae or pygerythrus or clupeidae or odorrana or corvidae or jerboa or jerboas or canutus or hylobatidae or clupeiformes or great cormorant or great cormorants or scorpaeniformes or chondrostean or garfish or proboscidea or psetta or diapsid or serotinus or tetrao or walruses or carcharhiniformes or leucoraja or pumpkinseed or dosidicus or acipenseriformes or daubentonii or emberizidae or gadiformes or hyraxes or stizostedion or wolverine or wolverines or lissotriton or acanthurus or centrarchidae or gloydius or laurasiatheria or limosa or psittacula or leporidae or proteidae or zander or zanders or arapaima or bagridae or cyprinodontidae or mithun or pandion or jackdaw or jackdaws or procyonidae or carus or jaculus or salmoniformes or common sole or common soles or protobothrops or calamita or brachyteles or trionyx or turdidae or boidae or luscinia or pugnax or euarchontoglires or saithe or saithes or symphalangus or aardvark or aardvarks or oystercatcher or oystercatchers or arius or corydoras or poacher or poachers or aurochs or cebuella or crecca or lemuridae or sirenia or lemmus or perdix or glires or lepidosaur or muskox or deinagkistrodon or pholidota or holocephali or cercopithecinae or clariidae or agapornis or doryteuthis or tyrannidae or dicroglossidae or godwit or godwits or monedula or pongidae or atheriniformes or colobinae or lophocebus or atelidae or cottidae or leucopsis or acanthuridae or didelphimorphia or elver or elvers or lapponica or dermoptera or european hake or european hakes or gerbillinae or banteng or hartebeest or hartebeests or hogget or haematopus or anguis fragilis or grey heron or grey herons or blue whiting or blue whitings or furnariidae or macrovipera or esocidae or lapwing or lapwings or mylopharyngodon or wallabia or beloniformes or potoroo or potoroos or athene noctua or pleuronectidae or bushbabies or muscicapidae or alligatoridae or fuligula or bush baby or guineafowl or spoonbill or spoonbills or viverridae or catostomidae or zebrafishes or ibexes or vendace or estrildidae or monotremata or sepiella or ambystomatidae or shelduck or shelducks or treeshrew or treeshrews or hoplobatrachus or pochard or hoolock or hoolocks or lynxes or antilope or antilopes or blackbuck or blackbucks or cricetinae or paramisgurnus or skylark or skylarks or soleidae or allobates or northern wheatear or northern wheatears or pitheciidae or takin or theria or vanellus or galaxiidae or lorisidae or ostralegus or palaeognathae or stone loach or alauda or callitrichinae or caniformia or duttaphrynus or ictaluridae or osteoglossiformes or poultries or curema or ruddy turnstone or ruddy turnstones or sheatfish or sunfishes or centropomidae or hemachatus or platalea or thamnophilidae or song thrush or atherinopsidae or siluridae or tadorna or chroicocephalus or ermine or ermines or gavialis or ruff or tupaiidae or diprotodontia or hyaenidae or antilopinae or crocodylidae or herpestidae or hippopotamidae or northern shoveler or round gobies or cheirogaleidae or indriidae or fundulidae or pythonidae or rhynchocephalia or anodorhynchus or red-backed shrike or red-backed shrikes or triakidae or phalangeridae or aoudad or boreoeutheria or eurasian jay or eurasian jays or feliformia or haplorhini or osteoglossidae or paenungulata or struthioniformes or ferina or sanderling or sanderlings or spheniscidae or cuttlefishes or cygnet or dasycneme or gadwall or gadwalls or pelobates fuscus or wryneck or wrynecks or afrosoricida or culaea or dover sole or dover soles or paralichthyidae or passeridae or osteolaemus or song thrushes or bluethroat or bluethroats or hydrophiidae or megrim or mephitidae or strepsirhini or tomistoma or epidalea or osmeriformes or bush babies or tarsiiform or atelinae or bufotes or eurasian coot or eurasian coots or galagidae or geopelia or philomachus or tubulidentata or bombinatoridae or pelobatidae or tachysurus or ailuridae or woodlark or woodlarks or alcelaphinae or redshank or redshanks or salientia or sand smelt or sand smelts or woodmice or woodmouse or dasyproctidae or eurasian wigeon or eurasian wigeons or garganey or garganeys or lemon sole or lemon soles or common dab or common dabs or graylag or graylags or leucorodia or osphronemidae or bewickii or common moorhen or common moorhens or decapodiformes or gobbler or gobblers or odontophoridae or paddlefishes or eutheria or salmonine or esociformes or eurasian woodcock or eurasian woodcocks or european smelt or european smelts or goldfishes or tenches or tyranni or common chaffinch or common chaffinchs or common redstart or common redstarts or common roach or common roachs or great knot or great knots or potoroidae or alytidae or coregonine or dipteral or leveret or poeciliopsis gracilis or amphiumidae or batrachoidiformes or bighead goby or heteropneustidae or lullula or norway pout or norway pouts or sipunculida or dogfishes or sebastidae or tarsiidae or alethinophidia or common nase or common nases or common sandpiper or common sandpipers or eurasian blackcap or eurasian blackcaps or pterocnemia or syngnathiformes or common chaffinches or eupleridae or octopodiformes or phascolarctidae or scophthalmidae or starry smooth-hound or starry smooth-hounds or whitefishes or cuniculidae or european sprat or european sprats or rosy bitterling or rosy bitterlings or common dace or common daces or lesser weever or lesser weevers or scaldfish or water rail or water rails or alouattinae or centrarchiformes or common whitethroat or common whitethroats or gavialidae or grey gurnard or grey gurnards or lateolabracidae or rheiformes or tub gurnard or tub gurnards or common chiffchaff or common chiffchaffs or garfishes or lesser whitethroat or lesser whitethroats or myoxidae or seabasses or spariformes or umbridae or yellow boxfish or anabantiformes or aotidae or common bleak or common bleaks or common rudd or common rudds or greater pipefish or hapale or nandiniidae or stone loaches or whinchat or whinchats or acanthuriformes or brotula barbata or common ling or common lings or common roaches or cottonrat or cottonrats or douroucoulis or dromaiidae or fitches or fitchew or galaxiiformes or laprine or saimiriinae or solenette or tarsii or tompot blenny or common dragonet or common dragonets or longspined bullhead or longspined bullheads or monotremate or monotremates or pempheriformes or perdicinae or presbytini or smegmamorpha or bighead gobies or carangaria incertae sedis or coiidae or fivebeard rockling or foulmart or foumart or grasskeet or greater pipefishes or ibices or millionfish or muguliformes or norwegian topknot or peewit or red sea sailfin tang or rupicapras or sheatfishes or tompot blennies or twait shad or yellow boxfishes).ti,ab,kf. | 8552520 |
| 12 | 10 and 11 | 300 |
| 13 | limit 12 to yr="2011 -Current" | 237 |
| 14 | limit 12 to yr="2016 -Current" | 162 |

**Appendix 1d. Search Strategy for Preclinical (Healthy Adults) NIBS Research**

**Non-Invasive Brain Stimulation in Healthy Volunteers**

September 29, 2022

| **Searcher: Sarah Visintini**  **Contact:** [**berkmanlibrary@ottawaheart.ca**](mailto:berkmanlibrary@ottawaheart.ca) | **Requestor:** Dr. Jodi Edwards  **Contact:** [**jedwards@ottawaheart.ca**](mailto:jedwards@ottawaheart.ca) |
| --- | --- |
| **Database(s) searched:** Medline  **Limits:** Last 10 years, Last 5 years, Reviews only  **Search question:** non-invasive brain stimulation in healthy volunteers | |
|  | |

**If a full text article is not free or available to you, please contact me at** [***berkmanlibrary@ottawaheart.ca***](mailto:berkmanlibrary@ottawaheart.ca)

Contents

[Search Results (157) 2](#_Toc115340657)

[Search History 2](#_Toc115340658)

[Accessing Articles 3](#_Toc115340659)

[Affiliation 3](#_Toc115340660)

[Finding Articles 3](#_Toc115340661)

# Search Results (157)

Limited to reviews/systematic reviews/meta-analyses and last 10 years

2022-09-29-LitSearch-Edwards-HealthyVolunteers-157.xls

2022-09-29-LitSearch-Edwards-HealthyVolunteers-157.ris

# Search History

Database(s): **Ovid MEDLINE(R) ALL**1946 to September 28, 2022
Search Strategy:

| **#** | **Searches** | **Results** |
| --- | --- | --- |
| 1 | deep brain stimulation/ | 10684 |
| 2 | ((transcranial or brain) adj3 stimulat*).ti. | 22085 |
| 3 | (TMS or TDCS or TACS or NIBS).ti. | 3831 |
| 4 | or/1-3 | 29626 |
| 5 | Healthy Volunteers/ | 25142 |
| 6 | (volunteer? or (healthy adj3 (participant* or subject* or patient* or volunteer* or population* or group))).ti,ab. | 467674 |
| 7 | 5 or 6 | 477253 |
| 8 | 4 and 7 | 2642 |
| 9 | meta-analysis.pt. | 168275 |
| 10 | meta-analysis/ or systematic review/ or meta-analysis as topic/ or "meta analysis (topic)"/ or "systematic review (topic)"/ or exp technology assessment, biomedical/ | 317574 |
| 11 | ((systematic* adj3 (review* or overview*)) or (methodologic* adj3 (review* or overview*))).ti,ab,kf,kw. | 286369 |
| 12 | ((quantitative adj3 (review* or overview* or synthes*)) or (research adj3 (integrati* or overview*))).ti,ab,kf,kw. | 14409 |
| 13 | ((integrative adj3 (review* or overview*)) or (collaborative adj3 (review* or overview*)) or (pool* adj3 analy*)).ti,ab,kf,kw. | 35940 |
| 14 | (data synthes* or data extraction* or data abstraction*).ti,ab,kf,kw. | 36985 |
| 15 | (handsearch* or hand search*).ti,ab,kf,kw. | 10713 |
| 16 | (mantel haenszel or peto or der simonian or dersimonian or fixed effect* or latin square*).ti,ab,kf,kw. | 33262 |
| 17 | (met analy* or metanaly* or technology assessment* or HTA or HTAs or technology overview* or technology appraisal*).ti,ab,kf,kw. | 11488 |
| 18 | (meta regression* or metaregression*).ti,ab,kf,kw. | 13156 |
| 19 | (meta-analy* or metaanaly* or systematic review* or biomedical technology assessment* or bio-medical technology assessment*).mp,hw. | 426411 |
| 20 | (medline or cochrane or pubmed or medlars or embase or cinahl).ti,ab,hw. | 310375 |
| 21 | (cochrane or (health adj2 technology assessment) or evidence report).jw. | 21015 |
| 22 | (meta-analysis or systematic review).mp. [mp=title, book title, abstract, original title, name of substance word, subject heading word, floating sub-heading word, keyword heading word, organism supplementary concept word, protocol supplementary concept word, rare disease supplementary concept word, unique identifier, synonyms] | 399066 |
| 23 | (comparative adj3 (efficacy or effectiveness)).ti,ab,kf,kw. | 16563 |
| 24 | (outcomes research or relative effectiveness).ti,ab,kf,kw. | 10785 |
| 25 | ((indirect or indirect treatment or mixed-treatment) adj comparison*).ti,ab,kf,kw. | 2721 |
| 26 | or/9-25 | 627256 |
| 27 | 8 and 26 | 102 |
| 28 | limit 8 to "review articles" | 136 |
| 29 | 27 or 28 | 189 |
| 30 | limit 29 to yr="2012 -Current" | 157 |

**Appendix 1e. Search Strategy for Clinical (Stroke Patients) NIBS Research**

**Non-Invasive Brain Stimulation in Stroke Patients**

October 17, 2022

| **Searcher: Sarah Visintini**  **Contact:** [**berkmanlibrary@ottawaheart.ca**](mailto:berkmanlibrary@ottawaheart.ca) | **Requestor:** Dr. Jodi Edwards  **Contact:** [**jedwards@ottawaheart.ca**](mailto:jedwards@ottawaheart.ca) |
| --- | --- |
| **Database(s) searched:** Medline  **Limits:** Last 10 years, RCTs only  **Search question:** | |
| Non-invasive brain stimulation in clinical population (stroke patients) | |

**If a full text article is not free or available to you, please contact me at** [***berkmanlibrary@ottawaheart.ca***](mailto:berkmanlibrary@ottawaheart.ca)

Contents

[Search Results (XX) 1](#_Toc20992358)

[Search History 1](#_Toc20992359)

# Search Results (829)

2022-10-17-LitSearch-Edwards.RIS

2022-10-17-LitSearch-Edwards.CSV

#

# Search History

Database(s): **Ovid MEDLINE(R) ALL**1946 to October 14, 2022
Search Strategy:

| **#** | **Searches** | **Results** |
| --- | --- | --- |
| 1 | deep brain stimulation/ | 10706 |
| 2 | ((transcranial or brain) adj3 stimulat*).ti,ab,kf. | 45991 |
| 3 | (TMS or TDCS or TACS or NIBS).ti,ab,kf. | 21771 |
| 4 | or/1-3 | 55245 |
| 5 | exp stroke/ | 164244 |
| 6 | (stroke or cerebrovasc$ or brain vasc$ or cerebral vasc$ or cva$ or apoplex$).tw. | 346729 |
| 7 | ((brain$ or cerebr$ or cerebell$ or vertebrobasilar or hemispher$ or intracran$ or intracerebral or infratentorial or supratentorial or MCA or anterior circulation or posterior circulation or basal ganglia) adj3 (isch?emi$ or infarct$ or thrombo$ or emboli$)).tw. | 103424 |
| 8 | ((brain$ or cerebr$ or cerebell$ or intracerebral or intracran$ or parenchymal or intraventricular or infratentorial or supratentorial or basal gangli$) adj3 (haemorrhage$ or hemorrhage$ or haematoma$ or hematoma$ or bleed$)).tw. | 59289 |
| 9 | or/5-8 | 469048 |
| 10 | 4 and 9 | 3647 |
| 11 | randomized controlled trial.pt. | 578616 |
| 12 | controlled clinical trial.pt. | 95068 |
| 13 | randomized.ab. | 578774 |
| 14 | placebo.ab. | 232345 |
| 15 | clinical trials as topic.sh. | 200444 |
| 16 | randomly.ab. | 393304 |
| 17 | trial.ti. | 271902 |
| 18 | 11 or 12 or 13 or 14 or 15 or 16 or 17 | 1478668 |
| 19 | exp animals/ not humans.sh. | 5054549 |
| 20 | 18 not 19 | 1360349 |
| 21 | 10 and 20 | 992 |
| 22 | limit 21 to yr="2012 -Current" | 829 |

**Appendix 1f. Reference list for preclinical studies (animal models and healthy adults) literature review**

1. Aceves-Serrano L, Neva JL, Doudet DJ. Insight Into the Effects of Clinical Repetitive Transcranial Magnetic Stimulation on the Brain From Positron Emission Tomography and Magnetic Resonance Imaging Studies: A Narrative Review. Front Neurosci 2022;16:787403.

2. Agarwal S, Koch G, Hillis AE et al. Interrogating cortical function with transcranial magnetic stimulation: insights from neurodegenerative disease and stroke. J Neurol Neurosurg Psychiatry 2019;90:47-57.

3. Ahn SM, Jung DH, Lee HJ et al. Contralesional Application of Transcranial Direct Current Stimulation on Functional Improvement in Ischemic Stroke Mice. Stroke 2020;51:2208-2218.

4. Ambriz-Tututi M, Sanchez-Gonzalez V, Drucker-Colin R. Transcranial magnetic stimulation reduces nociceptive threshold in rats. J Neurosci Res 2012;90:1085-95.

5. Andrade DC, Borges I, Bravo GL, Bolognini N, Fregni F. Therapeutic time window of noninvasive brain stimulation for pain treatment: inhibition of maladaptive plasticity with early intervention. Expert Rev Med Devices 2013;10:339-52.

6. Antal A, Alekseichuk I, Bikson M et al. Low intensity transcranial electric stimulation: Safety, ethical, legal regulatory and application guidelines. Clin Neurophysiol 2017;128:1774-1809.

7. Antal A, Keeser D, Priori A, Padberg F, Nitsche MA. Conceptual and Procedural Shortcomings of the Systematic Review "Evidence That Transcranial Direct Current Stimulation (tDCS) Generates Little-to-no Reliable Neurophysiologic Effect Beyond MEP Amplitude Modulation in Healthy Human Subjects: A Systematic Review" by Horvath and Co-workers. Brain Stimul 2015;8:846-9.

8. Babiloni C, Del Percio C, Lizio R et al. A review of the effects of hypoxia, sleep deprivation and transcranial magnetic stimulation on EEG activity in humans: challenges for drug discovery for Alzheimer's disease. Curr Alzheimer Res 2014;11:501-18.

9. Baek H, Sariev A, Kim MJ, Lee H, Kim J, Kim H. A neuroprotective brain stimulation for vulnerable cerebellar Purkinje cell after ischemic stroke: a study with low-intensity focused ultrasound. Annu Int Conf IEEE Eng Med Biol Soc 2018;2018:4744-4747.

10. Bahji A, Forth E, Yang CC, Khalifa N. Transcranial direct current stimulation for empathy: A systematic review and meta-analysis. Soc Neurosci 2021;16:232-255.

11. Balboa-Bandeira Y, Zubiaurre-Elorza L, Ibarretxe-Bilbao N, Ojeda N, Pena J. Effects of transcranial electrical stimulation techniques on second and foreign language learning enhancement in healthy adults: A systematic review and meta-analysis. Neuropsychologia 2021;160:107985.

12. Barbati SA, Cocco S, Longo V et al. Enhancing Plasticity Mechanisms in the Mouse Motor Cortex by Anodal Transcranial Direct-Current Stimulation: The Contribution of Nitric Oxide Signaling. Cereb Cortex 2020;30:2972-2985.

13. Bastani A, Jaberzadeh S. Does anodal transcranial direct current stimulation enhance excitability of the motor cortex and motor function in healthy individuals and subjects with stroke: a systematic review and meta-analysis. Clin Neurophysiol 2012;123:644-57.

14. Bates KA, Rodger J. Repetitive transcranial magnetic stimulation for stroke rehabilitation-potential therapy or misplaced hope? Restor Neurol Neurosci 2015;33:557-69.

15. Behrangrad S, Zoghi M, Kidgell D, Jaberzadeh S. Does cerebellar non-invasive brain stimulation affect corticospinal excitability in healthy individuals? A systematic review of literature and meta-analysis. Neurosci Lett 2019;706:128-139.

16. Behrangrad S, Zoghi M, Kidgell D, Jaberzadeh S. The Effect of a Single Session of Non-Invasive Brain Stimulation on Balance in Healthy Individuals: A Systematic Review and Best Evidence Synthesis. Brain Connect 2021;11:695-716.

17. Bennabi D, Pedron S, Haffen E, Monnin J, Peterschmitt Y, Van Waes V. Transcranial direct current stimulation for memory enhancement: from clinical research to animal models. Front Syst Neurosci 2014;8:159.

18. Beom J, Kim W, Han TR, Seo KS, Oh BM. Concurrent use of granulocyte-colony stimulating factor with repetitive transcranial magnetic stimulation did not enhance recovery of function in the early subacute stroke in rats. Neurol Sci 2015;36:771-7.

19. Berryhill ME, Martin D. Cognitive Effects of Transcranial Direct Current Stimulation in Healthy and Clinical Populations: An Overview. J ECT 2018;34:e25-e35.

20. Bikson M, Grossman P, Thomas C et al. Safety of Transcranial Direct Current Stimulation: Evidence Based Update 2016. Brain Stimul 2016;9:641-661.

21. Bikson M, Rahman A, Datta A. Computational models of transcranial direct current stimulation. Clin EEG Neurosci 2012;43:176-83.

22. Block HJ, Celnik P. Can cerebellar transcranial direct current stimulation become a valuable neurorehabilitation intervention? Expert Rev Neurother 2012;12:1275-7.

23. Bohr A, Schuhmann MK, Papp L, Volkmann J, Fluri F. Deep Brain Stimulation for Stroke: Continuous Stimulation of the Pedunculopontine Tegmental Nucleus has no Impact on Skilled Walking in Rats After Photothrombotic Stroke. Curr Neurovasc Res 2020;17:636-643.

24. Boonzaier J, van Tilborg GAF, Neggers SFW, Dijkhuizen RM. Noninvasive Brain Stimulation to Enhance Functional Recovery After Stroke: Studies in Animal Models. Neurorehabil Neural Repair 2018;32:927-940.

25. Bragina OA, Semyachkina-Glushkovskaya OV, Nemoto EM, Bragin DE. Anodal Transcranial Direct Current Stimulation Improves Impaired Cerebrovascular Reactivity in Traumatized Mouse Brain. Adv Exp Med Biol 2020;1232:47-53.

26. Braun R, Klein R, Walter HL et al. Transcranial direct current stimulation accelerates recovery of function, induces neurogenesis and recruits oligodendrocyte precursors in a rat model of stroke. Exp Neurol 2016;279:127-136.

27. Brem AK, Fried PJ, Horvath JC, Robertson EM, Pascual-Leone A. Is neuroenhancement by noninvasive brain stimulation a net zero-sum proposition? Neuroimage 2014;85 Pt 3:1058-68.

28. Brevet-Aeby C, Brunelin J, Iceta S, Padovan C, Poulet E. Prefrontal cortex and impulsivity: Interest of noninvasive brain stimulation. Neurosci Biobehav Rev 2016;71:112-134.

29. Bruckner S, Kammer T. Both anodal and cathodal transcranial direct current stimulation improves semantic processing. Neuroscience 2017;343:269-275.

30. Buetefisch CM, Wei L, Gu X, Epstein CM, Yu SP. Neuroprotection of Low-Frequency Repetitive Transcranial Magnetic Stimulation after Ischemic Stroke in Rats. Ann Neurol 2023;93:336-347.

31. Burke MJ, Fried PJ, Pascual-Leone A. Transcranial magnetic stimulation: Neurophysiological and clinical applications. Handb Clin Neurol 2019;163:73-92.

32. Cao Y, Yin P, Hu X, Ge Y, Zhou X. Chronic high-frequency stimulation therapy in hemiparkinsonian rhesus monkeys using an implanted human DBS system. Neurol Sci 2013;34:707-14.

33. Cavaleri R, Schabrun SM, Chipchase LS. The number of stimuli required to reliably assess corticomotor excitability and primary motor cortical representations using transcranial magnetic stimulation (TMS): a systematic review and meta-analysis. Syst Rev 2017;6:48.

34. Chan HH, Cooperrider J, Chen Z et al. Lateral Cerebellar Nucleus Stimulation has Selective Effects on Glutamatergic and GABAergic Perilesional Neurogenesis After Cortical Ischemia in the Rodent Model. Neurosurgery 2018;83:1057-1067.

35. Chan HH, Wathen CA, Mathews ND et al. Lateral cerebellar nucleus stimulation promotes motor recovery and suppresses neuroinflammation in a fluid percussion injury rodent model. Brain Stimul 2018;11:1356-1367.

36. Charalambous CC, Bowden MG, Adkins DL. Motor Cortex and Motor Cortical Interhemispheric Communication in Walking After Stroke: The Roles of Transcranial Magnetic Stimulation and Animal Models in Our Current and Future Understanding. Neurorehabil Neural Repair 2016;30:94-102.

37. Cheng J, Fan YQ, Jiang HX et al. Transcranial direct-current stimulation protects against cerebral ischemia-reperfusion injury through regulating Cezanne-dependent signaling. Exp Neurol 2021;345:113818.

38. Cheng X, Li T, Zhou H et al. Cortical electrical stimulation with varied low frequencies promotes functional recovery and brain remodeling in a rat model of ischemia. Brain Res Bull 2012;89:124-32.

39. Cherchi L, Anni D, Buffelli M, Cambiaghi M. Early Application of Ipsilateral Cathodal-tDCS in a Mouse Model of Brain Ischemia Results in Functional Improvement and Perilesional Microglia Modulation. Biomolecules 2022;12.

40. Chou W, Liu YF, Lin CH et al. Exercise Rehabilitation Attenuates Cognitive Deficits in Rats with Traumatic Brain Injury by Stimulating the Cerebral HSP20/BDNF/TrkB Signalling Axis. Mol Neurobiol 2018;55:8602-8611.

41. Clayton E, Kinley-Cooper SK, Weber RA, Adkins DL. Brain stimulation: Neuromodulation as a potential treatment for motor recovery following traumatic brain injury. Brain Res 2016;1640:130-138.

42. Cocco S, Podda MV, Grassi C. Role of BDNF Signaling in Memory Enhancement Induced by Transcranial Direct Current Stimulation. Front Neurosci 2018;12:427.

43. Coffman BA, Clark VP, Parasuraman R. Battery powered thought: enhancement of attention, learning, and memory in healthy adults using transcranial direct current stimulation. Neuroimage 2014;85 Pt 3:895-908.

44. Corp DT, Bereznicki HGK, Clark GM et al. Large-scale analysis of interindividual variability in theta-burst stimulation data: Results from the 'Big TMS Data Collaboration'. Brain Stimul 2020;13:1476-1488.

45. Corp DT, Lum JA, Tooley GA, Pearce AJ. Corticospinal activity during dual tasking: a systematic review and meta-analysis of TMS literature from 1995 to 2013. Neurosci Biobehav Rev 2014;43:74-87.

46. Cui J, Kim CS, Kim Y, Sohn MK, Jee S. Effects of Repetitive Transcranial Magnetic Stimulation (rTMS) Combined with Aerobic Exercise on the Recovery of Motor Function in Ischemic Stroke Rat Model. Brain Sci 2020;10.

47. Cui M, Ge H, Zeng H et al. Repetitive Transcranial Magnetic Stimulation Promotes Neural Stem Cell Proliferation and Differentiation after Intracerebral Hemorrhage in Mice. Cell Transplant 2019;28:568-584.

48. D'Agata F, Cicerale A, Mingolla A et al. Double-Cone Coil TMS Stimulation of the Medial Cortex Inhibits Central Pain Habituation. PLoS One 2015;10:e0128765.

49. de Moura M, Hazime FA, Marotti Aparicio LV, Grecco LAC, Brunoni AR, Hasue RH. Effects of transcranial direct current stimulation (tDCS) on balance improvement: a systematic review and meta-analysis. Somatosens Mot Res 2019;36:122-135.

50. Dedoncker J, Brunoni AR, Baeken C, Vanderhasselt MA. The effect of the interval-between-sessions on prefrontal transcranial direct current stimulation (tDCS) on cognitive outcomes: a systematic review and meta-analysis. J Neural Transm (Vienna) 2016;123:1159-72.

51. Dedoncker J, Brunoni AR, Baeken C, Vanderhasselt MA. A Systematic Review and Meta-Analysis of the Effects of Transcranial Direct Current Stimulation (tDCS) Over the Dorsolateral Prefrontal Cortex in Healthy and Neuropsychiatric Samples: Influence of Stimulation Parameters. Brain Stimul 2016;9:501-17.

52. Deng LD, Qi L, Suo Q et al. Transcranial focused ultrasound stimulation reduces vasogenic edema after middle cerebral artery occlusion in mice. Neural Regen Res 2022;17:2058-2063.

53. Deng Y, Guo F, Han X, Huang X. Repetitive transcranial magnetic stimulation increases neurological function and endogenous neural stem cell migration via the SDF-1alpha/CXCR4 axis after cerebral infarction in rats. Exp Ther Med 2021;22:1037.

54. Dias IA, Hazime FA, Lopes DA, Silva CSD, Baptista AF, Silva B. Effects of transcranial direct current stimulation on heart rate variability: a systematic review protocol. JBI Evid Synth 2020;18:1313-1319.

55. Dissanayaka T, Zoghi M, Farrell M, Egan GF, Jaberzadeh S. Does transcranial electrical stimulation enhance corticospinal excitability of the motor cortex in healthy individuals? A systematic review and meta-analysis. Eur J Neurosci 2017;46:1968-1990.

56. Duan JX, Zheng MG, Mu SH et al. Transcranial direct current stimulation treated by multilead brain reflex instrument accelerates neural functional recovery in a rat model of stroke. Int J Neurosci 2021;131:571-579.

57. Faillot M, Chaillet A, Palfi S, Senova S. Rodent models used in preclinical studies of deep brain stimulation to rescue memory deficits. Neurosci Biobehav Rev 2021;130:410-432.

58. Feczko T, Piiper A, Ansar S et al. Stimulating brain recovery after stroke using theranostic albumin nanocarriers loaded with nerve growth factor in combination therapy. J Control Release 2019;293:63-72.

59. Feng LB, Pang XM, Zhang L et al. MicroRNA involvement in mechanism of endogenous protection induced by fastigial nucleus stimulation based on deep sequencing and bioinformatics. BMC Med Genomics 2015;8:79.

60. Ferrucci R, Cortese F, Priori A. Cerebellar tDCS: how to do it. Cerebellum 2015;14:27-30.

61. Fetz EE. Restoring motor function with bidirectional neural interfaces. Prog Brain Res 2015;218:241-52.

62. Ficarella SC, Battelli L. Motor Preparation for Action Inhibition: A Review of Single Pulse TMS Studies Using the Go/NoGo Paradigm. Front Psychol 2019;10:340.

63. Floyd JT, Lairamore C, Garrision MK et al. Transcranial Direct Current Stimulation (tDCS) Can Alter Cortical Excitability of the Lower Extremity in Healthy Participants: A Review and Methodological Study. Front Neurol Neurosci Res 2020;1.

64. Fluri F, Malzahn U, Homola GA, Schuhmann MK, Kleinschnitz C, Volkmann J. Stimulation of the mesencephalic locomotor region for gait recovery after stroke. Ann Neurol 2017;82:828-840.

65. Fluri F, Mutzel T, Schuhmann MK, Krstic M, Endres H, Volkmann J. Development of a head-mounted wireless microstimulator for deep brain stimulation in rats. J Neurosci Methods 2017;291:249-256.

66. Fong PY, Chuang WY, Huang YZ, Chang CH. Safety of carotid artery stent in repetitive transcranial magnetic stimulation-The histopathological proof from swine carotid artery. Neurosci Lett 2017;657:194-198.

67. Forcano L, Mata F, de la Torre R, Verdejo-Garcia A. Cognitive and neuromodulation strategies for unhealthy eating and obesity: Systematic review and discussion of neurocognitive mechanisms. Neurosci Biobehav Rev 2018;87:161-191.

68. Frohlich F, Sellers KK, Cordle AL. Targeting the neurophysiology of cognitive systems with transcranial alternating current stimulation. Expert Rev Neurother 2015;15:145-67.

69. Fusco G, Cristiano A, Perazzini A, Aglioti SM. Neuromodulating the performance monitoring network during conflict and error processing in healthy populations: Insights from transcranial electric stimulation studies. Front Integr Neurosci 2022;16:953928.

70. Gatti D, Rinaldi L, Cristea I, Vecchi T. Probing cerebellar involvement in cognition through a meta-analysis of TMS evidence. Sci Rep 2021;11:14777.

71. Gondard E, Teves L, Wang L et al. Deep Brain Stimulation Rescues Memory and Synaptic Activity in a Rat Model of Global Ischemia. J Neurosci 2019;39:2430-2440.

72. Griskova-Bulanova I, Sveistyte K, Bjekic J. Neuromodulation of Gamma-Range Auditory Steady-State Responses: A Scoping Review of Brain Stimulation Studies. Front Syst Neurosci 2020;14:41.

73. Guo F, Han X, Zhang J et al. Repetitive transcranial magnetic stimulation promotes neural stem cell proliferation via the regulation of MiR-25 in a rat model of focal cerebral ischemia. PLoS One 2014;9:e109267.

74. Guo F, Lou J, Han X, Deng Y, Huang X. Repetitive Transcranial Magnetic Stimulation Ameliorates Cognitive Impairment by Enhancing Neurogenesis and Suppressing Apoptosis in the Hippocampus in Rats with Ischemic Stroke. Front Physiol 2017;8:559.

75. Guo T, Li H, Lv Y et al. Pulsed Transcranial Ultrasound Stimulation Immediately After The Ischemic Brain Injury is Neuroprotective. IEEE Trans Biomed Eng 2015;62:2352-7.

76. Gutierrez-Fernandez M, Fuentes B, Rodriguez-Frutos B, Ramos-Cejudo J, Vallejo-Cremades MT, Diez-Tejedor E. Trophic factors and cell therapy to stimulate brain repair after ischaemic stroke. J Cell Mol Med 2012;16:2280-90.

77. Gutierrez-Mercado YK, Canedo-Dorantes L, Gomez-Pinedo U, Serrano-Luna G, Banuelos-Pineda J, Feria-Velasco A. Increased vascular permeability in the circumventricular organs of adult rat brain due to stimulation by extremely low frequency magnetic fields. Bioelectromagnetics 2013;34:145-55.

78. Haghani M, Keshavarz S, Nazari M, Rafati A. Electrophysiology of cerebral ischemia and reperfusion: First evidence for the role of synapse in ischemic tolerance. Synapse 2016;70:351-60.

79. Hanajima R, Ugawa Y. Triad TMS of the human motor cortex. Neurosci Res 2020;156:245-249.

80. Hartwigsen G. The neurophysiology of language: Insights from non-invasive brain stimulation in the healthy human brain. Brain Lang 2015;148:81-94.

81. Heidarzadegan AR, Zarifkar A, Sotoudeh N, Namavar MR, Zarifkar AH. Different paradigms of transcranial electrical stimulation improve motor function impairment and striatum tissue injuries in the collagenase-induced intracerebral hemorrhage rat model. BMC Neurosci 2022;23:6.

82. Hermann JK, Borseth A, Pucci FG et al. Changes in somatosensory evoked potentials elicited by lateral cerebellar nucleus deep brain stimulation in the naive rodent. Neurosci Lett 2022;786:136800.

83. Hill AT, Fitzgerald PB, Hoy KE. Effects of Anodal Transcranial Direct Current Stimulation on Working Memory: A Systematic Review and Meta-Analysis of Findings From Healthy and Neuropsychiatric Populations. Brain Stimul 2016;9:197-208.

84. Ho KA, Taylor JL, Chew T et al. The Effect of Transcranial Direct Current Stimulation (tDCS) Electrode Size and Current Intensity on Motor Cortical Excitability: Evidence From Single and Repeated Sessions. Brain Stimul 2016;9:1-7.

85. Hoebeke Y, Desmedt O, Ozcimen B, Heeren A. The impact of transcranial Direct Current stimulation on rumination: A systematic review of the sham-controlled studies in healthy and clinical samples. Compr Psychiatry 2021;106:152226.

86. Hong Y, Liu Q, Peng M et al. High-frequency repetitive transcranial magnetic stimulation improves functional recovery by inhibiting neurotoxic polarization of astrocytes in ischemic rats. J Neuroinflammation 2020;17:150.

87. Hong Y, Lyu J, Zhu L et al. High-frequency repetitive transcranial magnetic stimulation (rTMS) protects against ischemic stroke by inhibiting M1 microglia polarization through let-7b-5p/HMGA2/NF-kappaB signaling pathway. BMC Neurosci 2022;23:49.

88. Horvath JC, Forte JD, Carter O. Evidence that transcranial direct current stimulation (tDCS) generates little-to-no reliable neurophysiologic effect beyond MEP amplitude modulation in healthy human subjects: A systematic review. Neuropsychologia 2015;66:213-36.

89. Horvath JC, Forte JD, Carter O. Quantitative Review Finds No Evidence of Cognitive Effects in Healthy Populations From Single-session Transcranial Direct Current Stimulation (tDCS). Brain Stimul 2015;8:535-50.

90. Hu K, Chen Y, Guo F, Wang X. Effects of Transcranial Direct Current Stimulation on Upper Limb Muscle Strength and Endurance in Healthy Individuals: A Systematic Review and Meta-Analysis. Front Physiol 2022;13:834397.

91. Hu Y, Li R, Yang H, Luo H, Chen Z. Sirtuin 6 is essential for sodium sulfide-mediated cytoprotective effect in ischemia/reperfusion-stimulated brain endothelial cells. J Stroke Cerebrovasc Dis 2015;24:601-9.

92. Huang J, Zhao K, Zhao Z, Qu Y. Neuroprotection by Transcranial Direct Current Stimulation in Rodent Models of Focal Ischemic Stroke: A Meta-Analysis. Front Neurosci 2021;15:761971.

93. Huang LG, Li JP, Pang XM et al. MicroRNA-29c Correlates with Neuroprotection Induced by FNS by Targeting Both Birc2 and Bak1 in Rat Brain after Stroke. CNS Neurosci Ther 2015;21:496-503.

94. Hurley R, Machado L. Using transcranial direct current stimulation to improve verbal working memory: A detailed review of the methodology. J Clin Exp Neuropsychol 2018;40:790-804.

95. Ibanez J, Zicher B, Brown KE et al. Standard intensities of transcranial alternating current stimulation over the motor cortex do not entrain corticospinal inputs to motor neurons. J Physiol 2022.

96. Ishida A, Isa K, Umeda T et al. Causal Link between the Cortico-Rubral Pathway and Functional Recovery through Forced Impaired Limb Use in Rats with Stroke. J Neurosci 2016;36:455-67.

97. Jackson MP, Rahman A, Lafon B et al. Animal models of transcranial direct current stimulation: Methods and mechanisms. Clin Neurophysiol 2016;127:3425-3454.

98. Jang DK, Park SI, Han YM et al. Motor-evoked potential confirmation of functional improvement by transplanted bone marrow mesenchymal stem cell in the ischemic rat brain. J Biomed Biotechnol 2011;2011:238409.

99. Janssens SEW, Sack AT. Spontaneous Fluctuations in Oscillatory Brain State Cause Differences in Transcranial Magnetic Stimulation Effects Within and Between Individuals. Front Hum Neurosci 2021;15:802244.

100. Jefferies E. The neural basis of semantic cognition: converging evidence from neuropsychology, neuroimaging and TMS. Cortex 2013;49:611-25.

101. Jiang B, He D. Repetitive transcranial magnetic stimulation (rTMS) fails to increase serum brain-derived neurotrophic factor (BDNF). Neurophysiol Clin 2019;49:295-300.

102. Jiang T, Xu RX, Zhang AW et al. Effects of transcranial direct current stimulation on hemichannel pannexin-1 and neural plasticity in rat model of cerebral infarction. Neuroscience 2012;226:421-6.

103. Joyal M, Fecteau S. Transcranial Direct Current Stimulation Effects on Semantic Processing in Healthy Individuals. Brain Stimul 2016;9:682-691.

104. Kadono Y, Koguchi K, Okada KI et al. Repetitive transcranial magnetic stimulation restores altered functional connectivity of central poststroke pain model monkeys. Sci Rep 2021;11:6126.

105. Katsoulaki M, Kastrinis A, Tsekoura M. The Effects of Anodal Transcranial Direct Current Stimulation on Working Memory. Adv Exp Med Biol 2017;987:283-289.

106. Kaviannejad R, Karimian SM, Riahi E, Ashabi G. The neuroprotective effects of transcranial direct current stimulation on global cerebral ischemia and reperfusion via modulating apoptotic pathways. Brain Res Bull 2022;186:70-78.

107. Kaviannejad R, Karimian SM, Riahi E, Ashabi G. A Single Immediate Use of the Cathodal Transcranial Direct Current Stimulation Induces Neuroprotection of Hippocampal Region Against Global Cerebral Ischemia. J Stroke Cerebrovasc Dis 2022;31:106241.

108. Kaviannejad R, Karimian SM, Riahi E, Ashabi G. Using dual polarities of transcranial direct current stimulation in global cerebral ischemia and its following reperfusion period attenuates neuronal injury. Metab Brain Dis 2022;37:1503-1516.

109. Klaus J, Schutter D. Non-invasive brain stimulation to investigate language production in healthy speakers: A meta-analysis. Brain Cogn 2018;123:10-22.

110. Kloosterboer E, Funke K. Repetitive transcranial magnetic stimulation recovers cortical map plasticity induced by sensory deprivation due to deafferentiation. J Physiol 2019;597:4025-4051.

111. Kotilainen T, Lehto SM. [Methods of brain stimulation based on weak electric current--future tool for the clinician?]. Duodecim 2016;132:306-12.

112. Krause MR, Zanos TP, Csorba BA et al. Transcranial Direct Current Stimulation Facilitates Associative Learning and Alters Functional Connectivity in the Primate Brain. Curr Biol 2017;27:3086-3096 e3.

113. Kremer KL, Smith AE, Sandeman L, Inglis JM, Ridding MC, Koblar SA. Transcranial Magnetic Stimulation of Human Adult Stem Cells in the Mammalian Brain. Front Neural Circuits 2016;10:17.

114. Krieg SM, Lioumis P, Makela JP et al. Protocol for motor and language mapping by navigated TMS in patients and healthy volunteers; workshop report. Acta Neurochir (Wien) 2017;159:1187-1195.

115. Kumari N, Taylor D, Signal N. The Effect of Cerebellar Transcranial Direct Current Stimulation on Motor Learning: A Systematic Review of Randomized Controlled Trials. Front Hum Neurosci 2019;13:328.

116. Lage C, Wiles K, Shergill SS, Tracy DK. A systematic review of the effects of low-frequency repetitive transcranial magnetic stimulation on cognition. J Neural Transm (Vienna) 2016;123:1479-1490.

117. Laskov O, Klirova M. Effects of deep transcranial magnetic stimulation (dTMS) on cognition. Neurosci Lett 2021;755:135906.

118. Lavezzi GD, Sanz Galan S, Andersen H, Tomer D, Cacciamani L. The effects of tDCS on object perception: A systematic review and meta-analysis. Behav Brain Res 2022;430:113927.

119. Lee BK, Lee WJ, Jung YS. Chrysin Attenuates VCAM-1 Expression and Monocyte Adhesion in Lipopolysaccharide-Stimulated Brain Endothelial Cells by Preventing NF-kappaB Signaling. Int J Mol Sci 2017;18.

120. Lee JH, Jung BH, Yoo KY. Application time and persistence of transcranial direct current stimulation (tDCS) against neuronal death resulting from transient cerebral ischemia. Lab Anim Res 2022;38:12.

121. Lenz M, Eichler A, Vlachos A. Monitoring and Modulating Inflammation-Associated Alterations in Synaptic Plasticity: Role of Brain Stimulation and the Blood-Brain Interface. Biomolecules 2021;11.

122. Li H, Shang J, Zhang C, Lu R, Chen J, Zhou X. Repetitive Transcranial Magnetic Stimulation Alleviates Neurological Deficits After Cerebral Ischemia Through Interaction Between RACK1 and BDNF exon IV by the Phosphorylation-Dependent Factor MeCP2. Neurotherapeutics 2020;17:651-663.

123. Li LM, Uehara K, Hanakawa T. The contribution of interindividual factors to variability of response in transcranial direct current stimulation studies. Front Cell Neurosci 2015;9:181.

124. Li M, Peng J, Song Y, Liang H, Mei Y, Fang Y. Electro-acupuncture combined with transcranial magnetic stimulation improves learning and memory function of rats with cerebral infarction by inhibiting neuron cell apoptosis. J Huazhong Univ Sci Technolog Med Sci 2012;32:746-749.

125. Li X, Yao J, Zhang W, Chen S, Peng W. Effects of transcranial direct current stimulation on experimental pain perception: A systematic review and meta-analysis. Clin Neurophysiol 2021;132:2163-2175.

126. Liang H, Xu C, Hu S et al. Repetitive Transcranial Magnetic Stimulation Improves Neuropathy and Oxidative Stress Levels in Rats with Experimental Cerebral Infarction through the Nrf2 Signaling Pathway. Evid Based Complement Alternat Med 2021;2021:3908677.

127. Lipp J, Draganova R, Batsikadze G, Ernst TM, Uengoer M, Timmann D. Prefrontal but not cerebellar tDCS attenuates renewal of extinguished conditioned eyeblink responses. Neurobiol Learn Mem 2020;170:107137.

128. Liu L, Du J, Zheng T et al. Protective effect of low-intensity transcranial ultrasound stimulation after differing delay following an acute ischemic stroke. Brain Res Bull 2019;146:22-27.

129. Liu YH, Chan SJ, Pan HC et al. Integrated treatment modality of cathodal-transcranial direct current stimulation with peripheral sensory stimulation affords neuroprotection in a rat stroke model. Neurophotonics 2017;4:045002.

130. Ljubisavljevic MR, Javid A, Oommen J et al. The Effects of Different Repetitive Transcranial Magnetic Stimulation (rTMS) Protocols on Cortical Gene Expression in a Rat Model of Cerebral Ischemic-Reperfusion Injury. PLoS One 2015;10:e0139892.

131. Longo V, Barbati SA, Re A et al. Transcranial Direct Current Stimulation Enhances Neuroplasticity and Accelerates Motor Recovery in a Stroke Mouse Model. Stroke 2022;53:1746-1758.

132. Luber B, Lisanby SH. Enhancement of human cognitive performance using transcranial magnetic stimulation (TMS). Neuroimage 2014;85 Pt 3:961-70.

133. Luedtke K, Rushton A, Wright C, Geiss B, Juergens TP, May A. Transcranial direct current stimulation for the reduction of clinical and experimentally induced pain: a systematic review and meta-analysis. Clin J Pain 2012;28:452-61.

134. Luo J, Feng Y, Li M, Yin M, Qin F, Hu X. Repetitive Transcranial Magnetic Stimulation Improves Neurological Function and Promotes the Anti-inflammatory Polarization of Microglia in Ischemic Rats. Front Cell Neurosci 2022;16:878345.

135. Luo J, Zheng H, Zhang L et al. High-Frequency Repetitive Transcranial Magnetic Stimulation (rTMS) Improves Functional Recovery by Enhancing Neurogenesis and Activating BDNF/TrkB Signaling in Ischemic Rats. Int J Mol Sci 2017;18.

136. Luo YP, Zhang L, Wu XY et al. Cerebral blood microcirculation measurement in APP/PS1 double transgenic mice at the preclinical stage of Alzheimer's disease: preliminary data on the early intervention of anodal transcranial direct current stimulation(). Annu Int Conf IEEE Eng Med Biol Soc 2020;2020:3557-3560.

137. Ma X, Cheng O, Jiang Q, Yang J, Xiao H, Qiu H. Activation of ephrinb1/EPHB2/MAP-2/NMDAR Mediates Hippocampal Neurogenesis Promoted by Transcranial Direct Current Stimulation in Cerebral-Ischemic Mice. Neuromolecular Med 2021;23:521-530.

138. Machado AG, Cooperrider J, Furmaga HT et al. Chronic 30-Hz deep cerebellar stimulation coupled with training enhances post-ischemia motor recovery and peri-infarct synaptophysin expression in rodents. Neurosurgery 2013;73:344-53; discussion 353.

139. Maeda Y, Otsuka T, Mitsuhara T, Okazaki T, Yuge L, Takeda M. A novel bone-thinning technique for transcranial stimulation motor-evoked potentials in rats. Sci Rep 2021;11:12496.

140. Malinova V, Bleuel K, Stadelmann C et al. The impact of transcranial direct current stimulation on cerebral vasospasm in a rat model of subarachnoid hemorrhage. J Cereb Blood Flow Metab 2021;41:2000-2009.

141. Malkani RG, Zee PC. Brain Stimulation for Improving Sleep and Memory. Sleep Med Clin 2020;15:101-115.

142. Mancuso LE, Ilieva IP, Hamilton RH, Farah MJ. Does Transcranial Direct Current Stimulation Improve Healthy Working Memory?: A Meta-analytic Review. J Cogn Neurosci 2016;28:1063-89.

143. Manto M, Argyropoulos GPD, Bocci T et al. Consensus Paper: Novel Directions and Next Steps of Non-invasive Brain Stimulation of the Cerebellum in Health and Disease. Cerebellum 2022;21:1092-1122.

144. Martin-Trias P, Bragulat V, Pena-Gomez C et al. Translational Challenge Models in Support of Efficacy Studies: Neurobehavioral and Cognitive Changes Induced by Transcranial Magnetic Stimulation in Healthy Volunteers. CNS Neurol Disord Drug Targets 2016;15:802-15.

145. Massetti T, Crocetta TB, Silva TDD et al. Application and outcomes of therapy combining transcranial direct current stimulation and virtual reality: a systematic review. Disabil Rehabil Assist Technol 2017;12:551-559.

146. Matsumoto H, Ugawa Y. Adverse events of tDCS and tACS: A review. Clin Neurophysiol Pract 2017;2:19-25.

147. Medina J, Cason S. No evidential value in samples of transcranial direct current stimulation (tDCS) studies of cognition and working memory in healthy populations. Cortex 2017;94:131-141.

148. Meng Q, Cherry M, Refai A et al. Development of Focused Transcranial Magnetic Stimulation for Rodents by Copper-Array Shields. IEEE Trans Magn 2018;54.

149. Micera S, Caleo M, Chisari C, Hummel FC, Pedrocchi A. Advanced Neurotechnologies for the Restoration of Motor Function. Neuron 2020;105:604-620.

150. Mielacher C, Scheele D, Hurlemann R. [Experimental and therapeutic neuromodulation of emotion and social cognition with non-invasive brain stimulation]. Nervenarzt 2015;86:1500-7.

151. Miterko LN, Baker KB, Beckinghausen J et al. Consensus Paper: Experimental Neurostimulation of the Cerebellum. Cerebellum 2019;18:1064-1097.

152. Mondino M, Thiffault F, Fecteau S. Does non-invasive brain stimulation applied over the dorsolateral prefrontal cortex non-specifically influence mood and emotional processing in healthy individuals? Front Cell Neurosci 2015;9:399.

153. Morimoto T, Yasuhara T, Kameda M et al. Striatal stimulation nurtures endogenous neurogenesis and angiogenesis in chronic-phase ischemic stroke rats. Cell Transplant 2011;20:1049-64.

154. Morya E, Monte-Silva K, Bikson M et al. Beyond the target area: an integrative view of tDCS-induced motor cortex modulation in patients and athletes. J Neuroeng Rehabil 2019;16:141.

155. Moscatelli F, Messina A, Valenzano A et al. Transcranial Magnetic Stimulation as a Tool to Investigate Motor Cortex Excitability in Sport. Brain Sci 2021;11.

156. Neige C, Masse-Alarie H, Mercier C. Stimulating the Healthy Brain to Investigate Neural Correlates of Motor Preparation: A Systematic Review. Neural Plast 2018;2018:5846096.

157. Nielsen RK, Jensen W. Low-Frequency Intracortical Electrical Stimulation Decreases Sensorimotor Cortex Hyperexcitability in the Acute Phase of Ischemic Stroke. IEEE Trans Neural Syst Rehabil Eng 2017;25:1287-1296.

158. Nikolin S, Loo CK, Bai S, Dokos S, Martin DM. Focalised stimulation using high definition transcranial direct current stimulation (HD-tDCS) to investigate declarative verbal learning and memory functioning. Neuroimage 2015;117:11-9.

159. Nitsche MA, Muller-Dahlhaus F, Paulus W, Ziemann U. The pharmacology of neuroplasticity induced by non-invasive brain stimulation: building models for the clinical use of CNS active drugs. J Physiol 2012;590:4641-62.

160. Notturno F, Pace M, Zappasodi F, Cam E, Bassetti CL, Uncini A. Neuroprotective effect of cathodal transcranial direct current stimulation in a rat stroke model. J Neurol Sci 2014;342:146-51.

161. Oldrati V, Schutter D. Targeting the Human Cerebellum with Transcranial Direct Current Stimulation to Modulate Behavior: a Meta-Analysis. Cerebellum 2018;17:228-236.

162. Pang N, Meng W, Zhong Y et al. Ultrasound Deep Brain Stimulation Modulates Body Temperature in Mice. IEEE Trans Neural Syst Rehabil Eng 2022;30:1851-1857.

163. Park HJ, Furmaga H, Cooperrider J, Gale JT, Baker KB, Machado AG. Modulation of Cortical Motor Evoked Potential After Stroke During Electrical Stimulation of the Lateral Cerebellar Nucleus. Brain Stimul 2015;8:1043-8.

164. Park HK, Song MK, Kim WI, Han JY. Regulation of gene expression after combined scalp acupuncture and transcranial magnetic stimulation in middle cerebral artery occlusion mice. Restor Neurol Neurosci 2020;38:253-263.

165. Pastore-Wapp M, Nyffeler T, Nef T, Bohlhalter S, Vanbellingen T. Non-invasive brain stimulation in limb praxis and apraxia: A scoping review in healthy subjects and patients with stroke. Cortex 2021;138:152-164.

166. Peng JJ, Sha R, Li MX et al. Repetitive transcranial magnetic stimulation promotes functional recovery and differentiation of human neural stem cells in rats after ischemic stroke. Exp Neurol 2019;313:1-9.

167. Pikhovych A, Stolberg NP, Jessica Flitsch L et al. Transcranial Direct Current Stimulation Modulates Neurogenesis and Microglia Activation in the Mouse Brain. Stem Cells Int 2016;2016:2715196.

168. Pikhovych A, Walter HL, Mahabir E et al. Transcranial direct current stimulation in the male mouse to promote recovery after stroke. Lab Anim 2016;50:212-6.

169. Pimentel VC, Pinheiro FV, De Bona KS et al. Hypoxic-ischemic brain injury stimulates inflammatory response and enzymatic activities in the hippocampus of neonatal rats. Brain Res 2011;1388:134-40.

170. Pino-Esteban A, Megia-Garcia A, Martin-Caro Alvarez D et al. Can Transcranial Direct Current Stimulation Enhance Functionality in Older Adults? A Systematic Review. J Clin Med 2021;10.

171. Pinter MM, Brainin M. Role of repetitive transcranial magnetic stimulation in stroke rehabilitation. Front Neurol Neurosci 2013;32:112-21.

172. Pixa NH, Pollok B. Effects of tDCS on Bimanual Motor Skills: A Brief Review. Front Behav Neurosci 2018;12:63.

173. Plow EB, Cunningham DA, Varnerin N, Machado A. Rethinking stimulation of the brain in stroke rehabilitation: why higher motor areas might be better alternatives for patients with greater impairments. Neuroscientist 2015;21:225-40.

174. Plow EB, Sankarasubramanian V, Cunningham DA et al. Models to Tailor Brain Stimulation Therapies in Stroke. Neural Plast 2016;2016:4071620.

175. Pollok B, Schmitz-Justen C, Krause V. Cathodal Transcranial Direct Current Stimulation (tDCS) Applied to the Left Premotor Cortex Interferes with Explicit Reproduction of a Motor Sequence. Brain Sci 2021;11.

176. Ramaraju S, Roula MA, McCarthy PW. Transcranial direct current stimulation and working memory: Comparison of effect on learning shapes and English letters. PLoS One 2020;15:e0222688.

177. Reis J, Fritsch B. Modulation of motor performance and motor learning by transcranial direct current stimulation. Curr Opin Neurol 2011;24:590-6.

178. Romanella SM, Roe D, Paciorek R et al. Sleep, Noninvasive Brain Stimulation, and the Aging Brain: Challenges and Opportunities. Ageing Res Rev 2020;61:101067.

179. Rossi S, Antal A, Bestmann S et al. Safety and recommendations for TMS use in healthy subjects and patient populations, with updates on training, ethical and regulatory issues: Expert Guidelines. Clin Neurophysiol 2021;132:269-306.

180. Rossi S, Santarnecchi E, Feurra M. Noninvasive brain stimulation and brain oscillations. Handb Clin Neurol 2022;184:239-247.

181. Rostami M, Zomorrodi R, Rostami R, Hosseinzadeh GA. Impact of methodological variability on EEG responses evoked by transcranial magnetic stimulation: A meta-analysis. Clin Neurophysiol 2022;142:154-180.

182. Rothwell J. Transcranial brain stimulation: Past and future. Brain Neurosci Adv 2018;2:2398212818818070.

183. Roux C, Kaeser M, Savidan J, Fregosi M, Rouiller EM, Schmidlin E. Assessment of the effect of continuous theta burst stimulation of the motor cortex on manual dexterity in non-human primates in a direct comparison with invasive intracortical pharmacological inactivation. Eur J Neurosci 2019;50:3599-3613.

184. Roy LB, Sparing R, Fink GR, Hesse MD. Modulation of attention functions by anodal tDCS on right PPC. Neuropsychologia 2015;74:96-107.

185. Rueger MA, Keuters MH, Walberer M et al. Multi-session transcranial direct current stimulation (tDCS) elicits inflammatory and regenerative processes in the rat brain. PLoS One 2012;7:e43776.

186. Sanchez-Kuhn A, Perez-Fernandez C, Canovas R, Flores P, Sanchez-Santed F. Transcranial direct current stimulation as a motor neurorehabilitation tool: an empirical review. Biomed Eng Online 2017;16:76.

187. Sasaki R, Kojima S, Onishi H. Do Brain-Derived Neurotrophic Factor Genetic Polymorphisms Modulate the Efficacy of Motor Cortex Plasticity Induced by Non-invasive Brain Stimulation? A Systematic Review. Front Hum Neurosci 2021;15:742373.

188. Schestatsky P, Simis M, Freeman R, Pascual-Leone A, Fregni F. Non-invasive brain stimulation and the autonomic nervous system. Clin Neurophysiol 2013;124:1716-28.

189. Schjetnan AG, Gidyk DC, Metz GA, Luczak A. Anodal transcranial direct current stimulation with monopolar pulses improves limb use after stroke by enhancing inter-hemispheric coherence. Acta Neurobiol Exp (Wars) 2019;79:290-301.

190. Schulz R, Gerloff C, Hummel FC. Non-invasive brain stimulation in neurological diseases. Neuropharmacology 2013;64:579-87.

191. Shah AM, Ishizaka S, Cheng MY et al. Optogenetic neuronal stimulation of the lateral cerebellar nucleus promotes persistent functional recovery after stroke. Sci Rep 2017;7:46612.

192. Siegert A, Diedrich L, Antal A. New Methods, Old Brains-A Systematic Review on the Effects of tDCS on the Cognition of Elderly People. Front Hum Neurosci 2021;15:730134.

193. Siew-Pin Leuk J, Yow KE, Zi-Xin Tan C et al. A meta-analytical review of transcranial direct current stimulation parameters on upper limb motor learning in healthy older adults and people with Parkinson's disease. Rev Neurosci 2023;34:325-348.

194. Sims SK, Rizzo A, Howard K, Farrand A, Boger H, Adkins DL. Comparative Enhancement of Motor Function and BDNF Expression Following Different Brain Stimulation Approaches in an Animal Model of Ischemic Stroke. Neurorehabil Neural Repair 2020;34:925-935.

195. Skelding KA, Arellano JM, Powis DA, Rostas JA. Excitotoxic stimulation of brain microslices as an in vitro model of stroke. J Vis Exp 2014:e51291.

196. Sollmann N, Ille S, Negwer C et al. Cortical time course of object naming investigated by repetitive navigated transcranial magnetic stimulation. Brain Imaging Behav 2017;11:1192-1206.

197. Song S, Yu L, Hasan MN et al. Elevated microglial oxidative phosphorylation and phagocytosis stimulate post-stroke brain remodeling and cognitive function recovery in mice. Commun Biol 2022;5:35.

198. Talar K, Vetrovsky T, van Haren M et al. The effects of aerobic exercise and transcranial direct current stimulation on cognitive function in older adults with and without cognitive impairment: A systematic review and meta-analysis. Ageing Res Rev 2022;81:101738.

199. Tang A, Thickbroom G, Rodger J. Repetitive Transcranial Magnetic Stimulation of the Brain: Mechanisms from Animal and Experimental Models. Neuroscientist 2017;23:82-94.

200. Tang C, Xue HL, Bai CL, Fu R. Regulation of adhesion molecules expression in TNF-alpha-stimulated brain microvascular endothelial cells by tanshinone IIA: involvement of NF-kappaB and ROS generation. Phytother Res 2011;25:376-80.

201. Tatti E, Rossi S, Innocenti I, Rossi A, Santarnecchi E. Non-invasive brain stimulation of the aging brain: State of the art and future perspectives. Ageing Res Rev 2016;29:66-89.

202. Teti Mayer J, Chopard G, Nicolier M et al. Can transcranial direct current stimulation (tDCS) improve impulsivity in healthy and psychiatric adult populations? A systematic review. Prog Neuropsychopharmacol Biol Psychiatry 2020;98:109814.

203. To WT, Hart J, De Ridder D, Vanneste S. Considering the influence of stimulation parameters on the effect of conventional and high-definition transcranial direct current stimulation. Expert Rev Med Devices 2016;13:391-404.

204. Tomlinson SP, Davis NJ, Bracewell RM. Brain stimulation studies of non-motor cerebellar function: a systematic review. Neurosci Biobehav Rev 2013;37:766-89.

205. Tremblay S, Austin D, Hannah R, Rothwell JC. Non-invasive brain stimulation as a tool to study cerebellar-M1 interactions in humans. Cerebellum Ataxias 2016;3:19.

206. Tremblay S, Beaule V, Proulx S et al. The use of magnetic resonance spectroscopy as a tool for the measurement of bi-hemispheric transcranial electric stimulation effects on primary motor cortex metabolism. J Vis Exp 2014:e51631.

207. Trevathan JK, Yousefi A, Park HO et al. Computational Modeling of Neurotransmitter Release Evoked by Electrical Stimulation: Nonlinear Approaches to Predicting Stimulation-Evoked Dopamine Release. ACS Chem Neurosci 2017;8:394-410.

208. Tu Y, Cao J, Guler S et al. Perturbing fMRI brain dynamics using transcranial direct current stimulation. Neuroimage 2021;237:118100.

209. Turner DA, Degan S, Galeffi F, Schmidt S, Peterchev AV. Rapid, Dose-Dependent Enhancement of Cerebral Blood Flow by transcranial AC Stimulation in Mouse. Brain Stimul 2021;14:80-87.

210. Ueda Y, Masuda T, Ishida A et al. Enhanced electrical responsiveness in the cerebral cortex with oral melatonin administration after a small hemorrhage near the internal capsule in rats. J Neurosci Res 2014;92:1499-508.

211. Uzair M, Arshad M, Abualait T et al. Neurobiology of amphetamine use in stroke recovery combined with rehabilitative training and brain stimulation. CNS Neurol Disord Drug Targets 2022.

212. Valero-Cabre A, Pascual-Leone A, Coubard OA. [Transcranial magnetic stimulation (TMS) in basic and clinical neuroscience research]. Rev Neurol (Paris) 2011;167:291-316.

213. van Dun K, Bodranghien FC, Marien P, Manto MU. tDCS of the Cerebellum: Where Do We Stand in 2016? Technical Issues and Critical Review of the Literature. Front Hum Neurosci 2016;10:199.

214. Varnerin N, Mirando D, Potter-Baker KA et al. Assessment of Vascular Stent Heating with Repetitive Transcranial Magnetic Stimulation. J Stroke Cerebrovasc Dis 2017;26:1121-1127.

215. Veniero D, Vossen A, Gross J, Thut G. Lasting EEG/MEG Aftereffects of Rhythmic Transcranial Brain Stimulation: Level of Control Over Oscillatory Network Activity. Front Cell Neurosci 2015;9:477.

216. Volz MS, Volz TS, Brunoni AR, de Oliveira JP, Fregni F. Analgesic effects of noninvasive brain stimulation in rodent animal models: a systematic review of translational findings. Neuromodulation 2012;15:283-95.

217. Vonloh M, Chen R, Kluger B. Safety of transcranial magnetic stimulation in Parkinson's disease: a review of the literature. Parkinsonism Relat Disord 2013;19:573-85.

218. Vosskuhl J, Struber D, Herrmann CS. Non-invasive Brain Stimulation: A Paradigm Shift in Understanding Brain Oscillations. Front Hum Neurosci 2018;12:211.

219. Wachter D, Wrede A, Schulz-Schaeffer W et al. Transcranial direct current stimulation induces polarity-specific changes of cortical blood perfusion in the rat. Exp Neurol 2011;227:322-7.

220. Walter HL, Pikhovych A, Endepols H et al. Transcranial-Direct-Current-Stimulation Accelerates Motor Recovery After Cortical Infarction in Mice: The Interplay of Structural Cellular Responses and Functional Recovery. Neurorehabil Neural Repair 2022;36:701-714.

221. Wang J, Dong WW, Zhang WH, Zheng J, Wang X. Electrical stimulation of cerebellar fastigial nucleus: mechanism of neuroprotection and prospects for clinical application against cerebral ischemia. CNS Neurosci Ther 2014;20:710-6.

222. Wang J, Li G, Deng L et al. Transcranial Focused Ultrasound Stimulation Improves Neurorehabilitation after Middle Cerebral Artery Occlusion in Mice. Aging Dis 2021;12:50-60.

223. Wang M, Guo J, Dong LN, Wang JP. Cerebellar Fastigial Nucleus Stimulation in a Chronic Unpredictable Mild Stress Rat Model Reduces Post-Stroke Depression by Suppressing Brain Inflammation via the microRNA-29c/TNFRSF1A Signaling Pathway. Med Sci Monit 2019;25:5594-5605.

224. Wang P, Zhang J, Yu J, Smith C, Feng W. Brain Modulatory Effects by Low-Intensity Transcranial Ultrasound Stimulation (TUS): A Systematic Review on Both Animal and Human Studies. Front Neurosci 2019;13:696.

225. Wang WJ, Zhong YB, Zhao JJ et al. Transcranial pulse current stimulation improves the locomotor function in a rat model of stroke. Neural Regen Res 2021;16:1229-1234.

226. Wang Y, Cooke MJ, Sachewsky N, Morshead CM, Shoichet MS. Bioengineered sequential growth factor delivery stimulates brain tissue regeneration after stroke. J Control Release 2013;172:1-11.

227. Wang Y, Wang J, Zhang QF et al. Neural Mechanism Underlying Task-Specific Enhancement of Motor Learning by Concurrent Transcranial Direct Current Stimulation. Neurosci Bull 2023;39:69-82.

228. Wathen CA, Frizon LA, Maiti TK, Baker KB, Machado AG. Deep brain stimulation of the cerebellum for poststroke motor rehabilitation: from laboratory to clinical trial. Neurosurg Focus 2018;45:E13.

229. Weissman-Fogel I, Granovsky Y. The "virtual lesion" approach to transcranial magnetic stimulation: studying the brain-behavioral relationships in experimental pain. Pain Rep 2019;4:e760.

230. Wertheim J, Colzato LS, Nitsche MA, Ragni M. Enhancing spatial reasoning by anodal transcranial direct current stimulation over the right posterior parietal cortex. Exp Brain Res 2020;238:181-192.

231. Westwood SJ, Romani C. Transcranial direct current stimulation (tDCS) modulation of picture naming and word reading: A meta-analysis of single session tDCS applied to healthy participants. Neuropsychologia 2017;104:234-249.

232. Winstein CJ, Kay DB. Translating the science into practice: shaping rehabilitation practice to enhance recovery after brain damage. Prog Brain Res 2015;218:331-60.

233. Wischnewski M, Schutter D, Nitsche MA. Effects of beta-tACS on corticospinal excitability: A meta-analysis. Brain Stimul 2019;12:1381-1389.

234. Worsching J, Padberg F, Ertl-Wagner B, Kumpf U, Kirsch B, Keeser D. Imaging transcranial direct current stimulation (tDCS) of the prefrontal cortex-correlation or causality in stimulation-mediated effects? Neurosci Biobehav Rev 2016;69:333-56.

235. Wu S, Zheng T, Du J et al. Neuroprotective effect of low-intensity transcranial ultrasound stimulation in endothelin-1-induced middle cerebral artery occlusion in rats. Brain Res Bull 2020;161:127-135.

236. Wu X, Wang C, Wang J, Zhu M, Yao Y, Liu J. Hypoxia preconditioning protects neuronal cells against traumatic brain injury through stimulation of glucose transport mediated by HIF-1alpha/GLUTs signaling pathway in rat. Neurosurg Rev 2021;44:411-422.

237. Wu Z, Sun F, Li Z et al. Electrical stimulation of the lateral cerebellar nucleus promotes neurogenesis in rats after motor cortical ischemia. Sci Rep 2020;10:16563.

238. Xing Y, Zhang Y, Li C et al. Repetitive Transcranial Magnetic Stimulation of the Brain After Ischemic Stroke: Mechanisms from Animal Models. Cell Mol Neurobiol 2023;43:1487-1497.

239. Yamada N, Kakuda W, Kondo T, Mitani S, Shimizu M, Abo M. Local muscle injection of botulinum toxin type a synergistically improves the beneficial effects of repetitive transcranial magnetic stimulation and intensive occupational therapy in post-stroke patients with spastic upper limb hemiparesis. Eur Neurol 2014;72:290-8.

240. Yang J, Liang R, Wang L, Zheng C, Xiao X, Ming D. Repetitive Transcranial Magnetic Stimulation (rTMS) Improves the Gait Disorders of Rats Under Simulated Microgravity Conditions Associated With the Regulation of Motor Cortex. Front Physiol 2021;12:587515.

241. Yang Y, Yang LY, Orban L et al. Non-invasive vagus nerve stimulation reduces blood-brain barrier disruption in a rat model of ischemic stroke. Brain Stimul 2018;11:689-698.

242. Yeh N, Rose NS. How Can Transcranial Magnetic Stimulation Be Used to Modulate Episodic Memory?: A Systematic Review and Meta-Analysis. Front Psychol 2019;10:993.

243. Yoon KJ, Lee YT, Han TR. Mechanism of functional recovery after repetitive transcranial magnetic stimulation (rTMS) in the subacute cerebral ischemic rat model: neural plasticity or anti-apoptosis? Exp Brain Res 2011;214:549-56.

244. Yoon KJ, Oh BM, Kim DY. Functional improvement and neuroplastic effects of anodal transcranial direct current stimulation (tDCS) delivered 1 day vs. 1 week after cerebral ischemia in rats. Brain Res 2012;1452:61-72.

245. Yu C, Xiao S, Wang B et al. Is Anodal Transcranial Direct Current Stimulation an Effective Ergogenic Technology in Lower Extremity Sensorimotor Control for Healthy Population? A Narrative Review. Brain Sci 2022;12.

246. Zebhauser PT, Vernet M, Unterburger E, Brem AK. Visuospatial Neglect - a Theory-Informed Overview of Current and Emerging Strategies and a Systematic Review on the Therapeutic Use of Non-invasive Brain Stimulation. Neuropsychol Rev 2019;29:397-420.

247. Zeng Y, Acord M, Kaovasia TP et al. A Miniature Laser Speckle Contrast Imager for Monitoring of the Neuro-Modulatory Effect of Transcranial Focused Ultrasound Stimulation. Proc 2021 Des Med Devices Conf DMD2021 (2021) 2021;2021.

248. Zhang K, Guo L, Zhang J et al. tDCS Accelerates the Rehabilitation of MCAO-Induced Motor Function Deficits via Neurogenesis Modulated by the Notch1 Signaling Pathway. Neurorehabil Neural Repair 2020;34:640-651.

249. Zhang KY, Rui G, Zhang JP et al. Cathodal tDCS exerts neuroprotective effect in rat brain after acute ischemic stroke. BMC Neurosci 2020;21:21.

250. Zhang L, Li S, Chen L et al. Cerebellar fastigial nucleus electrical stimulatin protects against cerebral ischemic damage by upregulating telomerase activity. Restor Neurol Neurosci 2019;37:131-141.

251. Zhang XQ, Li L, Huo JT, Cheng M, Li LH. Effects of repetitive transcranial magnetic stimulation on cognitive function and cholinergic activity in the rat hippocampus after vascular dementia. Neural Regen Res 2018;13:1384-1389.

252. Ziemann U, Reis J, Schwenkreis P et al. TMS and drugs revisited 2014. Clin Neurophysiol 2015;126:1847-68.

253. Zong X, Dong Y, Li Y et al. Beneficial Effects of Theta-Burst Transcranial Magnetic Stimulation on Stroke Injury via Improving Neuronal Microenvironment and Mitochondrial Integrity. Transl Stroke Res 2020;11:450-467.

254. Zong X, Gu J, Geng D, Gao D. Repetitive transcranial magnetic stimulation (rTMS) for multiple neurological conditions in rodent animal models: A systematic review. Neurochem Int 2022;157:105356.

255. Zong X, Li Y, Liu C et al. Theta-burst transcranial magnetic stimulation promotes stroke recovery by vascular protection and neovascularization. Theranostics 2020;10:12090-12110.

**Appendix 1g. Reference list for clinical trials (stroke patients) literature review**

1. Abo M. [Repetitive transcranial magnetic stimulation rehabilitation]. Rinsho Shinkeigaku 2013;23:1264-6.

2. Abo M, Kakuda W, Momosaki R et al. Randomized, multicenter, comparative study of NEURO versus CIMT in poststroke patients with upper limb hemiparesis: the NEURO-VERIFY Study. Int J Stroke 2014;9:607-12.

3. Abraha B, Chaves AR, Kelly LP et al. A Bout of High Intensity Interval Training Lengthened Nerve Conduction Latency to the Non-exercised Affected Limb in Chronic Stroke. Front Physiol 2018;9:827.

4. Ackerley SJ, Byblow WD, Barber PA, MacDonald H, McIntyre-Robinson A, Stinear CM. Primed Physical Therapy Enhances Recovery of Upper Limb Function in Chronic Stroke Patients. Neurorehabil Neural Repair 2016;30:339-48.

5. Ackerley SJ, Stinear CM, Barber PA, Byblow WD. Priming sensorimotor cortex to enhance task-specific training after subcortical stroke. Clin Neurophysiol 2014;125:1451-8.

6. Ahmadi M, Laumeier I, Ihl T et al. A support programme for secondary prevention in patients with transient ischaemic attack and minor stroke (INSPiRE-TMS): an open-label, randomised controlled trial. Lancet Neurol 2020;19:49-60.

7. Ahn YH, Sohn HJ, Park JS et al. Effect of bihemispheric anodal transcranial direct current stimulation for dysphagia in chronic stroke patients: A randomized clinical trial. J Rehabil Med 2017;49:30-35.

8. Alber R, Moser H, Gall C, Sabel BA. Combined Transcranial Direct Current Stimulation and Vision Restoration Training in Subacute Stroke Rehabilitation: A Pilot Study. PM R 2017;9:787-794.

9. Alisar DC, Ozen S, Sozay S. Effects of Bihemispheric Transcranial Direct Current Stimulation on Upper Extremity Function in Stroke Patients: A randomized Double-Blind Sham-Controlled Study. J Stroke Cerebrovasc Dis 2020;29:104454.

10. Allman C, Amadi U, Winkler AM et al. Ipsilesional anodal tDCS enhances the functional benefits of rehabilitation in patients after stroke. Sci Transl Med 2016;8:330re1.

11. Andrade SM, Batista LM, Nogueira LL et al. Constraint-Induced Movement Therapy Combined with Transcranial Direct Current Stimulation over Premotor Cortex Improves Motor Function in Severe Stroke: A Pilot Randomized Controlled Trial. Rehabil Res Pract 2017;2017:6842549.

12. Andrade SM, Fernandez-Calvo B, Boggio PS et al. Neurostimulation for cognitive rehabilitation in stroke (NeuroCog): study protocol for a randomized controlled trial. Trials 2015;16:435.

13. Andrade SM, Ferreira JJA, Rufino TS et al. Effects of different montages of transcranial direct current stimulation on the risk of falls and lower limb function after stroke. Neurol Res 2017;39:1037-1043.

14. Andrade SM, Santos NA, Fernandez-Calvo B et al. Stroke Treatment Associated with Rehabilitation Therapy and Transcranial DC Stimulation (START-tDCS): a study protocol for a randomized controlled trial. Trials 2016;17:56.

15. Andreoli ML, Souza JT, Ribeiro PW et al. What are the barriers to participation in a neuromodulation pilot trial for aphasia after stroke? Codas 2021;33:e20200019.

16. Andressa de Souza J, Ferrari Correa JC, Marduy A et al. To Combine or Not to Combine Physical Therapy With tDCS for Stroke With Shoulder Pain? Analysis From a Combination Randomized Clinical Trial for Rehabilitation of Painful Shoulder in Stroke. Front Pain Res (Lausanne) 2021;2:696547.

17. Aneksan B, Sawatdipan M, Bovonsunthonchai S et al. Five-Session Dual-Transcranial Direct Current Stimulation With Task-Specific Training Does Not Improve Gait and Lower Limb Performance Over Training Alone in Subacute Stroke: A Pilot Randomized Controlled Trial. Neuromodulation 2022;25:558-568.

18. Ang KK, Guan C, Phua KS et al. Transcranial direct current stimulation and EEG-based motor imagery BCI for upper limb stroke rehabilitation. Annu Int Conf IEEE Eng Med Biol Soc 2012;2012:4128-31.

19. Ang KK, Guan C, Phua KS et al. Facilitating effects of transcranial direct current stimulation on motor imagery brain-computer interface with robotic feedback for stroke rehabilitation. Arch Phys Med Rehabil 2015;96:S79-87.

20. Ant JM, Niessen E, Achilles EIS et al. Anodal tDCS over left parietal cortex expedites recovery from stroke-induced apraxic imitation deficits: a pilot study. Neurol Res Pract 2019;1:38.

21. Arnao V, Riolo M, Carduccio F et al. Effects of transcranial random noise stimulation combined with Graded Repetitive Arm Supplementary Program (GRASP) on motor rehabilitation of the upper limb in sub-acute ischemic stroke patients: a randomized pilot study. J Neural Transm (Vienna) 2019;126:1701-1706.

22. Ashaie SA, Engel S, Cherney LR. Timing of transcranial direct current stimulation (tDCS) combined with speech and language therapy (SLT) for aphasia: study protocol for a randomized controlled trial. Trials 2022;23:668.

23. Askin A, Tosun A, Demirdal US. Effects of low-frequency repetitive transcranial magnetic stimulation on upper extremity motor recovery and functional outcomes in chronic stroke patients: A randomized controlled trial. Somatosens Mot Res 2017;34:102-107.

24. Au-Yeung SS, Wang J, Chen Y, Chua E. Transcranial direct current stimulation to primary motor area improves hand dexterity and selective attention in chronic stroke. Am J Phys Med Rehabil 2014;93:1057-64.

25. Avenanti A, Coccia M, Ladavas E, Provinciali L, Ceravolo MG. Low-frequency rTMS promotes use-dependent motor plasticity in chronic stroke: a randomized trial. Neurology 2012;78:256-64.

26. Bai G, Jiang L, Huan S et al. Study on Low-Frequency Repetitive Transcranial Magnetic Stimulation Improves Speech Function and Mechanism in Patients With Non-fluent Aphasia After Stroke. Front Aging Neurosci 2022;14:883542.

27. Bai G, Jiang L, Ma W et al. Effect of Low-Frequency rTMS and Intensive Speech Therapy Treatment on Patients With Nonfluent Aphasia After Stroke. Neurologist 2020;26:6-9.

28. Bakker CD, Massa M, Daffertshofer A et al. The addition of the MEP amplitude of finger extension muscles to clinical predictors of hand function after stroke: A prospective cohort study. Restor Neurol Neurosci 2019;37:445-456.

29. Ballester BR, Nirme J, Camacho I et al. Domiciliary VR-Based Therapy for Functional Recovery and Cortical Reorganization: Randomized Controlled Trial in Participants at the Chronic Stage Post Stroke. JMIR Serious Games 2017;5:e15.

30. Baltar A, Piscitelli D, Marques D, Shirahige L, Monte-Silva K. Baseline Motor Impairment Predicts Transcranial Direct Current Stimulation Combined with Physical Therapy-Induced Improvement in Individuals with Chronic Stroke. Neural Plast 2020;2020:8859394.

31. Bang DH, Bong SY. Effect of combination of transcranial direct current stimulation and feedback training on visuospatial neglect in patients with subacute stroke: a pilot randomized controlled trial. J Phys Ther Sci 2015;27:2759-61.

32. Bao SC, Wong WW, Leung TWH, Tong KY. Cortico-Muscular Coherence Modulated by High-Definition Transcranial Direct Current Stimulation in People With Chronic Stroke. IEEE Trans Neural Syst Rehabil Eng 2019;27:304-313.

33. Barker RN, Brauer SG, Barry BK, Gill TJ, Carson RG. Training-induced modifications of corticospinal reactivity in severely affected stroke survivors. Exp Brain Res 2012;221:211-21.

34. Barros Galvao SC, Borba Costa dos Santos R, Borba dos Santos P, Cabral ME, Monte-Silva K. Efficacy of coupling repetitive transcranial magnetic stimulation and physical therapy to reduce upper-limb spasticity in patients with stroke: a randomized controlled trial. Arch Phys Med Rehabil 2014;95:222-9.

35. Barwood CH, Murdoch BE, Riek S et al. Long term language recovery subsequent to low frequency rTMS in chronic non-fluent aphasia. NeuroRehabilitation 2013;32:915-28.

36. Barwood CHS, Murdoch BE, Whelan BM et al. Improved receptive and expressive language abilities in nonfluent aphasic stroke patients after application of rTMS: an open protocol case series. Brain Stimul 2012;5:274-286.

37. Batsikadze G, Paulus W, Kuo MF, Nitsche MA. Effect of serotonin on paired associative stimulation-induced plasticity in the human motor cortex. Neuropsychopharmacology 2013;38:2260-7.

38. Beaulieu LD, Blanchette AK, Mercier C, Bernard-Larocque V, Milot MH. Efficacy, safety, and tolerability of bilateral transcranial direct current stimulation combined to a resistance training program in chronic stroke survivors: A double-blind, randomized, placebo-controlled pilot study. Restor Neurol Neurosci 2019;37:333-346.

39. Beaulieu LD, Masse-Alarie H, Brouwer B, Schneider C. Noninvasive neurostimulation in chronic stroke: a double-blind randomized sham-controlled testing of clinical and corticomotor effects. Top Stroke Rehabil 2015;22:8-17.

40. Beaulieu LD, Masse-Alarie H, Camire-Bernier S, Ribot-Ciscar E, Schneider C. After-effects of peripheral neurostimulation on brain plasticity and ankle function in chronic stroke: The role of afferents recruited. Neurophysiol Clin 2017;47:275-291.

41. Berrigan P, Hodge J, Kirton A, Moretti ME, Ungar WJ, Zwicker JD. Protocol for a cost-utility analysis of neurostimulation and intensive camp-based therapy for children with perinatal stroke and hemiparesis based on a multicentre clinical trial. BMJ Open 2021;11:e041444.

42. Blesneag AV, Slavoaca DF, Popa L et al. Low-frequency rTMS in patients with subacute ischemic stroke: clinical evaluation of short and long-term outcomes and neurophysiological assessment of cortical excitability. J Med Life 2015;8:378-87.

43. Boasquevisque DS, Servinsckins L, de Paiva JPQ et al. Contralesional Cathodal Transcranial Direct Current Stimulation Does Not Enhance Upper Limb Function in Subacute Stroke: A Pilot Randomized Clinical Trial. Neural Plast 2021;2021:8858394.

44. Bolognini N, Russo C, Souza Carneiro MI et al. Bi-hemispheric transcranial direct current stimulation for upper-limb hemiparesis in acute stroke: a randomized, double-blind, sham-controlled trial. Eur J Neurol 2020;27:2473-2482.

45. Bonin Pinto C, Morales-Quezada L, de Toledo Piza PV et al. Combining Fluoxetine and rTMS in Poststroke Motor Recovery: A Placebo-Controlled Double-Blind Randomized Phase 2 Clinical Trial. Neurorehabil Neural Repair 2019;33:643-655.

46. Bonni S, Motta C, Pellicciari MC et al. Intermittent Cerebellar Theta Burst Stimulation Improves Visuo-motor Learning in Stroke Patients: a Pilot Study. Cerebellum 2020;19:739-743.

47. Borich MR, Wolf SL, Tan AQ, Palmer JA. Targeted Neuromodulation of Abnormal Interhemispheric Connectivity to Promote Neural Plasticity and Recovery of Arm Function after Stroke: A Randomized Crossover Clinical Trial Study Protocol. Neural Plast 2018;2018:9875326.

48. Bornheim S, Croisier JL, Maquet P, Kaux JF. Transcranial direct current stimulation associated with physical-therapy in acute stroke patients - A randomized, triple blind, sham-controlled study. Brain Stimul 2020;13:329-336.

49. Boyne P, Meyrose C, Westover J et al. Exercise intensity affects acute neurotrophic and neurophysiological responses poststroke. J Appl Physiol (1985) 2019;126:431-443.

50. Branscheidt M, Hoppe J, Zwitserlood P, Liuzzi G. tDCS over the motor cortex improves lexical retrieval of action words in poststroke aphasia. J Neurophysiol 2018;119:621-630.

51. Brodie SM, Borich MR, Boyd LA. Impact of 5-Hz rTMS over the primary sensory cortex is related to white matter volume in individuals with chronic stroke. Eur J Neurosci 2014;40:3405-12.

52. Brodie SM, Meehan S, Borich MR, Boyd LA. 5 Hz repetitive transcranial magnetic stimulation over the ipsilesional sensory cortex enhances motor learning after stroke. Front Hum Neurosci 2014;8:143.

53. Buick AR, Kowalczewski J, Carson RG, Prochazka A. Tele-Supervised FES-Assisted Exercise for Hemiplegic Upper Limb. IEEE Trans Neural Syst Rehabil Eng 2016;24:79-87.

54. Cabib C, Nascimento W, Rofes L et al. Short-term neurophysiological effects of sensory pathway neurorehabilitation strategies on chronic poststroke oropharyngeal dysphagia. Neurogastroenterol Motil 2020;32:e13887.

55. Calabro RS, Naro A, Russo M et al. Shaping neuroplasticity by using powered exoskeletons in patients with stroke: a randomized clinical trial. J Neuroeng Rehabil 2018;15:35.

56. Cao H, Chen X, Ren X et al. Repetitive transcranial magnetic stimulation combined with respiratory muscle training for pulmonary rehabilitation after ischemic stroke-A randomized, case-control study. Front Aging Neurosci 2022;14:1006696.

57. Carlson HL, Ciechanski P, Harris AD, MacMaster FP, Kirton A. Changes in spectroscopic biomarkers after transcranial direct current stimulation in children with perinatal stroke. Brain Stimul 2018;11:94-103.

58. Cassidy JM, Chu H, Anderson DC et al. A Comparison of Primed Low-frequency Repetitive Transcranial Magnetic Stimulation Treatments in Chronic Stroke. Brain Stimul 2015;8:1074-84.

59. Cattagni T, Geiger M, Supiot A et al. A single session of anodal transcranial direct current stimulation applied over the affected primary motor cortex does not alter gait parameters in chronic stroke survivors. Neurophysiol Clin 2019;49:283-293.

60. Cazzoli D, Muri RM, Schumacher R et al. Theta burst stimulation reduces disability during the activities of daily living in spatial neglect. Brain 2012;135:3426-39.

61. Cha B, Kim J, Kim JM et al. Therapeutic Effect of Repetitive Transcranial Magnetic Stimulation for Post-stroke Vascular Cognitive Impairment: A Prospective Pilot Study. Front Neurol 2022;13:813597.

62. Cha HG, Kim MK. The effects of repetitive transcranial magnetic stimulation on unilateral neglect of acute stroke patients: A randomised controlled trial. Hong Kong Physiother J 2015;33:53-58.

63. Cha HG, Kim MK. Effects of repetitive transcranial magnetic stimulation on arm function and decreasing unilateral spatial neglect in subacute stroke: a randomized controlled trial. Clin Rehabil 2016;30:649-56.

64. Cha HG, Kim MK. Effects of strengthening exercise integrated repetitive transcranial magnetic stimulation on motor function recovery in subacute stroke patients: A randomized controlled trial. Technol Health Care 2017;25:521-529.

65. Cha HK, Ji SG, Kim MK, Chang JS. Effect of transcranial direct current stimulation of function in patients with stroke. J Phys Ther Sci 2014;26:363-5.

66. Chabriat H, Bassetti CL, Marx U et al. Safety and efficacy of GABA(A) alpha5 antagonist S44819 in patients with ischaemic stroke: a multicentre, double-blind, randomised, placebo-controlled trial. Lancet Neurol 2020;19:226-233.

67. Chagas TJ, Cravo I, Bazan R, de Souza L, Luvizutto GJ. Effects of transcranial direct current stimulation on balance after ischemic stroke (SANDE trial): Study protocol for a multicentric randomized controlled trial. Contemp Clin Trials 2021;105:106396.

68. Chang MC, Kim DY, Park DH. Enhancement of Cortical Excitability and Lower Limb Motor Function in Patients With Stroke by Transcranial Direct Current Stimulation. Brain Stimul 2015;8:561-6.

69. Chang PW, Lu CF, Chang ST, Tsai PY. Functional Near-Infrared Spectroscopy as a Target Navigator for rTMS Modulation in Patients with Hemiplegia: A Randomized Control Study. Neurol Ther 2022;11:103-121.

70. Chang WH, Kim YH, Yoo WK et al. rTMS with motor training modulates cortico-basal ganglia-thalamocortical circuits in stroke patients. Restor Neurol Neurosci 2012;30:179-89.

71. Chen J, Shen K, Fan L et al. Integrative medicine in treating post-stroke depression: Study protocol for a multicenter, prospective, randomized, controlled trial. Front Psychol 2022;13:923506.

72. Chen Q, Huang H, Chen G et al. The Effect of Cerebellar Repetitive Transcranial Magnetic Stimulation on Dysphagia due to Posterior Circulation Stroke, a Randomized Controlled Trial Protocol. Cerebrovasc Dis 2022;51:706-711.

73. Chen Q, Shen D, Sun H et al. Effects of coupling inhibitory and facilitatory repetitive transcranial magnetic stimulation on motor recovery in patients following acute cerebral infarction. NeuroRehabilitation 2021;48:83-96.

74. Chen Q, Shen W, Sun H et al. The effect of coupled inhibitory-facilitatory repetitive transcranial magnetic stimulation on shaping early reorganization of the motor network after stroke. Brain Res 2022;1790:147959.

75. Chen QM, Yao FR, Sun HW et al. Combining inhibitory and facilitatory repetitive transcranial magnetic stimulation (rTMS) treatment improves motor function by modulating GABA in acute ischemic stroke patients. Restor Neurol Neurosci 2021;39:419-434.

76. Chen SC, Yang LY, Adeel M, Lai CH, Peng CW. Transcranial electrostimulation with special waveforms enhances upper-limb motor function in patients with chronic stroke: a pilot randomized controlled trial. J Neuroeng Rehabil 2021;18:106.

77. Chen X, Liu X, Cui Y et al. Efficacy of functional magnetic stimulation in improving upper extremity function after stroke: a randomized, single-blind, controlled study. J Int Med Res 2020;48:300060520927881.

78. Chen Y, Wang C, Song P et al. Alpha rhythm of electroencephalography was modulated differently by three transcranial direct current stimulation protocols in patients with ischemic stroke. Front Hum Neurosci 2022;16:887849.

79. Chen YJ, Huang YZ, Chen CY et al. Intermittent theta burst stimulation enhances upper limb motor function in patients with chronic stroke: a pilot randomized controlled trial. BMC Neurol 2019;19:69.

80. Cheng IK, Chan KM, Wong CS, Cheung RT. Preliminary evidence of the effects of high-frequency repetitive transcranial magnetic stimulation (rTMS) on swallowing functions in post-stroke individuals with chronic dysphagia. Int J Lang Commun Disord 2015;50:389-96.

81. Cheng IKY, Chan KMK, Wong CS et al. Neuronavigated high-frequency repetitive transcranial magnetic stimulation for chronic post-stroke dysphagia: A randomized controlled study. J Rehabil Med 2017;49:475-481.

82. Cherney LR. Epidural Cortical Stimulation as Adjunctive Treatment for Nonfluent Aphasia: Phase 1 Clinical Trial Follow-up Findings. Neurorehabil Neural Repair 2016;30:131-42.

83. Cherney LR, Babbitt EM, Wang X, Pitts LL. Extended fMRI-Guided Anodal and Cathodal Transcranial Direct Current Stimulation Targeting Perilesional Areas in Post-Stroke Aphasia: A Pilot Randomized Clinical Trial. Brain Sci 2021;11.

84. Chervyakov AV, Poydasheva AG, Lyukmanov RH et al. Effects of Navigated Repetitive Transcranial Magnetic Stimulation After Stroke. J Clin Neurophysiol 2018;35:166-172.

85. Chew E, Teo WP, Tang N et al. Using Transcranial Direct Current Stimulation to Augment the Effect of Motor Imagery-Assisted Brain-Computer Interface Training in Chronic Stroke Patients-Cortical Reorganization Considerations. Front Neurol 2020;11:948.

86. Chhatbar PY, Ramakrishnan V, Kautz S, George MS, Adams RJ, Feng W. Transcranial Direct Current Stimulation Post-Stroke Upper Extremity Motor Recovery Studies Exhibit a Dose-Response Relationship. Brain Stimul 2016;9:16-26.

87. Chieffo R, De Prezzo S, Houdayer E et al. Deep repetitive transcranial magnetic stimulation with H-coil on lower limb motor function in chronic stroke: a pilot study. Arch Phys Med Rehabil 2014;95:1141-7.

88. Chieffo R, Ferrari F, Battista P et al. Excitatory deep transcranial magnetic stimulation with H-coil over the right homologous Broca's region improves naming in chronic post-stroke aphasia. Neurorehabil Neural Repair 2014;28:291-8.

89. Chieffo R, Giatsidis F, Santangelo R et al. Repetitive Transcranial Magnetic Stimulation With H-Coil Coupled With Cycling for Improving Lower Limb Motor Function After Stroke: An Exploratory Study. Neuromodulation 2021;24:916-922.

90. Chiu D, McCane CD, Lee J et al. Multifocal transcranial stimulation in chronic ischemic stroke: A phase 1/2a randomized trial. J Stroke Cerebrovasc Dis 2020;29:104816.

91. Cho HS, Cha HG. Effect of mirror therapy with tDCS on functional recovery of the upper extremity of stroke patients. J Phys Ther Sci 2015;27:1045-7.

92. Cho JY, Lee A, Kim MS et al. Dual-mode noninvasive brain stimulation over the bilateral primary motor cortices in stroke patients. Restor Neurol Neurosci 2017;35:105-114.

93. Choi GS, Chang MC. Effects of high-frequency repetitive transcranial magnetic stimulation on reducing hemiplegic shoulder pain in patients with chronic stoke: a randomized controlled trial. Int J Neurosci 2018;128:110-116.

94. Chou TY, Wang JC, Lin MY, Tsai PY. Low-Frequency vs. Theta Burst Transcranial Magnetic Stimulation for the Treatment of Chronic Non-fluent Aphasia in Stroke: A Proof-of-Concept Study. Front Aging Neurosci 2021;13:800377.

95. Chu M, Zhang Y, Chen J et al. Efficacy of Intermittent Theta-Burst Stimulation and Transcranial Direct Current Stimulation in Treatment of Post-Stroke Cognitive Impairment. J Integr Neurosci 2022;21:130.

96. Chung E, Kim JH, Park DS, Lee BH. Effects of brain-computer interface-based functional electrical stimulation on brain activation in stroke patients: a pilot randomized controlled trial. J Phys Ther Sci 2015;27:559-62.

97. Clancy JA, Johnson R, Raw R, Deuchars SA, Deuchars J. Anodal transcranial direct current stimulation (tDCS) over the motor cortex increases sympathetic nerve activity. Brain Stimul 2014;7:97-104.

98. Cleland BT, Sisel E, Madhavan S. Motor evoked potential latency and duration from tibialis anterior in individuals with chronic stroke. Exp Brain Res 2021;239:2251-2260.

99. Clemens B, Jung S, Zvyagintsev M, Domahs F, Willmes K. Modulating arithmetic fact retrieval: a single-blind, sham-controlled tDCS study with repeated fMRI measurements. Neuropsychologia 2013;51:1279-86.

100. Conforto AB, Anjos SM, Saposnik G et al. Transcranial magnetic stimulation in mild to severe hemiparesis early after stroke: a proof of principle and novel approach to improve motor function. J Neurol 2012;259:1399-405.

101. Cotelli M, Manenti R, Petesi M et al. Treatment of primary progressive aphasias by transcranial direct current stimulation combined with language training. J Alzheimers Dis 2014;39:799-808.

102. Cunningham DA, Knutson JS, Sankarasubramanian V, Potter-Baker KA, Machado AG, Plow EB. Bilateral Contralaterally Controlled Functional Electrical Stimulation Reveals New Insights Into the Interhemispheric Competition Model in Chronic Stroke. Neurorehabil Neural Repair 2019;33:707-717.

103. Cunningham DA, Varnerin N, Machado A et al. Stimulation targeting higher motor areas in stroke rehabilitation: A proof-of-concept, randomized, double-blinded placebo-controlled study of effectiveness and underlying mechanisms. Restor Neurol Neurosci 2015;33:911-26.

104. Curado M, Fritsch B, Reis J. Non-Invasive Electrical Brain Stimulation Montages for Modulation of Human Motor Function. J Vis Exp 2016:e53367.

105. D'Agata F, Peila E, Cicerale A et al. Cognitive and Neurophysiological Effects of Non-invasive Brain Stimulation in Stroke Patients after Motor Rehabilitation. Front Behav Neurosci 2016;10:135.

106. da Cunha MJ, Pinto C, Schifino GP, Sant'Anna Py I, Cimolin V, Pagnussat AS. Bicephalic Transcranial Direct-Current Stimulation Does Not Add Benefits to a Footdrop Stimulator for Improving Functional Mobility in People With Chronic Hemiparesis After Stroke: A Double-Blind, Randomized Controlled Trial. Phys Ther 2022;102.

107. da Silva TR, de Carvalho Nunes HR, Martins LG et al. Non-invasive Brain Stimulation Can Reduce Unilateral Spatial Neglect after Stroke: ELETRON Trial. Ann Neurol 2022;92:400-410.

108. Danzl MM, Chelette KC, Lee K, Lykins D, Sawaki L. Brain stimulation paired with novel locomotor training with robotic gait orthosis in chronic stroke: a feasibility study. NeuroRehabilitation 2013;33:67-76.

109. Darkow R, Martin A, Wurtz A, Floel A, Meinzer M. Transcranial direct current stimulation effects on neural processing in post-stroke aphasia. Hum Brain Mapp 2017;38:1518-1531.

110. Darmani G, Zipser CM, Bohmer GM et al. Effects of the Selective alpha5-GABAAR Antagonist S44819 on Excitability in the Human Brain: A TMS-EMG and TMS-EEG Phase I Study. J Neurosci 2016;36:12312-12320.

111. Davis NJ, Gold E, Pascual-Leone A, Bracewell RM. Challenges of proper placebo control for non-invasive brain stimulation in clinical and experimental applications. Eur J Neurosci 2013;38:2973-7.

112. De Doncker W, Ondobaka S, Kuppuswamy A. Effect of transcranial direct current stimulation on post-stroke fatigue. J Neurol 2021;268:2831-2842.

113. de Freitas Zanona A, Romeiro da Silva AC, do Rego Maciel AB et al. Somatosensory Cortex Repetitive Transcranial Magnetic Stimulation and Associative Sensory Stimulation of Peripheral Nerves Could Assist Motor and Sensory Recovery After Stroke. Front Hum Neurosci 2022;16:860965.

114. De Laet C, Herman B, Riga A et al. Bimanual motor skill learning after stroke: Combining robotics and anodal tDCS over the undamaged hemisphere: An exploratory study. Front Neurol 2022;13:882225.

115. de Oliveira RA, de Andrade DC, Mendonca M et al. Repetitive transcranial magnetic stimulation of the left premotor/dorsolateral prefrontal cortex does not have analgesic effect on central poststroke pain. J Pain 2014;15:1271-81.

116. de Souza JA, Correa JCF, Agnol LD, Dos Santos FR, Gomes MRP, Correa FI. Effects of transcranial direct current stimulation on the rehabilitation of painful shoulder following a stroke: protocol for a randomized, controlled, double-blind, clinical trial. Trials 2019;20:165.

117. Dehem S, Gilliaux M, Lejeune T et al. Effectiveness of a single session of dual-transcranial direct current stimulation in combination with upper limb robotic-assisted rehabilitation in chronic stroke patients: a randomized, double-blind, cross-over study. Int J Rehabil Res 2018;41:138-145.

118. Del Felice A, Daloli V, Masiero S, Manganotti P. Contralesional Cathodal versus Dual Transcranial Direct Current Stimulation for Decreasing Upper Limb Spasticity in Chronic Stroke Individuals: A Clinical and Neurophysiological Study. J Stroke Cerebrovasc Dis 2016;25:2932-2941.

119. Di Gregorio F, La Porta F, Casanova E et al. Efficacy of repetitive transcranial magnetic stimulation combined with visual scanning treatment on cognitive and behavioral symptoms of left hemispatial neglect in right hemispheric stroke patients: study protocol for a randomized controlled trial. Trials 2021;22:24.

120. Di Lazzaro V, Capone F, Di Pino G et al. Combining Robotic Training and Non-Invasive Brain Stimulation in Severe Upper Limb-Impaired Chronic Stroke Patients. Front Neurosci 2016;10:88.

121. Di Lazzaro V, Dileone M, Capone F et al. Immediate and late modulation of interhemipheric imbalance with bilateral transcranial direct current stimulation in acute stroke. Brain Stimul 2014;7:841-8.

122. Di Lazzaro V, Rothwell JC, Talelli P et al. Inhibitory theta burst stimulation of affected hemisphere in chronic stroke: a proof of principle, sham-controlled study. Neurosci Lett 2013;553:148-52.

123. Dimyan MA, Perez MA, Auh S, Tarula E, Wilson M, Cohen LG. Nonparetic arm force does not overinhibit the paretic arm in chronic poststroke hemiparesis. Arch Phys Med Rehabil 2014;95:849-56.

124. Ding Q, Chen S, Chen J et al. Intermittent Theta Burst Stimulation Increases Natural Oscillatory Frequency in Ipsilesional Motor Cortex Post-Stroke: A Transcranial Magnetic Stimulation and Electroencephalography Study. Front Aging Neurosci 2022;14:818340.

125. Ding Q, Zhang S, Chen S et al. The Effects of Intermittent Theta Burst Stimulation on Functional Brain Network Following Stroke: An Electroencephalography Study. Front Neurosci 2021;15:755709.

126. Dionisio A, Gouveia R, Castelhano J et al. The Role of Continuous Theta Burst TMS in the Neurorehabilitation of Subacute Stroke Patients: A Placebo-Controlled Study. Front Neurol 2021;12:749798.

127. Divya M, Narkeesh A. Therapeutic Effect of Multi-Channel Transcranial Direct Current Stimulation (M-tDCS) on Recovery of Cognitive Domains, Motor Functions of Paretic Hand and Gait in Subacute Stroke Survivors-A Randomized Controlled Trial Protocol. Neurosci Insights 2022;17:26331055221087741.

128. Dong XL, Sun X, Sun WM et al. A randomized controlled trial to explore the efficacy and safety of transcranial direct current stimulation on patients with post-stroke fatigue. Medicine (Baltimore) 2021;100:e27504.

129. Doost MY, Orban de Xivry JJ, Herman B et al. Learning a Bimanual Cooperative Skill in Chronic Stroke Under Noninvasive Brain Stimulation: A Randomized Controlled Trial. Neurorehabil Neural Repair 2019;33:486-498.

130. Doris Miu KY, Kok C, Leung SS, Chan EYL, Wong E. Comparison of Repetitive Transcranial Magnetic Stimulation and Transcranial Direct Current Stimulation on Upper Limb Recovery Among Patients With Recent Stroke. Ann Rehabil Med 2020;44:428-437.

131. Dos Santos RBC, Galvao SCB, Frederico LMP et al. Cortical and spinal excitability changes after repetitive transcranial magnetic stimulation combined to physiotherapy in stroke spastic patients. Neurol Sci 2019;40:1199-1207.

132. Du J, Tian L, Liu W et al. Effects of repetitive transcranial magnetic stimulation on motor recovery and motor cortex excitability in patients with stroke: a randomized controlled trial. Eur J Neurol 2016;23:1666-1672.

133. Du J, Wang S, Cheng Y et al. Effects of Neuromuscular Electrical Stimulation Combined with Repetitive Transcranial Magnetic Stimulation on Upper Limb Motor Function Rehabilitation in Stroke Patients with Hemiplegia. Comput Math Methods Med 2022;2022:9455428.

134. Du J, Yang F, Hu J et al. Effects of high- and low-frequency repetitive transcranial magnetic stimulation on motor recovery in early stroke patients: Evidence from a randomized controlled trial with clinical, neurophysiological and functional imaging assessments. Neuroimage Clin 2019;21:101620.

135. Du J, Yang F, Liu L et al. Repetitive transcranial magnetic stimulation for rehabilitation of poststroke dysphagia: A randomized, double-blind clinical trial. Clin Neurophysiol 2016;127:1907-13.

136. Du Y, Wei L, Lu Y, Gao H. The effects of different frequencies of repetitive transcranial magnetic stimulation (rTMS) on patients with swallowing disorders after cerebral infarction. NeuroRehabilitation 2022;50:115-122.

137. Duan YJ, Hua XY, Zheng MX et al. Corticocortical paired associative stimulation for treating motor dysfunction after stroke: study protocol for a randomised sham-controlled double-blind clinical trial. BMJ Open 2022;12:e053991.

138. Edwards DJ, Cortes M, Rykman-Peltz A et al. Clinical improvement with intensive robot-assisted arm training in chronic stroke is unchanged by supplementary tDCS. Restor Neurol Neurosci 2019;37:167-180.

139. Ehsani F, Mortezanejad M, Yosephi MH, Daniali S, Jaberzadeh S. The effects of concurrent M1 anodal tDCS and physical therapy interventions on function of ankle muscles in patients with stroke: a randomized, double-blinded sham-controlled trial study. Neurol Sci 2022;43:1893-1901.

140. El Nahas N, Elbokl AM, Abd Eldayem EH et al. Navigated perilesional transcranial magnetic stimulation can improve post-stroke visual field defect: A double-blind sham-controlled study. Restor Neurol Neurosci 2021;39:199-207.

141. El-Tamawy MS, Darwish MH, Elkholy SH, Moustafa EBS, Abulkassem ST, Khalifa HA. Low frequency transcranial magnetic stimulation in subacute ischemic stroke: Number of sessions that altered cortical excitability. NeuroRehabilitation 2020;47:427-434.

142. Elsner B, Kugler J, Pohl M, Mehrholz J. Transcranial direct current stimulation (tDCS) for improving activities of daily living, and physical and cognitive functioning, in people after stroke. Cochrane Database Syst Rev 2020;11:CD009645.

143. Etherton-Beer C, Lui Y, Radalj M, Vallence AM, Singer B. Transcranial Direct Current Stimulation to Optimise Participation in Stroke Rehabilitation - A Sham-Controlled Cross-Over Feasibility Study. Neurosci Insights 2020;15:2633105520922181.

144. Etoh S, Kawamura K, Tomonaga K et al. Effects of concomitant neuromuscular electrical stimulation during repetitive transcranial magnetic stimulation before repetitive facilitation exercise on the hemiparetic hand. NeuroRehabilitation 2019;45:323-329.

145. Etoh S, Noma T, Ikeda K et al. Effects of repetitive trascranial magnetic stimulation on repetitive facilitation exercises of the hemiplegic hand in chronic stroke patients. J Rehabil Med 2013;45:843-7.

146. Fan J, Voisin J, Milot MH, Higgins J, Boudrias MH. Transcranial direct current stimulation over multiple days enhances motor performance of a grip task. Ann Phys Rehabil Med 2017;60:329-333.

147. Feil S, Eisenhut P, Strakeljahn F et al. Left Shifting of Language Related Activity Induced by Bihemispheric tDCS in Postacute Aphasia Following Stroke. Front Neurosci 2019;13:295.

148. Ferreira IS, Pinto CB, Saleh Velez FG, Leffa DT, Vulcano de Toledo Piza P, Fregni F. Recruitment challenges in stroke neurorecovery clinical trials. Contemp Clin Trials Commun 2019;15:100404.

149. Figlewski K, Blicher JU, Mortensen J, Severinsen KE, Nielsen JF, Andersen H. Transcranial Direct Current Stimulation Potentiates Improvements in Functional Ability in Patients With Chronic Stroke Receiving Constraint-Induced Movement Therapy. Stroke 2017;48:229-232.

150. Fiori V, Nitsche M, Iasevoli L, Cucuzza G, Caltagirone C, Marangolo P. Differential effects of bihemispheric and unihemispheric transcranial direct current stimulation in young and elderly adults in verbal learning. Behav Brain Res 2017;321:170-175.

151. Fleming MK, Sorinola IO, Roberts-Lewis SF, Wolfe CD, Wellwood I, Newham DJ. The effect of combined somatosensory stimulation and task-specific training on upper limb function in chronic stroke: a double-blind randomized controlled trial. Neurorehabil Neural Repair 2015;29:143-52.

152. Forogh B, Ahadi T, Nazari M et al. The Effect of Repetitive Transcranial Magnetic Stimulation on Postural Stability After Acute Stroke: A Clinical Trial. Basic Clin Neurosci 2017;8:405-411.

153. Frey J, Najib U, Lilly C, Adcock A. Novel TMS for Stroke and Depression (NoTSAD): Accelerated Repetitive Transcranial Magnetic Stimulation as a Safe and Effective Treatment for Post-stroke Depression. Front Neurol 2020;11:788.

154. Fridriksson J, Elm J, Stark BC et al. BDNF genotype and tDCS interaction in aphasia treatment. Brain Stimul 2018;11:1276-1281.

155. Fridriksson J, Rorden C, Elm J, Sen S, George MS, Bonilha L. Transcranial Direct Current Stimulation vs Sham Stimulation to Treat Aphasia After Stroke: A Randomized Clinical Trial. JAMA Neurol 2018;75:1470-1476.

156. Fruhauf AMA, Politti F, Dal Corso S et al. Immediate effect of transcranial direct current stimulation combined with functional electrical stimulation on activity of the tibialis anterior muscle and balance of individuals with hemiparesis stemming from a stroke. J Phys Ther Sci 2017;29:2138-2146.

157. Fu W, Song W, Zhang Y et al. Long-term effects of continuous theta-burst stimulation in visuospatial neglect. J Int Med Res 2015;43:196-203.

158. Fusco A, Assenza F, Iosa M et al. The ineffective role of cathodal tDCS in enhancing the functional motor outcomes in early phase of stroke rehabilitation: an experimental trial. Biomed Res Int 2014;2014:547290.

159. Fusco A, De Angelis D, Morone G et al. The ABC of tDCS: Effects of Anodal, Bilateral and Cathodal Montages of Transcranial Direct Current Stimulation in Patients with Stroke-A Pilot Study. Stroke Res Treat 2013;2013:837595.

160. Fusco A, Iosa M, Venturiero V et al. After vs. priming effects of anodal transcranial direct current stimulation on upper extremity motor recovery in patients with subacute stroke. Restor Neurol Neurosci 2014;32:301-12.

161. Gall C, Silvennoinen K, Granata G et al. Non-invasive electric current stimulation for restoration of vision after unilateral occipital stroke. Contemp Clin Trials 2015;43:231-6.

162. Garcia G, Norise C, Faseyitan O, Naeser MA, Hamilton RH. Utilizing repetitive transcranial magnetic stimulation to improve language function in stroke patients with chronic non-fluent aphasia. J Vis Exp 2013:e50228.

163. Geiger M, Roche N, Vlachos E, Cattagni T, Zory R. Acute effects of bi-hemispheric transcranial direct current stimulation on the neuromuscular function of patients with chronic stroke: A randomized controlled study. Clin Biomech (Bristol, Avon) 2019;70:1-7.

164. Geiger M, Supiot A, Zory R, Aegerter P, Pradon D, Roche N. The effect of transcranial direct current stimulation (tDCS) on locomotion and balance in patients with chronic stroke: study protocol for a randomised controlled trial. Trials 2017;18:492.

165. Gillick B, Menk J, Mueller B et al. Synergistic effect of combined transcranial direct current stimulation/constraint-induced movement therapy in children and young adults with hemiparesis: study protocol. BMC Pediatr 2015;15:178.

166. Gillick BT, Feyma T, Menk J et al. Safety and feasibility of transcranial direct current stimulation in pediatric hemiparesis: randomized controlled preliminary study. Phys Ther 2015;95:337-49.

167. Gillick BT, Krach LE, Feyma T et al. Safety of primed repetitive transcranial magnetic stimulation and modified constraint-induced movement therapy in a randomized controlled trial in pediatric hemiparesis. Arch Phys Med Rehabil 2015;96:S104-13.

168. Gillick BT, Krach LE, Feyma T et al. Primed low-frequency repetitive transcranial magnetic stimulation and constraint-induced movement therapy in pediatric hemiparesis: a randomized controlled trial. Dev Med Child Neurol 2014;56:44-52.

169. Goh HT, Chan HY, Abdul-Latif L. Aftereffects of 2 noninvasive brain stimulation techniques on corticospinal excitability in persons with chronic stroke: a pilot study. J Neurol Phys Ther 2015;39:15-22.

170. Gong Y, Long XM, Xu Y, Cai XY, Ye M. Effects of repetitive transcranial magnetic stimulation combined with transcranial direct current stimulation on motor function and cortex excitability in subacute stroke patients: A randomized controlled trial. Clin Rehabil 2021;35:718-727.

171. Gopalakrishnan R, Burgess RC, Malone DA et al. Deep brain stimulation of the ventral striatal area for poststroke pain syndrome: a magnetoencephalography study. J Neurophysiol 2018;119:2118-2128.

172. Gorsler A, Grittner U, Kulzow N, Rackoll T. Blinding in electric current stimulation in subacute neglect patients with current densities of 0.8 A/m(2): a cross-over pilot study. BMC Res Notes 2021;14:35.

173. Gottlieb A, Boltzmann M, Schmidt SB et al. Treatment of upper limb spasticity with inhibitory repetitive transcranial magnetic stimulation: A randomized placebo-controlled trial. NeuroRehabilitation 2021;49:425-434.

174. Group NTC. A multicenter, randomized, double-blind, placebo-controlled trial to test efficacy and safety of transcranial direct current stimulation to the motor cortex after stroke (NETS): study protocol. Neurol Res Pract 2022;4:14.

175. Gu SY, Chang MC. The Effects of 10-Hz Repetitive Transcranial Magnetic Stimulation on Depression in Chronic Stroke Patients. Brain Stimul 2017;10:270-274.

176. Guan YZ, Li J, Zhang XW et al. Effectiveness of repetitive transcranial magnetic stimulation (rTMS) after acute stroke: A one-year longitudinal randomized trial. CNS Neurosci Ther 2017;23:940-946.

177. Guillouet E, Cogne M, Saverot E et al. Impact of Combined Transcranial Direct Current Stimulation and Speech-language Therapy on Spontaneous Speech in Aphasia: A Randomized Controlled Double-blind Study. J Int Neuropsychol Soc 2020;26:7-18.

178. Haghighi M, Mazdeh M, Ranjbar N, Seifrabie MA. Further Evidence of the Positive Influence of Repetitive Transcranial Magnetic Stimulation on Speech and Language in Patients with Aphasia after Stroke: Results from a Double-Blind Intervention with Sham Condition. Neuropsychobiology 2017;75:185-192.

179. Halakoo S, Ehsani F, Masoudian N, Zoghi M, Jaberzadeh S. Does anodal trans-cranial direct current stimulation of the damaged primary motor cortex affects wrist flexor muscle spasticity and also activity of the wrist flexor and extensor muscles in patients with stroke?: a Randomized Clinical Trial. Neurol Sci 2021;42:2763-2773.

180. Hammad AB, Elhamrawy EA, Abdel-Tawab H et al. Transcranial Magnetic Stimulation Versus Transcutaneous Neuromuscular Electrical Stimulation in Post Stroke Dysphagia: A Clinical Randomized Controlled Trial. J Stroke Cerebrovasc Dis 2022;31:106554.

181. Hammerbeck U, Tyson SF, Samraj P, Hollands K, Krakauer JW, Rothwell J. The Strength of the Corticospinal Tract Not the Reticulospinal Tract Determines Upper-Limb Impairment Level and Capacity for Skill-Acquisition in the Sub-Acute Post-Stroke Period. Neurorehabil Neural Repair 2021;35:812-822.

182. Hamoudi M, Schambra HM, Fritsch B et al. Transcranial Direct Current Stimulation Enhances Motor Skill Learning but Not Generalization in Chronic Stroke. Neurorehabil Neural Repair 2018;32:295-308.

183. Han JY, Kim JH, Park JH et al. Scalp acupuncture and electromagnetic convergence stimulation for patients with cerebral infarction: study protocol for a randomized controlled trial. Trials 2016;17:490.

184. Hanafi MH, Kassim NK, Ibrahim AH et al. Cortical Modulation After Two Different Repetitive Transcranial Magnetic Stimulation Protocols in Similar Ischemic Stroke Patients. Malays J Med Sci 2018;25:116-125.

185. Harvey RL, Edwards D, Dunning K et al. Randomized Sham-Controlled Trial of Navigated Repetitive Transcranial Magnetic Stimulation for Motor Recovery in Stroke. Stroke 2018;49:2138-2146.

186. Hayward KS, Brauer SG, Ruddy KL, Lloyd D, Carson RG. Repetitive reaching training combined with transcranial Random Noise Stimulation in stroke survivors with chronic and severe arm paresis is feasible: a pilot, triple-blind, randomised case series. J Neuroeng Rehabil 2017;14:46.

187. He XK, Sun QQ, Liu HH, Guo XY, Chen C, Chen LD. Timing of Acupuncture during LTP-Like Plasticity Induced by Paired-Associative Stimulation. Behav Neurol 2019;2019:9278270.

188. Heikkinen PH, Pulvermuller F, Makela JP et al. Combining rTMS With Intensive Language-Action Therapy in Chronic Aphasia: A Randomized Controlled Trial. Front Neurosci 2018;12:1036.

189. Heinz G, De Angelis K, Dal Corso S et al. Effects of Transcranial Direct Current Stimulation (tDCS) and Exercises Treadmill on Autonomic Modulation of Hemiparetic Patients Due To Stroke-Clinic Test, Controlled, Randomized, Double-Blind. Front Neurol 2019;10:1402.

190. Heiss WD, Hartmann A, Rubi-Fessen I et al. Noninvasive brain stimulation for treatment of right- and left-handed poststroke aphasics. Cerebrovasc Dis 2013;36:363-72.

191. Hensel L, Grefkes C, Tscherpel C et al. Intermittent theta burst stimulation applied during early rehabilitation after stroke: study protocol for a randomised controlled trial. BMJ Open 2019;9:e034088.

192. Hensel L, Tscherpel C, Freytag J et al. Connectivity-Related Roles of Contralesional Brain Regions for Motor Performance Early after Stroke. Cereb Cortex 2021;31:993-1007.

193. Higgins J, Koski L, Xie H. Combining rTMS and Task-Oriented Training in the Rehabilitation of the Arm after Stroke: A Pilot Randomized Controlled Trial. Stroke Res Treat 2013;2013:539146.

194. Hirakawa Y, Takeda K, Tanabe S et al. Effect of intensive motor training with repetitive transcranial magnetic stimulation on upper limb motor function in chronic post-stroke patients with severe upper limb motor impairment. Top Stroke Rehabil 2018;25:321-325.

195. Hodics TM, Nakatsuka K, Upreti B, Alex A, Smith PS, Pezzullo JC. Wolf Motor Function Test for characterizing moderate to severe hemiparesis in stroke patients. Arch Phys Med Rehabil 2012;93:1963-7.

196. Hong X, Lu ZK, Teh I et al. Brain plasticity following MI-BCI training combined with tDCS in a randomized trial in chronic subcortical stroke subjects: a preliminary study. Sci Rep 2017;7:9222.

197. Hordacre B, Comacchio K, Williams L, Hillier S. Repetitive transcranial magnetic stimulation for post-stroke depression: a randomised trial with neurophysiological insight. J Neurol 2021;268:1474-1484.

198. Hordacre B, Moezzi B, Ridding MC. Neuroplasticity and network connectivity of the motor cortex following stroke: A transcranial direct current stimulation study. Hum Brain Mapp 2018;39:3326-3339.

199. Hosomi K, Morris S, Sakamoto T et al. Daily Repetitive Transcranial Magnetic Stimulation for Poststroke Upper Limb Paresis in the Subacute Period. J Stroke Cerebrovasc Dis 2016;25:1655-1664.

200. Hu M, Cheng HJ, Ji F et al. Brain Functional Changes in Stroke Following Rehabilitation Using Brain-Computer Interface-Assisted Motor Imagery With and Without tDCS: A Pilot Study. Front Hum Neurosci 2021;15:692304.

201. Hu XY, Zhang T, Rajah GB et al. Effects of different frequencies of repetitive transcranial magnetic stimulation in stroke patients with non-fluent aphasia: a randomized, sham-controlled study. Neurol Res 2018;40:459-465.

202. Huang YJ, Wang SM, Chen C et al. High-Definition Transcranial Direct Current with Electrical Theta Burst on Post-Stroke Motor Rehabilitation: A Pilot Randomized Controlled Trial. Neurorehabil Neural Repair 2022;36:645-654.

203. Huang YZ, Lin LF, Chang KH, Hu CJ, Liou TH, Lin YN. Priming With 1-Hz Repetitive Transcranial Magnetic Stimulation Over Contralesional Leg Motor Cortex Does Not Increase the Rate of Regaining Ambulation Within 3 Months of Stroke: A Randomized Controlled Trial. Am J Phys Med Rehabil 2018;97:339-345.

204. Hunter SM, Johansen-Berg H, Ward N et al. Functional Strength Training and Movement Performance Therapy for Upper Limb Recovery Early Poststroke-Efficacy, Neural Correlates, Predictive Markers, and Cost-Effectiveness: FAST-INdiCATE Trial. Front Neurol 2017;8:733.

205. Hurd C, Livingstone D, Brunton K et al. Early Intensive Leg Training to Enhance Walking in Children With Perinatal Stroke: Protocol for a Randomized Controlled Trial. Phys Ther 2017;97:818-825.

206. Hyakutake K, Morishita T, Saita K et al. Feasibility of single and combined with other treatments using transcranial direct current stimulation for chronic stroke: A pilot study. SAGE Open Med 2020;8:2050312120940546.

207. Ihl T, Ahmadi M, Laumeier I et al. Patient-Centered Outcomes in a Randomized Trial Investigating a Multimodal Prevention Program After Transient Ischemic Attack or Minor Stroke: The INSPiRE-TMS Trial. Stroke 2022;53:2730-2738.

208. Ilic NV, Dubljanin-Raspopovic E, Nedeljkovic U et al. Effects of anodal tDCS and occupational therapy on fine motor skill deficits in patients with chronic stroke. Restor Neurol Neurosci 2016;34:935-945.

209. Inguaggiato E, Bolognini N, Fiori S, Cioni G. Transcranial Direct Current Stimulation (tDCS) in Unilateral Cerebral Palsy: A Pilot Study of Motor Effect. Neural Plast 2019;2019:2184398.

210. Iwanski S, Lesniak M, Polanowska K, Bembenek J, Czepiel W, Seniow J. Neuronavigated 1 Hz rTMS of the left angular gyrus combined with visuospatial therapy in post-stroke neglect. NeuroRehabilitation 2020;46:83-93.

211. Jayaraman A, O'Brien MK, Madhavan S et al. Stride management assist exoskeleton vs functional gait training in stroke: A randomized trial. Neurology 2019;92:e263-e273.

212. Jeon SY, Han SJ. Improvement of the working memory and naming by transcranial direct current stimulation. Ann Rehabil Med 2012;36:585-95.

213. Jiang W, Tang W, Song Y et al. Effectiveness of repetitive transcranial magnetic stimulation against poststroke urinary incontinence: a study protocol for a randomized controlled trial. Trials 2022;23:650.

214. Jiao Y, Peng W, Yang J, Li C. Effect of Repetitive Transcranial Magnetic Stimulation on the Nutritional Status and Neurological Function of Patients With Postischemic Stroke Dysphagia. Neurologist 2023;28:69-72.

215. Jin M, Zhang Z, Bai Z, Fong KNK. Timing-dependent interaction effects of tDCS with mirror therapy on upper extremity motor recovery in patients with chronic stroke: A randomized controlled pilot study. J Neurol Sci 2019;405:116436.

216. Johansson BB. Multisensory stimulation in stroke rehabilitation. Front Hum Neurosci 2012;6:60.

217. Johnstone A, Grigoras I, Petitet P, Capitao LP, Stagg CJ. A single, clinically relevant dose of the GABA(B) agonist baclofen impairs visuomotor learning. J Physiol 2021;599:307-322.

218. Juan D, Yao W, Li J et al. Motor Network Reorganization After Repetitive Transcranial Magnetic Stimulation in Early Stroke Patients: A Resting State fMRI Study. Neurorehabil Neural Repair 2022;36:61-68.

219. Kakuda W, Abo M, Sasanuma J et al. Combination Protocol of Low-Frequency rTMS and Intensive Occupational Therapy for Post-stroke Upper Limb Hemiparesis: a 6-year Experience of More Than 1700 Japanese Patients. Transl Stroke Res 2016;7:172-9.

220. Kakuda W, Abo M, Shimizu M et al. A multi-center study on low-frequency rTMS combined with intensive occupational therapy for upper limb hemiparesis in post-stroke patients. J Neuroeng Rehabil 2012;9:4.

221. Kang JH, Kim MW, Park KH, Choi YA. The effects of additional electrical stimulation combined with repetitive transcranial magnetic stimulation and motor imagery on upper extremity motor recovery in the subacute period after stroke: A preliminary study. Medicine (Baltimore) 2021;100:e27170.

222. Kasashima Y, Fujiwara T, Matsushika Y et al. Modulation of event-related desynchronization during motor imagery with transcranial direct current stimulation (tDCS) in patients with chronic hemiparetic stroke. Exp Brain Res 2012;221:263-8.

223. Kasashima-Shindo Y, Fujiwara T, Ushiba J et al. Brain-computer interface training combined with transcranial direct current stimulation in patients with chronic severe hemiparesis: Proof of concept study. J Rehabil Med 2015;47:318-24.

224. Kazuta T, Takeda K, Osu R et al. Transcranial Direct Current Stimulation Improves Audioverbal Memory in Stroke Patients. Am J Phys Med Rehabil 2017;96:565-571.

225. Keser Z, Dehgan MW, Shadravan S, Yozbatiran N, Maher LM, Francisco GE. Combined Dextroamphetamine and Transcranial Direct Current Stimulation in Poststroke Aphasia. Am J Phys Med Rehabil 2017;96:S141-S145.

226. Khedr EM, Abo El-Fetoh N, Ali AM et al. Dual-hemisphere repetitive transcranial magnetic stimulation for rehabilitation of poststroke aphasia: a randomized, double-blind clinical trial. Neurorehabil Neural Repair 2014;28:740-50.

227. Khedr EM, Shawky OA, El-Hammady DH et al. Effect of anodal versus cathodal transcranial direct current stimulation on stroke rehabilitation: a pilot randomized controlled trial. Neurorehabil Neural Repair 2013;27:592-601.

228. Kim BR, Chun MH, Kim DY, Lee SJ. Effect of high- and low-frequency repetitive transcranial magnetic stimulation on visuospatial neglect in patients with acute stroke: a double-blind, sham-controlled trial. Arch Phys Med Rehabil 2013;94:803-7.

229. Kim J, Yim J. Effects of High-Frequency Repetitive Transcranial Magnetic Stimulation Combined with Task-Oriented Mirror Therapy Training on Hand Rehabilitation of Acute Stroke Patients. Med Sci Monit 2018;24:743-750.

230. Kim JH, Han JY, Song MK, Park GC, Lee JS. Synergistic Effects of Scalp Acupuncture and Repetitive Transcranial Magnetic Stimulation on Cerebral Infarction: A Randomized Controlled Pilot Trial. Brain Sci 2020;10.

231. Kim JY, Boudier-Reveret M, Chang M. Can repetitive transcranial magnetic stimulation enhance motor outcomes in cerebral infarct patients? J Integr Neurosci 2020;19:119-123.

232. Kim SB, Lee KW, Lee JH, Lee SJ, Park JG, Lee JB. Effect of Combined Therapy of Robot and Low-Frequency Repetitive Transcranial Magnetic Stimulation on Hemispatial Neglect in Stroke Patients. Ann Rehabil Med 2018;42:788-797.

233. Kim SH. Effects of Dual Transcranial Direct Current Stimulation and Modified Constraint-Induced Movement Therapy to Improve Upper-Limb Function after Stroke: A Double-Blinded, Pilot Randomized Controlled Trial. J Stroke Cerebrovasc Dis 2021;30:105928.

234. Kim WS, Cho S, Park SH, Lee JY, Kwon S, Paik NJ. A low cost kinect-based virtual rehabilitation system for inpatient rehabilitation of the upper limb in patients with subacute stroke: A randomized, double-blind, sham-controlled pilot trial. Medicine (Baltimore) 2018;97:e11173.

235. Kim WS, Jung SH, Oh MK, Min YS, Lim JY, Paik NJ. Effect of repetitive transcranial magnetic stimulation over the cerebellum on patients with ataxia after posterior circulation stroke: A pilot study. J Rehabil Med 2014;46:418-23.

236. Kim WS, Kwon BS, Seo HG, Park J, Paik NJ. Low-Frequency Repetitive Transcranial Magnetic Stimulation Over Contralesional Motor Cortex for Motor Recovery in Subacute Ischemic Stroke: A Randomized Sham-Controlled Trial. Neurorehabil Neural Repair 2020;34:856-867.

237. Kim YK, Jung JH, Shin SH. A comparison of the effects of repetitive transcranial magnetic stimulation (rTMS) by number of stimulation sessions on hemispatial neglect in chronic stroke patients. Exp Brain Res 2015;233:283-9.

238. Kindler J, Schumacher R, Cazzoli D et al. Theta burst stimulation over the right Broca's homologue induces improvement of naming in aphasic patients. Stroke 2012;43:2175-9.

239. Kindred JH, Kautz SA, Wonsetler EC, Bowden MG. Single Sessions of High-Definition Transcranial Direct Current Stimulation Do Not Alter Lower Extremity Biomechanical or Corticomotor Response Variables Post-stroke. Front Neurosci 2019;13:286.

240. Kindred JH, Wonsetler EC, Charalambous CC et al. Individualized Responses to Ipsilesional High-Frequency and Contralesional Low-Frequency rTMS in Chronic Stroke: A Pilot Study to Support the Individualization of Neuromodulation for Rehabilitation. Front Hum Neurosci 2020;14:578127.

241. Kirton A, Andersen J, Herrero M et al. Brain stimulation and constraint for perinatal stroke hemiparesis: The PLASTIC CHAMPS Trial. Neurology 2016;86:1659-67.

242. Kirton A, Ciechanski P, Zewdie E et al. Transcranial direct current stimulation for children with perinatal stroke and hemiparesis. Neurology 2017;88:259-267.

243. Kleineberg NN, Richter MK, Becker I, Weiss PH, Fink GR. Verum versus sham tDCS in the treatment of stroke-induced apraxia: study protocol of the randomized controlled trial RAdiCS -"Rehabilitating (stroke-induced) Apraxia with direct Current Stimulation". Neurol Res Pract 2020;2:7.

244. Klomjai W, Aneksan B. A randomized sham-controlled trial on the effects of dual-tDCS "during" physical therapy on lower limb performance in sub-acute stroke and a comparison to the previous study using a "before" stimulation protocol. BMC Sports Sci Med Rehabil 2022;14:68.

245. Klomjai W, Aneksan B, Chotik-Anuchit S et al. Effects of Different Montages of Transcranial Direct Current Stimulation on Haemodynamic Responses and Motor Performance in Acute Stroke: A Randomized Controlled Trial. J Rehabil Med 2022;54:jrm00331.

246. Klomjai W, Aneksan B, Pheungphrarattanatrai A et al. Effect of single-session dual-tDCS before physical therapy on lower-limb performance in sub-acute stroke patients: A randomized sham-controlled crossover study. Ann Phys Rehabil Med 2018;61:286-291.

247. Klomjai W, Giron A, Mounir El Mendili M et al. Anodal tDCS of contralesional hemisphere modulates ipsilateral control of spinal motor networks targeting the paretic arm post-stroke. Clin Neurophysiol 2022;136:1-12.

248. Ko MH, Yoon JY, Jo YJ et al. Home-Based Transcranial Direct Current Stimulation to Enhance Cognition in Stroke: Randomized Controlled Trial. Stroke 2022;53:2992-3001.

249. Koch G, Bonni S, Casula EP et al. Effect of Cerebellar Stimulation on Gait and Balance Recovery in Patients With Hemiparetic Stroke: A Randomized Clinical Trial. JAMA Neurol 2019;76:170-178.

250. Koch G, Bonni S, Giacobbe V et al. theta-burst stimulation of the left hemisphere accelerates recovery of hemispatial neglect. Neurology 2012;78:24-30.

251. Koh CL, Lin JH, Jeng JS, Huang SL, Hsieh CL. Effects of Transcranial Direct Current Stimulation With Sensory Modulation on Stroke Motor Rehabilitation: A Randomized Controlled Trial. Arch Phys Med Rehabil 2017;98:2477-2484.

252. Kolskar KK, Richard G, Alnaes D et al. Reliability, sensitivity, and predictive value of fMRI during multiple object tracking as a marker of cognitive training gain in combination with tDCS in stroke survivors. Hum Brain Mapp 2021;42:1167-1181.

253. Koo WR, Jang BH, Kim CR. Effects of Anodal Transcranial Direct Current Stimulation on Somatosensory Recovery After Stroke: A Randomized Controlled Trial. Am J Phys Med Rehabil 2018;97:507-513.

254. Kulishova TV, Shinkorenko OV. [The effectiveness of early rehabilitation of the patients presenting with ischemic stroke]. Vopr Kurortol Fizioter Lech Fiz Kult 2014:9-12.

255. Kulishova TV, Shinkorenko OV. [The efficacy of transcranial magnetic stimulation in acute ischemic stroke]. Zh Nevrol Psikhiatr Im S S Korsakova 2015;115:67-72.

256. Kumar S, Marchina S, Langmore S et al. Fostering eating after stroke (FEASt) trial for improving post-stroke dysphagia with non-invasive brain stimulation. Sci Rep 2022;12:9607.

257. Kumari N, Taylor D, Olsen S, Rashid U, Signal N. Cerebellar Transcranial Direct Current Stimulation for Motor Learning in People with Chronic Stroke: A Pilot Randomized Controlled Trial. Brain Sci 2020;10.

258. Kuo HC, Litzenberger J, Nettel-Aguirre A, Zewdie E, Kirton A. Exploring Clinical and Neurophysiological Factors Associated with Response to Constraint Therapy and Brain Stimulation in Children with Hemiparetic Cerebral Palsy. Dev Neurorehabil 2022;25:229-238.

259. Kuo HC, Zewdie E, Ciechanski P, Damji O, Kirton A. Intervention-Induced Motor Cortex Plasticity in Hemiparetic Children With Perinatal Stroke. Neurorehabil Neural Repair 2018;32:941-952.

260. Kuo IJ, Tang CW, Tsai YA et al. Neurophysiological signatures of hand motor response to dual-transcranial direct current stimulation in subacute stroke: a TMS and MEG study. J Neuroeng Rehabil 2020;17:72.

261. Kuzu O, Adiguzel E, Kesikburun S, Yasar E, Yilmaz B. The Effect of Sham Controlled Continuous Theta Burst Stimulation and Low Frequency Repetitive Transcranial Magnetic Stimulation on Upper Extremity Spasticity and Functional Recovery in Chronic Ischemic Stroke Patients. J Stroke Cerebrovasc Dis 2021;30:105795.

262. Kwon TG, Kim YH, Chang WH, Bang OY, Shin YI. Effective method of combining rTMS and motor training in stroke patients. Restor Neurol Neurosci 2014;32:223-32.

263. Kwon TG, Park E, Kang C, Chang WH, Kim YH. The effects of combined repetitive transcranial magnetic stimulation and transcranial direct current stimulation on motor function in patients with stroke. Restor Neurol Neurosci 2016;34:915-923.

264. Kwon YG, Do KH, Park SJ, Chang MC, Chun MH. Effect of Repetitive Transcranial Magnetic Stimulation on Patients With Dysarthria After Subacute Stroke. Ann Rehabil Med 2015;39:793-9.

265. Ladavas E, Giulietti S, Avenanti A et al. a-tDCS on the ipsilesional parietal cortex boosts the effects of prism adaptation treatment in neglect. Restor Neurol Neurosci 2015;33:647-62.

266. Lappchen CH, Ringer T, Blessin J et al. Daily iTBS worsens hand motor training--a combined TMS, fMRI and mirror training study. Neuroimage 2015;107:257-265.

267. Learmonth G, Benwell CSY, Marker G et al. Non-invasive brain stimulation in Stroke patients (NIBS): A prospective randomized open blinded end-point (PROBE) feasibility trial using transcranial direct current stimulation (tDCS) in post-stroke hemispatial neglect. Neuropsychol Rehabil 2021;31:1163-1189.

268. Lee DJ, Lee YS, Kim HJ, Seo TH. The effects of exercise training using transcranial direct current stimulation (tDCS) on breathing in patients with chronic stroke patients. J Phys Ther Sci 2017;29:527-530.

269. Lee IT, Huang CC, Hsu PC, Lin CP, Tsai PY. Resting-State Network Changes Following Transcranial Magnetic Stimulation in Patients With Aphasia-A Randomized Controlled Study. Neuromodulation 2022;25:528-537.

270. Lee J, Park E, Lee A et al. Modulating Brain Connectivity by Simultaneous Dual-Mode Stimulation over Bilateral Primary Motor Cortices in Subacute Stroke Patients. Neural Plast 2018;2018:1458061.

271. Lee S, Cha H. The effect of clinical application of transcranial direct current stimulation combined with non-immersive virtual reality rehabilitation in stroke patients. Technol Health Care 2022;30:117-127.

272. Lee SH, Kim WS, Park J, Kim J, Paik NJ. Effects of anodal transcranial direct current stimulation over the contralesional hemisphere on motor recovery in subacute stroke patients with severe upper extremity hemiparesis: Study protocol for a randomized controlled trial. Medicine (Baltimore) 2020;99:e19495.

273. Lee SJ, Chun MH. Combination transcranial direct current stimulation and virtual reality therapy for upper extremity training in patients with subacute stroke. Arch Phys Med Rehabil 2014;95:431-8.

274. Lefebvre S, Dricot L, Laloux P et al. Increased functional connectivity one week after motor learning and tDCS in stroke patients. Neuroscience 2017;340:424-435.

275. Lefebvre S, Dricot L, Laloux P et al. Neural substrates underlying stimulation-enhanced motor skill learning after stroke. Brain 2015;138:149-63.

276. Lefebvre S, Laloux P, Peeters A, Desfontaines P, Jamart J, Vandermeeren Y. Dual-tDCS Enhances Online Motor Skill Learning and Long-Term Retention in Chronic Stroke Patients. Front Hum Neurosci 2012;6:343.

277. Lefebvre S, Thonnard JL, Laloux P, Peeters A, Jamart J, Vandermeeren Y. Single session of dual-tDCS transiently improves precision grip and dexterity of the paretic hand after stroke. Neurorehabil Neural Repair 2014;28:100-10.

278. Leistner S, Michelson G, Laumeier I et al. Intensified secondary prevention intending a reduction of recurrent events in TIA and minor stroke patients (INSPiRE-TMS): a protocol for a randomised controlled trial. BMC Neurol 2013;13:11.

279. Lempka SF, Malone DA, Jr., Hu B et al. Randomized clinical trial of deep brain stimulation for poststroke pain. Ann Neurol 2017;81:653-663.

280. Levin MF, Banina MC, Frenkel-Toledo S et al. Personalized upper limb training combined with anodal-tDCS for sensorimotor recovery in spastic hemiparesis: study protocol for a randomized controlled trial. Trials 2018;19:7.

281. Li C, Zhang N, Han Q et al. Prolonged Continuous Theta Burst Stimulation Can Regulate Sensitivity on Abeta Fibers: An Functional Near-Infrared Spectroscopy Study. Front Mol Neurosci 2022;15:887426.

282. Li F, Zhang T, Li BJ, Zhang W, Zhao J, Song LP. Motor imagery training induces changes in brain neural networks in stroke patients. Neural Regen Res 2018;13:1771-1781.

283. Li H, Ma J, Zhang J, Shi WY, Mei HN, Xing Y. Repetitive Transcranial Magnetic Stimulation (rTMS) Modulates Thyroid Hormones Level and Cognition in the Recovery Stage of Stroke Patients with Cognitive Dysfunction. Med Sci Monit 2021;27:e931914.

284. Li H, Zhu N, Klomparens EA et al. Application of functional near-infrared spectroscopy to explore the neural mechanism of transcranial direct current stimulation for post-stroke depression. Neurol Res 2019;41:714-721.

285. Li J, Meng XM, Li RY, Zhang R, Zhang Z, Du YF. Effects of different frequencies of repetitive transcranial magnetic stimulation on the recovery of upper limb motor dysfunction in patients with subacute cerebral infarction. Neural Regen Res 2016;11:1584-1590.

286. Li J, Wang H, Yuan Y et al. Effects of high frequency rTMS of contralesional dorsal premotor cortex in severe subcortical chronic stroke: protocol of a randomized controlled trial with multimodal neuroimaging assessments. BMC Neurol 2022;22:125.

287. Li J, Zuo Z, Zhang X et al. Excitatory Repetitive Transcranial Magnetic Stimulation Induces Contralesional Cortico-Cerebellar Pathways After Acute Ischemic Stroke: A Preliminary DTI Study. Front Behav Neurosci 2018;12:160.

288. Li LX, Lu JK, Li BJ et al. The optimum parameters and neuroimaging mechanism of repetitive transcranial magnetic stimulation to post-stroke cognitive impairment, a protocol of an orthogonally-designed randomized controlled trial. PLoS One 2022;17:e0271283.

289. Li X, Lin YL, Cunningham DA et al. Repetitive Transcranial Magnetic Stimulation of the Contralesional Dorsal Premotor Cortex for Upper Extremity Motor Improvement in Severe Stroke: Study Protocol for a Pilot Randomized Clinical Trial. Cerebrovasc Dis 2022;51:557-564.

290. Li Y, Feng H, Li J, Wang H, Chen N, Yang J. The effect of transcranial direct current stimulation of pharyngeal motor cortex on swallowing function in patients with chronic dysphagia after stroke: A retrospective cohort study. Medicine (Baltimore) 2020;99:e19121.

291. Li Y, Li K, Feng R et al. Mechanisms of Repetitive Transcranial Magnetic Stimulation on Post-stroke Depression: A Resting-State Functional Magnetic Resonance Imaging Study. Brain Topogr 2022;35:363-374.

292. Li Y, Luo H, Yu Q et al. Cerebral Functional Manipulation of Repetitive Transcranial Magnetic Stimulation in Cognitive Impairment Patients After Stroke: An fMRI Study. Front Neurol 2020;11:977.

293. Liao LY, Xie YJ, Chen Y, Gao Q. Cerebellar Theta-Burst Stimulation Combined With Physiotherapy in Subacute and Chronic Stroke Patients: A Pilot Randomized Controlled Trial. Neurorehabil Neural Repair 2021;35:23-32.

294. Liao WW, Chiang WC, Lin KC et al. Timing-dependent effects of transcranial direct current stimulation with mirror therapy on daily function and motor control in chronic stroke: a randomized controlled pilot study. J Neuroeng Rehabil 2020;17:101.

295. Lim H, Madhavan S. Differential corticomotor mechanisms of ankle motor control in post stroke individuals with and without motor evoked potentials. Brain Res 2020;1739:146833.

296. Lim KB, Lee HJ, Yoo J, Kwon YG. Effect of Low-Frequency rTMS and NMES on Subacute Unilateral Hemispheric Stroke With Dysphagia. Ann Rehabil Med 2014;38:592-602.

297. Lin BF, Yeh SC, Kao YJ, Lu CF, Tsai PY. Functional Remodeling Associated With Language Recovery After Repetitive Transcranial Magnetic Stimulation in Chronic Aphasic Stroke. Front Neurol 2022;13:809843.

298. Lin LF, Chang KH, Huang YZ, Lai CH, Liou TH, Lin YN. Simultaneous stimulation in bilateral leg motor areas with intermittent theta burst stimulation to improve functional performance after stroke: a feasibility pilot study. Eur J Phys Rehabil Med 2019;55:162-168.

299. Lin WS, Chou CL, Chang MH, Chung YM, Lin FG, Tsai PY. Vagus nerve magnetic modulation facilitates dysphagia recovery in patients with stroke involving the brainstem - A proof of concept study. Brain Stimul 2018;11:264-270.

300. Lin YN, Hu CJ, Chi JY et al. Effects of repetitive transcranial magnetic stimulation of the unaffected hemisphere leg motor area in patients with subacute stroke and substantial leg impairment: A pilot study. J Rehabil Med 2015;47:305-10.

301. Lindenberg R, Nachtigall L, Meinzer M, Sieg MM, Floel A. Differential effects of dual and unihemispheric motor cortex stimulation in older adults. J Neurosci 2013;33:9176-83.

302. Lindenberg R, Zhu LL, Ruber T, Schlaug G. Predicting functional motor potential in chronic stroke patients using diffusion tensor imaging. Hum Brain Mapp 2012;33:1040-51.

303. Lindenberg R, Zhu LL, Schlaug G. Combined central and peripheral stimulation to facilitate motor recovery after stroke: the effect of number of sessions on outcome. Neurorehabil Neural Repair 2012;26:479-83.

304. Liu H, Au-Yeung SSY. Corticomotor Excitability Effects of Peripheral Nerve Electrical Stimulation to the Paretic Arm in Stroke. Am J Phys Med Rehabil 2017;96:687-693.

305. Liu H, Peng Y, Liu Z et al. Hemodynamic signal changes and swallowing improvement of repetitive transcranial magnetic stimulation on stroke patients with dysphagia: A randomized controlled study. Front Neurol 2022;13:918974.

306. Liu J, Zhang J, Wang LN. Gamma aminobutyric acid (GABA) receptor agonists for acute stroke. Cochrane Database Syst Rev 2018;10:CD009622.

307. Liu S, Wang X, Yu R, Sun Y. Effect of transcranial magnetic stimulation on treatment effect and immune function. Saudi J Biol Sci 2022;29:379-384.

308. Liu Y, Yin M, Luo J et al. Effects of transcranial magnetic stimulation on the performance of the activities of daily living and attention function after stroke: a randomized controlled trial. Clin Rehabil 2020;34:1465-1473.

309. Liu YW, Chen ZH, Luo J et al. Explore combined use of transcranial direct current stimulation and cognitive training on executive function after stroke. J Rehabil Med 2021;53:jrm00162.

310. Llorens R, Fuentes MA, Borrego A et al. Effectiveness of a combined transcranial direct current stimulation and virtual reality-based intervention on upper limb function in chronic individuals post-stroke with persistent severe hemiparesis: a randomized controlled trial. J Neuroeng Rehabil 2021;18:108.

311. Long H, Wang H, Zhao C et al. Effects of combining high- and low-frequency repetitive transcranial magnetic stimulation on upper limb hemiparesis in the early phase of stroke. Restor Neurol Neurosci 2018;36:21-30.

312. Lopez-Romero LA, Riano-Carreno DM, Pachon-Poveda MY et al. [Efficacy and safety of transcranial magnetic stimulation in patients with non-fluent aphasia, following an ischaemic stroke. A controlled, randomised and double-blind clinical trial]. Rev Neurol 2019;68:241-249.

313. Lu H, Zhang T, Wen M, Sun L. Impact of repetitive transcranial magnetic stimulation on post-stroke dysmnesia and the role of BDNF Val66Met SNP. Med Sci Monit 2015;21:761-8.

314. Lu Y, Zhou W, Lin Y, Du Y, Zhang X. The effects of traditional Chinese medicine sensory stimulation combined with transcranial direct current stimulation on deglutition and related complications in stroke patients with dysphagia: a randomized trial. Ann Palliat Med 2021;10:6597-6605.

315. Ludemann-Podubecka J, Bosl K, Nowak DA. Inhibition of the contralesional dorsal premotor cortex improves motor function of the affected hand following stroke. Eur J Neurol 2016;23:823-30.

316. Ludemann-Podubecka J, Bosl K, Theilig S, Wiederer R, Nowak DA. The Effectiveness of 1 Hz rTMS Over the Primary Motor Area of the Unaffected Hemisphere to Improve Hand Function After Stroke Depends on Hemispheric Dominance. Brain Stimul 2015;8:823-30.

317. Luk KY, Ouyang HX, Pang MYC. Low-Frequency rTMS over Contralesional M1 Increases Ipsilesional Cortical Excitability and Motor Function with Decreased Interhemispheric Asymmetry in Subacute Stroke: A Randomized Controlled Study. Neural Plast 2022;2022:3815357.

318. Luvizutto GJ, Rizzati GR, Fogaroli MO et al. Treatment of unilateral spatial neglect after stroke using transcranial direct current stimulation (ELETRON trial): study protocol for a randomized controlled trial. Trials 2016;17:479.

319. Madhavan S, Cleland BT, Sivaramakrishnan A et al. Cortical priming strategies for gait training after stroke: a controlled, stratified trial. J Neuroeng Rehabil 2020;17:111.

320. Malcolm MP, Enney L, Cramer SC. Methods for an International Randomized Clinical Trial to Investigate the Effect of Gsk249320 on Motor Cortex Neurophysiology using Transcranial Magnetic Stimulation in Survivors of Stroke. J Clin Trials 2014;4:1-9.

321. Malcolm MP, Vaughn HN, Greene DP. Inhibitory and excitatory motor cortex dysfunction persists in the chronic poststroke recovery phase. J Clin Neurophysiol 2015;32:251-6.

322. Mane R, Chew E, Phua KS et al. Prognostic and Monitory EEG-Biomarkers for BCI Upper-Limb Stroke Rehabilitation. IEEE Trans Neural Syst Rehabil Eng 2019;27:1654-1664.

323. Manji A, Amimoto K, Matsuda T, Wada Y, Inaba A, Ko S. Effects of transcranial direct current stimulation over the supplementary motor area body weight-supported treadmill gait training in hemiparetic patients after stroke. Neurosci Lett 2018;662:302-305.

324. Mao H, Lyu Y, Li Y et al. Clinical study on swallowing function of brainstem stroke by tDCS. Neurol Sci 2022;43:477-484.

325. Marangolo P, Fiori V, Caltagirone C, Pisano F, Priori A. Transcranial Cerebellar Direct Current Stimulation Enhances Verb Generation but Not Verb Naming in Poststroke Aphasia. J Cogn Neurosci 2018;30:188-199.

326. Marchina S, Schlaug G, Kumar S. Study design for the fostering eating after stroke with transcranial direct current stimulation trial: a randomized controlled intervention for improving Dysphagia after acute ischemic stroke. J Stroke Cerebrovasc Dis 2015;24:511-20.

327. Marquez JL, Conley AC, Karayanidis F, Miller J, Lagopoulos J, Parsons MW. Determining the benefits of transcranial direct current stimulation on functional upper limb movement in chronic stroke. Int J Rehabil Res 2017;40:138-145.

328. Martens G, Fregni F, Carriere M, Barra A, Laureys S, Thibaut A. Single tDCS session of motor cortex in patients with disorders of consciousness: a pilot study. Brain Inj 2019;33:1679-1683.

329. Massie CL, Malcolm MP. Considerations for stimulus-response curves in stroke: an investigation comparing collection and analysis methods. Int J Neurosci 2013;123:175-83.

330. Massie CL, Tracy BL, Malcolm MP. Functional repetitive transcranial magnetic stimulation increases motor cortex excitability in survivors of stroke. Clin Neurophysiol 2013;124:371-8.

331. Massie CL, Tracy BL, Paxton RJ, Malcolm MP. Repeated sessions of functional repetitive transcranial magnetic stimulation increases motor cortex excitability and motor control in survivors of stroke. NeuroRehabilitation 2013;33:185-93.

332. Matar SJ, Newton C, Sorinola IO, Pavlou M. Transcranial Direct-Current Stimulation as an Adjunct to Verb Network Strengthening Treatment in Post-stroke Chronic Aphasia: A Double-Blinded Randomized Feasibility Study. Front Neurol 2022;13:722402.

333. Matsuura A, Onoda K, Oguro H, Yamaguchi S. Magnetic stimulation and movement-related cortical activity for acute stroke with hemiparesis. Eur J Neurol 2015;22:1526-32.

334. Mazzoleni S, Tran VD, Dario P, Posteraro F. Effects of Transcranial Direct Current Stimulation (tDCS) Combined With Wrist Robot-Assisted Rehabilitation on Motor Recovery in Subacute Stroke Patients: A Randomized Controlled Trial. IEEE Trans Neural Syst Rehabil Eng 2019;27:1458-1466.

335. Mazzoleni S, Tran VD, Iardella L, Dario P, Posteraro F. Randomized, sham-controlled trial based on transcranial direct current stimulation and wrist robot-assisted integrated treatment on subacute stroke patients: Intermediate results. IEEE Int Conf Rehabil Robot 2017;2017:555-560.

336. McCambridge AB, Stinear JW, Byblow WD. A dissociation between propriospinal facilitation and inhibition after bilateral transcranial direct current stimulation. J Neurophysiol 2014;111:2187-95.

337. McCambridge AB, Stinear JW, Byblow WD. Revisiting interhemispheric imbalance in chronic stroke: A tDCS study. Clin Neurophysiol 2018;129:42-50.

338. Meinzer M, Darkow R, Lindenberg R, Floel A. Electrical stimulation of the motor cortex enhances treatment outcome in post-stroke aphasia. Brain 2016;139:1152-63.

339. Meinzer M, Jahnigen S, Copland DA et al. Transcranial direct current stimulation over multiple days improves learning and maintenance of a novel vocabulary. Cortex 2014;50:137-47.

340. Mello EA, Cohen LG, Monteiro Dos Anjos S et al. Increase in Short-Interval Intracortical Facilitation of the Motor Cortex after Low-Frequency Repetitive Magnetic Stimulation of the Unaffected Hemisphere in the Subacute Phase after Stroke. Neural Plast 2015;2015:407320.

341. Menezes IS, Cohen LG, Mello EA et al. Combined Brain and Peripheral Nerve Stimulation in Chronic Stroke Patients With Moderate to Severe Motor Impairment. Neuromodulation 2018;21:176-183.

342. Meng L, Tsang RCC, Ge Y, Guo Q, Gao Q. rTMS for poststroke pusher syndrome: study protocol for a randomised, patient-blinded controlled clinical trial. BMJ Open 2022;12:e064905.

343. Meng Y, Zhang D, Hai H, Zhao YY, Ma YW. Efficacy of coupling intermittent theta-burst stimulation and 1 Hz repetitive transcranial magnetic stimulation to enhance upper limb motor recovery in subacute stroke patients: A randomized controlled trial. Restor Neurol Neurosci 2020;38:109-118.

344. Meng ZY, Song WQ. Low frequency repetitive transcranial magnetic stimulation improves motor dysfunction after cerebral infarction. Neural Regen Res 2017;12:610-613.

345. Metzler MJ, Haspels E, Brunton L et al. Goals of children with unilateral cerebral palsy in a brain stimulation arm rehabilitation trial. Dev Med Child Neurol 2021;63:584-591.

346. Michou E, Mistry S, Jefferson S, Singh S, Rothwell J, Hamdy S. Targeting unlesioned pharyngeal motor cortex improves swallowing in healthy individuals and after dysphagic stroke. Gastroenterology 2012;142:29-38.

347. Michou E, Mistry S, Jefferson S, Tyrrell P, Hamdy S. Characterizing the mechanisms of central and peripheral forms of neurostimulation in chronic dysphagic stroke patients. Brain Stimul 2014;7:66-73.

348. Michou E, Mistry S, Rothwell J, Hamdy S. Priming pharyngeal motor cortex by repeated paired associative stimulation: implications for dysphagia neurorehabilitation. Neurorehabil Neural Repair 2013;27:355-62.

349. Milot MH, Palimeris S, Corriveau H, Tremblay F, Boudrias MH. Effects of a tailored strength training program of the upper limb combined with transcranial direct current stimulation (tDCS) in chronic stroke patients: study protocol for a randomised, double-blind, controlled trial. BMC Sports Sci Med Rehabil 2019;11:8.

350. Mirowska-Guzel D, Gromadzka G, Seniow J et al. Association between BDNF-196 G>A and BDNF-270 C>T polymorphisms, BDNF concentration, and rTMS-supported long-term rehabilitation outcome after ischemic stroke. NeuroRehabilitation 2013;32:573-82.

351. Mitsutake T, Sakamoto M, Nakazono H, Horikawa E. The Effects of Combining Transcranial Direct Current Stimulation and Gait Training with Functional Electrical Stimulation on Trunk Acceleration During Walking in Patients with Subacute Stroke. J Stroke Cerebrovasc Dis 2021;30:105635.

352. Miyai I, Mihara M, Hattori N, Hatakenaka M, Kawano T, Yagura H. [Contribution of brain function analysis to the evolution of neurorehabilitation]. Rinsho Shinkeigaku 2012;52:1174-7.

353. Mohan A, Knutson JS, Cunningham DA et al. Contralaterally Controlled Functional Electrical Stimulation Combined With Brain Stimulation for Severe Upper Limb Hemiplegia-Study Protocol for a Randomized Controlled Trial. Front Neurol 2022;13:869733.

354. Momosaki R, Abo M, Watanabe S, Kakuda W, Yamada N, Mochio K. Functional magnetic stimulation using a parabolic coil for dysphagia after stroke. Neuromodulation 2014;17:637-41; discussion 641.

355. Montenegro RA, Midgley A, Massaferri R, Bernardes W, Okano AH, Farinatti P. Bihemispheric Motor Cortex Transcranial Direct Current Stimulation Improves Force Steadiness in Post-Stroke Hemiparetic Patients: A Randomized Crossover Controlled Trial. Front Hum Neurosci 2016;10:426.

356. Moretti CB, Edwards DJ, Hamilton T et al. Robotic Kinematic measures of the arm in chronic Stroke: part 1 - Motor Recovery patterns from tDCS preceding intensive training. Bioelectron Med 2021;7:20.

357. Mortensen J, Figlewski K, Andersen H. Combined transcranial direct current stimulation and home-based occupational therapy for upper limb motor impairment following intracerebral hemorrhage: a double-blind randomized controlled trial. Disabil Rehabil 2016;38:637-43.

358. Moslemi Haghighi F, Kordi Yoosefinejad A, Razeghi M, Shariat A, Bagheri Z, Rezaei K. The Effect of High-Frequency Repetitive Transcranial Magnetic Stimulation on Functional Indices of Affected Upper Limb in Patients with Subacute Stroke. J Biomed Phys Eng 2021;11:175-184.

359. Motamed Vaziri P, Bahrpeyma F, Firoozabadi M et al. Low frequency repetitive transcranial magnetic stimulation to improve motor function and grip force of upper limbs of patients with hemiplegia. Iran Red Crescent Med J 2014;16:e13579.

360. Mrachacz-Kersting N, Stevenson AJT, Jorgensen HRM et al. Brain state-dependent stimulation boosts functional recovery following stroke. Ann Neurol 2019;85:84-95.

361. Muffel T, Shih PC, Kalloch B, Nikulin V, Villringer A, Sehm B. Differential effects of anodal and dual tDCS on sensorimotor functions in chronic hemiparetic stroke patients. Brain Stimul 2022;15:509-522.

362. Muller CO, Muthalib M, Mottet D et al. Recovering arm function in chronic stroke patients using combined anodal HD-tDCS and virtual reality therapy (ReArm): a study protocol for a randomized controlled trial. Trials 2021;22:747.

363. Murdoch K, Buckley JD, McDonnell MN. The Effect of Aerobic Exercise on Neuroplasticity within the Motor Cortex following Stroke. PLoS One 2016;11:e0152377.

364. Naeser MA, Martin PI, Ho M et al. Transcranial magnetic stimulation and aphasia rehabilitation. Arch Phys Med Rehabil 2012;93:S26-34.

365. Nepveu JF, Thiel A, Tang A et al. A Single Bout of High-Intensity Interval Training Improves Motor Skill Retention in Individuals With Stroke. Neurorehabil Neural Repair 2017;31:726-735.

366. Nguyen VT, Wu CW, Chen CA et al. Modulation of Interhemispheric Synchronization and Cortical Activity in Healthy Subjects by High-Definition Theta-Burst Electrical Stimulation. Neural Plast 2022;2022:3593262.

367. Nicolo P, Magnin C, Pedrazzini E et al. Comparison of Neuroplastic Responses to Cathodal Transcranial Direct Current Stimulation and Continuous Theta Burst Stimulation in Subacute Stroke. Arch Phys Med Rehabil 2018;99:862-872 e1.

368. Noh JS, Lim JH, Choi TW, Jang SG, Pyun SB. Effects and safety of combined rTMS and action observation for recovery of function in the upper extremities in stroke patients: A randomized controlled trial. Restor Neurol Neurosci 2019;37:219-230.

369. Nojima I, Mima T, Koganemaru S, Thabit MN, Fukuyama H, Kawamata T. Human motor plasticity induced by mirror visual feedback. J Neurosci 2012;32:1293-300.

370. Nyffeler T, Vanbellingen T, Kaufmann BC et al. Theta burst stimulation in neglect after stroke: functional outcome and response variability origins. Brain 2019;142:992-1008.

371. O'Neil-Pirozzi TM, Doruk D, Thomson JM, Fregni F. Immediate memory and electrophysiologic effects of prefrontal cortex transcranial direct current stimulation on neurotypical individuals and individuals with chronic traumatic brain injury: a pilot study. Int J Neurosci 2017;127:592-600.

372. Ochi M, Saeki S, Oda T, Matsushima Y, Hachisuka K. Effects of anodal and cathodal transcranial direct current stimulation combined with robotic therapy on severely affected arms in chronic stroke patients. J Rehabil Med 2013;45:137-40.

373. Olsen S, Signal N, Niazi IK et al. Peripheral Electrical Stimulation Paired With Movement-Related Cortical Potentials Improves Isometric Muscle Strength and Voluntary Activation Following Stroke. Front Hum Neurosci 2020;14:156.

374. Oveisgharan S, Organji H, Ghorbani A. Enhancement of Motor Recovery through Left Dorsolateral Prefrontal Cortex Stimulation after Acute Ischemic Stroke. J Stroke Cerebrovasc Dis 2018;27:185-191.

375. Palimeris S, Ansari Y, Remaud A et al. Effect of a tailored upper extremity strength training intervention combined with direct current stimulation in chronic stroke survivors: A Randomized Controlled Trial. Front Rehabil Sci 2022;3:978257.

376. Pan W, Wang P, Song X, Sun X, Xie Q. The Effects of Combined Low Frequency Repetitive Transcranial Magnetic Stimulation and Motor Imagery on Upper Extremity Motor Recovery Following Stroke. Front Neurol 2019;10:96.

377. Park E, Kim MS, Chang WH et al. Effects of Bilateral Repetitive Transcranial Magnetic Stimulation on Post-Stroke Dysphagia. Brain Stimul 2017;10:75-82.

378. Park HK, Song MK, Kim JH, Han JY. A randomized controlled trial to evaluate the effectiveness and safety of electro acupuncture and transcranial direct current stimulation with computerized cognitive rehabilitation in patients with vascular cognitive impairment. Medicine (Baltimore) 2020;99:e21263.

379. Park JW, Kim SB, Lee KW, Lee JH, Park JG, Lee SJ. Effects of Hand Training During the Aftereffect Period of Low-Frequency rTMS in Subacute Stroke Patients. Ann Rehabil Med 2018;42:521-527.

380. Park JW, Oh JC, Lee JW, Yeo JS, Ryu KH. The effect of 5Hz high-frequency rTMS over contralesional pharyngeal motor cortex in post-stroke oropharyngeal dysphagia: a randomized controlled study. Neurogastroenterol Motil 2013;25:324-e250.

381. Patel J, Fluet G, Qiu Q et al. Intensive virtual reality and robotic based upper limb training compared to usual care, and associated cortical reorganization, in the acute and early sub-acute periods post-stroke: a feasibility study. J Neuroeng Rehabil 2019;16:92.

382. Pestalozzi MI, Di Pietro M, Martins Gaytanidis C et al. Effects of Prefrontal Transcranial Direct Current Stimulation on Lexical Access in Chronic Poststroke Aphasia. Neurorehabil Neural Repair 2018;32:913-923.

383. Picelli A, Brugnera A, Filippetti M et al. Effects of two different protocols of cerebellar transcranial direct current stimulation combined with transcutaneous spinal direct current stimulation on robot-assisted gait training in patients with chronic supratentorial stroke: A single blind, randomized controlled trial. Restor Neurol Neurosci 2019;37:97-107.

384. Picelli A, Chemello E, Castellazzi P et al. Combined effects of cerebellar transcranial direct current stimulation and transcutaneous spinal direct current stimulation on robot-assisted gait training in patients with chronic brain stroke: A pilot, single blind, randomized controlled trial. Restor Neurol Neurosci 2018;36:161-171.

385. Picelli A, Chemello E, Castellazzi P et al. Combined effects of transcranial direct current stimulation (tDCS) and transcutaneous spinal direct current stimulation (tsDCS) on robot-assisted gait training in patients with chronic stroke: A pilot, double blind, randomized controlled trial. Restor Neurol Neurosci 2015;33:357-68.

386. Pilloni G, Vogel-Eyny A, Lustberg M et al. Tolerability and feasibility of at-home remotely supervised transcranial direct current stimulation (RS-tDCS): Single-center evidence from 6,779 sessions. Brain Stimul 2022;15:707-716.

387. Pingue V, Priori A, Malovini A, Pistarini C. Dual Transcranial Direct Current Stimulation for Poststroke Dysphagia: A Randomized Controlled Trial. Neurorehabil Neural Repair 2018;32:635-644.

388. Pinto AP, Guimaraes CL, Souza G et al. Sensory-motor and cardiorespiratory sensory rehabilitation associated with transcranial photobiomodulation in patients with central nervous system injury: Trial protocol for a single-center, randomized, double-blind, and controlled clinical trial. Medicine (Baltimore) 2019;98:e15851.

389. Pinto EF, Gupta A, Kulkarni GB, Andrade C. A Randomized, Double-Blind, Sham-Controlled Study of Transcranial Direct Current Stimulation as an Augmentation Intervention for the Attenuation of Motor Deficits in Patients With Stroke. J ECT 2021;37:281-290.

390. Pinto N, Goncalves H, Silva R, Duarte M, Gama J, Vaz Pato M. Theta burst stimulation over the prefrontal cortex: Effects on cerebral oximetry and cardiovascular measures in healthy humans. Neurosci Lett 2021;752:135792.

391. Pipatsrisawat S, Klaphajone J, Kitisak K, Sungkarat S, Wivatvongvana P. Effects of combining two techniques of non-invasive brain stimulation in subacute stroke patients: a pilot study. BMC Neurol 2022;22:98.

392. Pisano F, Caltagirone C, Incoccia C, Marangolo P. DUAL-tDCS Treatment over the Temporo-Parietal Cortex Enhances Writing Skills: First Evidence from Chronic Post-Stroke Aphasia. Life (Basel) 2021;11.

393. Pisano F, Caltagirone C, Incoccia C, Marangolo P. Spinal or cortical direct current stimulation: Which is the best? Evidence from apraxia of speech in post-stroke aphasia. Behav Brain Res 2021;399:113019.

394. Pisano F, Manfredini A, Castellano A, Caltagirone C, Marangolo P. Does Executive Function Training Impact on Communication? A Randomized Controlled tDCS Study on Post-Stroke Aphasia. Brain Sci 2022;12.

395. Pitts LL, Rogers L, Wang X, Bahia MM, Cherney LR. Functionally navigated transcranial magnetic stimulation to evoke lingual pressure in stroke survivors with dysphagia and healthy adults: a proof of concept trial. Top Stroke Rehabil 2020;27:241-250.

396. Plow EB, Cunningham DA, Beall E et al. Effectiveness and neural mechanisms associated with tDCS delivered to premotor cortex in stroke rehabilitation: study protocol for a randomized controlled trial. Trials 2013;14:331.

397. Plow EB, Cunningham DA, Varnerin N, Machado A. Rethinking stimulation of the brain in stroke rehabilitation: why higher motor areas might be better alternatives for patients with greater impairments. Neuroscientist 2015;21:225-40.

398. Plow EB, Obretenova SN, Jackson ML, Merabet LB. Temporal profile of functional visual rehabilitative outcomes modulated by transcranial direct current stimulation. Neuromodulation 2012;15:367-73.

399. Polanowska KE, Lesniak M, Seniow JB, Czlonkowska A. No effects of anodal transcranial direct stimulation on language abilities in early rehabilitation of post-stroke aphasic patients. Neurol Neurochir Pol 2013;47:414-22.

400. Polanowska KE, Lesniak MM, Seniow JB, Czepiel W, Czlonkowska A. Anodal transcranial direct current stimulation in early rehabilitation of patients with post-stroke non-fluent aphasia: a randomized, double-blind, sham-controlled pilot study. Restor Neurol Neurosci 2013;31:761-71.

401. Potter-Baker KA, Bonnett CE, Chabra P et al. A game of hide and seek: Is it possible to recruit more patients for NIBS studies in stroke? J Neurol Sci 2015;358:472-4.

402. Potter-Baker KA, Bonnett CE, Chabra P et al. Challenges in Recruitment for the Study of Noninvasive Brain Stimulation in Stroke: Lessons from Deep Brain Stimulation. J Stroke Cerebrovasc Dis 2016;25:927-37.

403. Powell ES, Carrico C, Raithatha R, Salyers E, Ward A, Sawaki L. Transvertebral direct current stimulation paired with locomotor training in chronic spinal cord injury: A case study. NeuroRehabilitation 2016;38:27-35.

404. Premi E, Cantoni V, Benussi A et al. Citicoline Treatment in Acute Ischemic Stroke: A Randomized, Single-Blind TMS Study. Front Neurol 2022;13:915362.

405. Pruvost-Robieux E, Benzakoun J, Turc G et al. Cathodal Transcranial Direct Current Stimulation in Acute Ischemic Stroke: Pilot Randomized Controlled Trial. Stroke 2021;52:1951-1960.

406. Pruvost-Robieux E, Calvet D, Ben Hassen W et al. Design and Methodology of a Pilot Randomized Controlled Trial of Transcranial Direct Current Stimulation in Acute Middle Cerebral Artery Stroke (STICA). Front Neurol 2018;9:816.

407. Pundik S, Skelly M, McCabe J, Akbari H, Tatsuoka C, Plow EB. Does rTMS Targeting Contralesional S1 Enhance Upper Limb Somatosensory Function in Chronic Stroke? A Proof-of-Principle Study. Neurorehabil Neural Repair 2021;35:233-246.

408. Qin Y, Liu X, Guo X, Liu M, Li H, Xu S. Low-Frequency Repetitive Transcranial Magnetic Stimulation Restores Dynamic Functional Connectivity in Subcortical Stroke. Front Neurol 2021;12:771034.

409. Rabadi MH, Aston CE. Effect of Transcranial Direct Current Stimulation on Severely Affected Arm-Hand Motor Function in Patients After an Acute Ischemic Stroke: A Pilot Randomized Control Trial. Am J Phys Med Rehabil 2017;96:S178-S184.

410. Ranjan S, Rezaee Z, Dutta A, Lahiri U. Feasibility of Cerebellar Transcranial Direct Current Stimulation to Facilitate Goal-Directed Weight Shifting in Chronic Post-Stroke Hemiplegics. IEEE Trans Neural Syst Rehabil Eng 2021;29:2203-2210.

411. Rao J, Li F, Zhong L et al. Bilateral Cerebellar Intermittent Theta Burst Stimulation Combined With Swallowing Speech Therapy for Dysphagia After Stroke: A Randomized, Double-Blind, Sham-Controlled, Clinical Trial. Neurorehabil Neural Repair 2022;36:437-448.

412. Rastgoo M, Naghdi S, Nakhostin Ansari N et al. Effects of repetitive transcranial magnetic stimulation on lower extremity spasticity and motor function in stroke patients. Disabil Rehabil 2016;38:1918-26.

413. Raty S, Borrmann C, Granata G et al. Non-invasive electrical brain stimulation for vision restoration after stroke: An exploratory randomized trial (REVIS). Restor Neurol Neurosci 2021;39:221-235.

414. Ren C, Zhang G, Xu X et al. The Effect of rTMS over the Different Targets on Language Recovery in Stroke Patients with Global Aphasia: A Randomized Sham-Controlled Study. Biomed Res Int 2019;2019:4589056.

415. Revill KP, Haut MW, Belagaje SR, Nahab F, Drake D, Buetefisch CM. Hebbian-Type Primary Motor Cortex Stimulation: A Potential Treatment of Impaired Hand Function in Chronic Stroke Patients. Neurorehabil Neural Repair 2020;34:159-171.

416. Rich T, Cassidy J, Menk J et al. Stability of stereognosis after pediatric repetitive transcranial magnetic stimulation and constraint-induced movement therapy clinical trial. Dev Neurorehabil 2017;20:169-172.

417. Rich TL, Menk J, Krach LE, Feyma T, Gillick BT. Repetitive Transcranial Magnetic Stimulation/Behavioral Intervention Clinical Trial: Long-Term Follow-Up of Outcomes in Congenital Hemiparesis. J Child Adolesc Psychopharmacol 2016;26:598-605.

418. Richardson J, Datta A, Dmochowski J, Parra LC, Fridriksson J. Feasibility of using high-definition transcranial direct current stimulation (HD-tDCS) to enhance treatment outcomes in persons with aphasia. NeuroRehabilitation 2015;36:115-26.

419. Rocha S, Silva E, Foerster A et al. The impact of transcranial direct current stimulation (tDCS) combined with modified constraint-induced movement therapy (mCIMT) on upper limb function in chronic stroke: a double-blind randomized controlled trial. Disabil Rehabil 2016;38:653-60.

420. Rodrigues L, Moncion K, Eng JJ et al. Intensity matters: protocol for a randomized controlled trial exercise intervention for individuals with chronic stroke. Trials 2022;23:442.

421. Rose DK, Patten C, McGuirk TE, Lu X, Triggs WJ. Does inhibitory repetitive transcranial magnetic stimulation augment functional task practice to improve arm recovery in chronic stroke? Stroke Res Treat 2014;2014:305236.

422. Rossano C, Terrier P. Visually-guided gait training in paretic patients during the first rehabilitation phase: study protocol for a randomized controlled trial. Trials 2016;17:523.

423. Rossi C, Sallustio F, Di Legge S, Stanzione P, Koch G. Transcranial direct current stimulation of the affected hemisphere does not accelerate recovery of acute stroke patients. Eur J Neurol 2013;20:202-4.

424. Rosso C, Moulton EJ, Kemlin C et al. Cerebello-Motor Paired Associative Stimulation and Motor Recovery in Stroke: a Randomized, Sham-Controlled, Double-Blind Pilot Trial. Neurotherapeutics 2022;19:491-500.

425. Rubi-Fessen I, Hartmann A, Huber W et al. Add-on Effects of Repetitive Transcranial Magnetic Stimulation on Subacute Aphasia Therapy: Enhanced Improvement of Functional Communication and Basic Linguistic Skills. A Randomized Controlled Study. Arch Phys Med Rehabil 2015;96:1935-44 e2.

426. Rungseethanakul S, Tretriluxana J, Piriyaprasarth P et al. Task Oriented Training Activities Post Stroke Will Produce Measurable Alterations in Brain Plasticity Concurrent with Skill Improvement. Top Stroke Rehabil 2022;29:241-254.

427. Saeys W, Vereeck L, Lafosse C, Truijen S, Wuyts FL, Van De Heyning P. Transcranial direct current stimulation in the recovery of postural control after stroke: a pilot study. Disabil Rehabil 2015;37:1857-63.

428. Saitoh Y, Hosomi K, Maruo T. [Stimulation of primary motor cortex and reorganization of cortical function]. Rinsho Shinkeigaku 2012;52:1182-4.

429. Salazar AP, Cimolin V, Schifino GP, Rech KD, Marchese RR, Pagnussat AS. Bi-cephalic transcranial direct current stimulation combined with functional electrical stimulation for upper-limb stroke rehabilitation: A double-blind randomized controlled trial. Ann Phys Rehabil Med 2020;63:4-11.

430. Sanches C, Levy R, Benisty S et al. Testing the therapeutic effects of transcranial direct current stimulation (tDCS) in semantic dementia: a double blind, sham controlled, randomized clinical trial. Trials 2019;20:632.

431. Sanchez-Cuesta FJ, Arroyo-Ferrer A, Gonzalez-Zamorano Y et al. Clinical Effects of Immersive Multimodal BCI-VR Training after Bilateral Neuromodulation with rTMS on Upper Limb Motor Recovery after Stroke. A Study Protocol for a Randomized Controlled Trial. Medicina (Kaunas) 2021;57.

432. Sankarasubramanian V, Machado AG, Conforto AB et al. Inhibition versus facilitation of contralesional motor cortices in stroke: Deriving a model to tailor brain stimulation. Clin Neurophysiol 2017;128:892-902.

433. Santos MD, Gagliardi RJ, Mac-Kay AP, Boggio PS, Lianza R, Fregni F. Transcranial direct-current stimulation induced in stroke patients with aphasia: a prospective experimental cohort study. Sao Paulo Med J 2013;131:422-6.

434. Santos MDD, Cavenaghi VB, Mac-Kay A et al. Non-invasive brain stimulation and computational models in post-stroke aphasic patients: single session of transcranial magnetic stimulation and transcranial direct current stimulation. A randomized clinical trial. Sao Paulo Med J 2017;135:475-480.

435. Santos-Pontelli TE, Rimoli BP, Favoretto DB et al. Polarity-Dependent Misperception of Subjective Visual Vertical during and after Transcranial Direct Current Stimulation (tDCS). PLoS One 2016;11:e0152331.

436. Sasaki N, Abo M, Hara T, Yamada N, Niimi M, Kakuda W. High-frequency rTMS on leg motor area in the early phase of stroke. Acta Neurol Belg 2017;117:189-194.

437. Sasaki N, Hara T, Yamada N, Niimi M, Kakuda W, Abo M. The Efficacy of High-Frequency Repetitive Transcranial Magnetic Stimulation for Improving Apathy in Chronic Stroke Patients. Eur Neurol 2017;78:28-32.

438. Sasaki N, Kakuda W, Abo M. Bilateral high- and low-frequency rTMS in acute stroke patients with hemiparesis: a comparative study with unilateral high-frequency rTMS. Brain Inj 2014;28:1682-6.

439. Sasaki N, Mizutani S, Kakuda W, Abo M. Comparison of the effects of high- and low-frequency repetitive transcranial magnetic stimulation on upper limb hemiparesis in the early phase of stroke. J Stroke Cerebrovasc Dis 2013;22:413-8.

440. Sasegbon A, Watanabe M, Simons A et al. Cerebellar repetitive transcranial magnetic stimulation restores pharyngeal brain activity and swallowing behaviour after disruption by a cortical virtual lesion. J Physiol 2019;597:2533-2546.

441. Sattler V, Acket B, Raposo N et al. Anodal tDCS Combined With Radial Nerve Stimulation Promotes Hand Motor Recovery in the Acute Phase After Ischemic Stroke. Neurorehabil Neural Repair 2015;29:743-54.

442. Schambra HM, Martinez-Hernandez IE, Slane KJ, Boehme AK, Marshall RS, Lazar RM. The neurophysiological effects of single-dose theophylline in patients with chronic stroke: A double-blind, placebo-controlled, randomized cross-over study. Restor Neurol Neurosci 2016;34:799-813.

443. Schuhmann T, Duecker F, Middag-van Spanje M et al. Transcranial alternating brain stimulation at alpha frequency reduces hemispatial neglect symptoms in stroke patients. Int J Clin Health Psychol 2022;22:100326.

444. Seamon BA, Bowden MG, Kindred JH, Embry AE, Kautz SA. Transcranial Direct Current Stimulation Electrode Montages May Differentially Impact Variables of Walking Performance in Individuals Poststroke: A Preliminary Study. J Clin Neurophysiol 2023;40:71-78.

445. Sebastian R, Kim JH, Brenowitz R et al. Cerebellar neuromodulation improves naming in post-stroke aphasia. Brain Commun 2020;2:fcaa179.

446. Sebastian R, Saxena S, Tsapkini K et al. Cerebellar tDCS: A Novel Approach to Augment Language Treatment Post-stroke. Front Hum Neurosci 2016;10:695.

447. Sehm B, Schafer A, Kipping J et al. Dynamic modulation of intrinsic functional connectivity by transcranial direct current stimulation. J Neurophysiol 2012;108:3253-63.

448. Seniow J, Bilik M, Lesniak M, Waldowski K, Iwanski S, Czlonkowska A. Transcranial magnetic stimulation combined with physiotherapy in rehabilitation of poststroke hemiparesis: a randomized, double-blind, placebo-controlled study. Neurorehabil Neural Repair 2012;26:1072-9.

449. Seniow J, Waldowski K, Lesniak M, Iwanski S, Czepiel W, Czlonkowska A. Transcranial magnetic stimulation combined with speech and language training in early aphasia rehabilitation: a randomized double-blind controlled pilot study. Top Stroke Rehabil 2013;20:250-61.

450. Seo HG, Lee WH, Lee SH, Yi Y, Kim KD, Oh BM. Robotic-assisted gait training combined with transcranial direct current stimulation in chronic stroke patients: A pilot double-blind, randomized controlled trial. Restor Neurol Neurosci 2017;35:527-536.

451. Shah-Basak PP, Norise C, Garcia G, Torres J, Faseyitan O, Hamilton RH. Individualized treatment with transcranial direct current stimulation in patients with chronic non-fluent aphasia due to stroke. Front Hum Neurosci 2015;9:201.

452. Shaheiwola N, Zhang B, Jia J, Zhang D. Using tDCS as an Add-On Treatment Prior to FES Therapy in Improving Upper Limb Function in Severe Chronic Stroke Patients: A Randomized Controlled Study. Front Hum Neurosci 2018;12:233.

453. Shaker HA, Sawan SAE, Fahmy EM, Ismail RS, Elrahman S. Effect of transcranial direct current stimulation on cognitive function in stroke patients. Egypt J Neurol Psychiatr Neurosurg 2018;54:32.

454. Shao Y, Yang Y, Sun YX, Xu AH. Different frequencies of repetitive transcranial magnetic stimulation combined with local injection of botulinum toxin type A for post-stroke lower limb spasticity: study protocol for a prospective, single-center, non-randomized, controlled clinical trial. Neural Regen Res 2022;17:2491-2496.

455. Sharma H, Vishnu VY, Kumar N et al. Efficacy of Low-Frequency Repetitive Transcranial Magnetic Stimulation in Ischemic Stroke: A Double-Blind Randomized Controlled Trial. Arch Rehabil Res Clin Transl 2020;2:100039.

456. Sharma R, Aranha VP, Saxena A, Samuel AJ. Effects of Dual Transcranial Direct Current Stimulation and Modified Constraint Induced Movement Therapy to Improve Upper-Limb Function After Stroke: A Double-Blinded, Pilot Randomized Controlled Trial. J Stroke Cerebrovasc Dis 2022;31:106227.

457. Shibata T, Urata A, Kawahara K et al. Therapeutic Effects of Diagonal-Transcranial Direct Current Stimulation on Functional Recovery in Acute Stroke: A Pilot Study. J Stroke Cerebrovasc Dis 2020;29:105107.

458. Shigematsu T, Fujishima I, Ohno K. Transcranial direct current stimulation improves swallowing function in stroke patients. Neurorehabil Neural Repair 2013;27:363-9.

459. Siddique U, Rahman S, Frazer A, Leung M, Pearce AJ, Kidgell DJ. Task-dependent modulation of corticospinal excitability and inhibition following strength training. J Electromyogr Kinesiol 2020;52:102411.

460. Silva FRD, Mac-Kay A, Chao JC, Santos MDD, Gagliadi RJ. Transcranial direct current stimulation: a study on naming performance in aphasic individuals. Codas 2018;30:e20170242.

461. Singer BJ, Vallence AM, Cleary S, Cooper I, Loftus AM. The effect of EMG triggered electrical stimulation plus task practice on arm function in chronic stroke patients with moderate-severe arm deficits. Restor Neurol Neurosci 2013;31:681-91.

462. Singh N, Saini M, Kumar N, Srivastava MVP, Mehndiratta A. Evidence of neuroplasticity with robotic hand exoskeleton for post-stroke rehabilitation: a randomized controlled trial. J Neuroeng Rehabil 2021;18:76.

463. Sivaramakrishnan A, Madhavan S. Absence of a Transcranial Magnetic Stimulation-Induced Lower Limb Corticomotor Response Does Not Affect Walking Speed in Chronic Stroke Survivors. Stroke 2018;49:2004-2007.

464. Sivaramakrishnan A, Madhavan S. Combining transcranial direct current stimulation with aerobic exercise to optimize cortical priming in stroke. Appl Physiol Nutr Metab 2021;46:426-435.

465. Soliman RK, Tax CMW, Abo-Elfetoh N et al. Effects of tDCS on Language Recovery in Post-Stroke Aphasia: A Pilot Study Investigating Clinical Parameters and White Matter Change with Diffusion Imaging. Brain Sci 2021;11.

466. Spielmann K, van de Sandt-Koenderman WM, Heijenbrok-Kal MH, Ribbers GM. Transcranial direct current stimulation in post-stroke sub-acute aphasia: study protocol for a randomized controlled trial. Trials 2016;17:380.

467. Spielmann K, van de Sandt-Koenderman WM, Heijenbrok-Kal MH, Ribbers GM. Comparison of two configurations of transcranial direct current stimulation for aphasia treatment. J Rehabil Med 2018;50:527-533.

468. Spielmann K, van de Sandt-Koenderman WME, Heijenbrok-Kal MH, Ribbers GM. Transcranial Direct Current Stimulation Does Not Improve Language Outcome in Subacute Poststroke Aphasia. Stroke 2018;49:1018-1020.

469. Stahl B, Darkow R, von Podewils V et al. Transcranial Direct Current Stimulation to Enhance Training Effectiveness in Chronic Post-Stroke Aphasia: A Randomized Controlled Trial Protocol. Front Neurol 2019;10:1089.

470. Stinear CM, Petoe MA, Anwar S, Barber PA, Byblow WD. Bilateral priming accelerates recovery of upper limb function after stroke: a randomized controlled trial. Stroke 2014;45:205-10.

471. Stoykov ME, Biller OM, Wax A et al. Bilateral upper extremity motor priming (BUMP) plus task-specific training for severe, chronic upper limb hemiparesis: study protocol for a randomized clinical trial. Trials 2022;23:523.

472. Stoykov ME, King E, David FJ, Vatinno A, Fogg L, Corcos DM. Bilateral motor priming for post stroke upper extremity hemiparesis: A randomized pilot study. Restor Neurol Neurosci 2020;38:11-22.

473. Straudi S, Baroni A, Mele S et al. Effects of a Robot-Assisted Arm Training Plus Hand Functional Electrical Stimulation on Recovery After Stroke: A Randomized Clinical Trial. Arch Phys Med Rehabil 2020;101:309-316.

474. Straudi S, Fregni F, Martinuzzi C, Pavarelli C, Salvioli S, Basaglia N. tDCS and Robotics on Upper Limb Stroke Rehabilitation: Effect Modification by Stroke Duration and Type of Stroke. Biomed Res Int 2016;2016:5068127.

475. Sun TT, Zhu GY, Zheng Y et al. Effects of paired associative magnetic stimulation between nerve root and cortex on motor function of lower limbs after spinal cord injury: study protocol for a randomized controlled trial. Neural Regen Res 2022;17:2459-2464.

476. Sun X, Dong X, Yuan Q et al. Effects of transcranial direct current stimulation on patients with post-stroke fatigue: a study protocol for a double-blind randomized controlled trial. Trials 2022;23:200.

477. Sung WH, Wang CP, Chou CL, Chen YC, Chang YC, Tsai PY. Efficacy of coupling inhibitory and facilitatory repetitive transcranial magnetic stimulation to enhance motor recovery in hemiplegic stroke patients. Stroke 2013;44:1375-82.

478. Suntrup-Krueger S, Ringmaier C, Muhle P et al. Randomized trial of transcranial direct current stimulation for poststroke dysphagia. Ann Neurol 2018;83:328-340.

479. Sunwoo H, Kim YH, Chang WH, Noh S, Kim EJ, Ko MH. Effects of dual transcranial direct current stimulation on post-stroke unilateral visuospatial neglect. Neurosci Lett 2013;554:94-8.

480. Tahtis V, Kaski D, Seemungal BM. The effect of single session bi-cephalic transcranial direct current stimulation on gait performance in sub-acute stroke: A pilot study. Restor Neurol Neurosci 2014;32:527-32.

481. Tai I, Lai CL, Hsu MJ et al. Effect of thermal stimulation on corticomotor excitability in patients with stroke. Am J Phys Med Rehabil 2014;93:801-8.

482. Takebayashi T, Takahashi K, Moriwaki M, Sakamoto T, Domen K. Improvement of Upper Extremity Deficit after Constraint-Induced Movement Therapy Combined with and without Preconditioning Stimulation Using Dual-hemisphere Transcranial Direct Current Stimulation and Peripheral Neuromuscular Stimulation in Chronic Stroke Patients: A Pilot Randomized Controlled Trial. Front Neurol 2017;8:568.

483. Takeuchi N, Tada T, Matsuo Y, Ikoma K. Low-frequency repetitive TMS plus anodal transcranial DCS prevents transient decline in bimanual movement induced by contralesional inhibitory rTMS after stroke. Neurorehabil Neural Repair 2012;26:988-98.

484. Talelli P, Wallace A, Dileone M et al. Theta burst stimulation in the rehabilitation of the upper limb: a semirandomized, placebo-controlled trial in chronic stroke patients. Neurorehabil Neural Repair 2012;26:976-87.

485. Tang Y, Chen A, Zhu S et al. Repetitive transcranial magnetic stimulation for depression after basal ganglia ischaemic stroke: protocol for a multicentre randomised double-blind placebo-controlled trial. BMJ Open 2018;8:e018011.

486. Tarameshlu M, Ansari NN, Ghelichi L, Jalaei S. The effect of repetitive transcranial magnetic stimulation combined with traditional dysphagia therapy on poststroke dysphagia: a pilot double-blinded randomized-controlled trial. Int J Rehabil Res 2019;42:133-138.

487. Tarri M, Brihmat N, Gasq D et al. Five-day course of paired associative stimulation fails to improve motor function in stroke patients. Ann Phys Rehabil Med 2018;61:78-84.

488. Taud B, Lindenberg R, Darkow R et al. Limited Add-On Effects of Unilateral and Bilateral Transcranial Direct Current Stimulation on Visuo-Motor Grip Force Tracking Task Training Outcome in Chronic Stroke. A Randomized Controlled Trial. Front Neurol 2021;12:736075.

489. Tedla JS, Rodrigues E, Ferreira AS et al. Transcranial direct current stimulation combined with trunk-targeted, proprioceptive neuromuscular facilitation in subacute stroke: a randomized controlled trial. PeerJ 2022;10:e13329.

490. Thiel A, Black SE, Rochon EA et al. Non-invasive repeated therapeutic stimulation for aphasia recovery: a multilingual, multicenter aphasia trial. J Stroke Cerebrovasc Dis 2015;24:751-8.

491. Thiel A, Hartmann A, Rubi-Fessen I et al. Effects of noninvasive brain stimulation on language networks and recovery in early poststroke aphasia. Stroke 2013;44:2240-6.

492. Tosun A, Ture S, Askin A et al. Effects of low-frequency repetitive transcranial magnetic stimulation and neuromuscular electrical stimulation on upper extremity motor recovery in the early period after stroke: a preliminary study. Top Stroke Rehabil 2017;24:361-367.

493. Tretriluxana J, Thanakamchokchai J, Jalayondeja C, Pakaprot N, Tretriluxana S. The Persisted Effects of Low-Frequency Repetitive Transcranial Magnetic Stimulation to Augment Task-Specific Induced Hand Recovery Following Subacute Stroke: Extended Study. Ann Rehabil Med 2018;42:777-787.

494. Triccas LT, Burridge JH, Hughes A, Verheyden G, Desikan M, Rothwell J. A double-blinded randomised controlled trial exploring the effect of anodal transcranial direct current stimulation and uni-lateral robot therapy for the impaired upper limb in sub-acute and chronic stroke. NeuroRehabilitation 2015;37:181-91.

495. Tsai PY, Lin WS, Tsai KT, Kuo CY, Lin PH. High-frequency versus theta burst transcranial magnetic stimulation for the treatment of poststroke cognitive impairment in humans. J Psychiatry Neurosci 2020;45:262-270.

496. Tsai PY, Wang CP, Ko JS, Chung YM, Chang YW, Wang JX. The persistent and broadly modulating effect of inhibitory rTMS in nonfluent aphasic patients: a sham-controlled, double-blind study. Neurorehabil Neural Repair 2014;28:779-87.

497. Uhm KE, Kim YH, Yoon KJ, Hwang JM, Chang WH. BDNF genotype influence the efficacy of rTMS in stroke patients. Neurosci Lett 2015;594:117-21.

498. Ulrichsen KM, Kolskar KK, Richard G et al. No add-on effect of tDCS on fatigue and depression in chronic stroke patients: A randomized sham-controlled trial combining tDCS with computerized cognitive training. Brain Behav 2022;12:e2643.

499. Unluer NO, Temucin CM, Demir N, Serel Arslan S, Karaduman AA. Effects of Low-Frequency Repetitive Transcranial Magnetic Stimulation on Swallowing Function and Quality of Life of Post-stroke Patients. Dysphagia 2019;34:360-371.

500. Valiengo LC, Goulart AC, de Oliveira JF, Bensenor IM, Lotufo PA, Brunoni AR. Transcranial direct current stimulation for the treatment of post-stroke depression: results from a randomised, sham-controlled, double-blinded trial. J Neurol Neurosurg Psychiatry 2017;88:170-175.

501. van Asseldonk EH, Boonstra TA. Transcranial Direct Current Stimulation of the Leg Motor Cortex Enhances Coordinated Motor Output During Walking With a Large Inter-Individual Variability. Brain Stimul 2016;9:182-90.

502. van der Cruijsen J, Piastra MC, Selles RW, Oostendorp TF. A Method to Experimentally Estimate the Conductivity of Chronic Stroke Lesions: A Tool to Individualize Transcranial Electric Stimulation. Front Hum Neurosci 2021;15:738200.

503. van der Vliet R, Ribbers GM, Vandermeeren Y, Frens MA, Selles RW. BDNF Val66Met but not transcranial direct current stimulation affects motor learning after stroke. Brain Stimul 2017;10:882-892.

504. van Lieshout ECC, Visser-Meily JMA, Neggers SFW, van der Worp HB, Dijkhuizen RM. Brain stimulation for arm recovery after stroke (B-STARS): protocol for a randomised controlled trial in subacute stroke patients. BMJ Open 2017;7:e016566.

505. Vasant DH, Michou E, Mistry S, Rothwell JC, Hamdy S. High-frequency focal repetitive cerebellar stimulation induces prolonged increases in human pharyngeal motor cortex excitability. J Physiol 2015;593:4963-77.

506. Vatanparasti S, Kazemnejad A, Yoonessi A, Oveisgharan S. The Effect of Continuous Theta-Burst Transcranial Magnetic Stimulation Combined with Prism Adaptation on the Neglect Recovery in Stroke Patients. J Stroke Cerebrovasc Dis 2019;28:104296.

507. Veldema J, Bosl K, Neumann G, Verheyden G, Nowak DA. Noninvasive brain stimulation in rehabilitation of hemispatial neglect after stroke. CNS Spectr 2020;25:38-49.

508. Viana RT, Laurentino GE, Souza RJ et al. Effects of the addition of transcranial direct current stimulation to virtual reality therapy after stroke: a pilot randomized controlled trial. NeuroRehabilitation 2014;34:437-46.

509. Vila-Nova C, Lucena PH, Lucena R, Armani-Franceschi G, Campbell FQ. Effect of Anodal tDCS on Articulatory Accuracy, Word Production, and Syllable Repetition in Subjects with Aphasia: A Crossover, Double-Blinded, Sham-Controlled Trial. Neurol Ther 2019;8:411-424.

510. Vongvaivanichakul P, Tretriluxana J, Bovonsunthonchai S, Pakaprot N, Laksanakorn W. Reach-to-grasp training in individuals with chronic stroke augmented by low-frequency repetitive transcranial magnetic stimulation. J Med Assoc Thai 2014;97 Suppl 7:S45-9.

511. Waldowski K, Seniow J, Lesniak M, Iwanski S, Czlonkowska A. Effect of low-frequency repetitive transcranial magnetic stimulation on naming abilities in early-stroke aphasic patients: a prospective, randomized, double-blind sham-controlled study. ScientificWorldJournal 2012;2012:518568.

512. Wang C, Chen Y, Song P et al. Varied Response of EEG Rhythm to Different tDCS Protocols and Lesion Hemispheres in Stroke Subjects with Upper Limb Dysfunction. Neural Plast 2022;2022:7790730.

513. Wang CC, Wang CP, Tsai PY, Hsieh CY, Chan RC, Yeh SC. Inhibitory repetitive transcranial magnetic stimulation of the contralesional premotor and primary motor cortices facilitate poststroke motor recovery. Restor Neurol Neurosci 2014;32:825-35.

514. Wang CP, Hsieh CY, Tsai PY, Wang CT, Lin FG, Chan RC. Efficacy of synchronous verbal training during repetitive transcranial magnetic stimulation in patients with chronic aphasia. Stroke 2014;45:3656-62.

515. Wang CP, Tsai PY, Yang TF, Yang KY, Wang CC. Differential effect of conditioning sequences in coupling inhibitory/facilitatory repetitive transcranial magnetic stimulation for poststroke motor recovery. CNS Neurosci Ther 2014;20:355-63.

516. Wang H, Zhang W, Zhao W et al. The efficacy of transcranial alternating current stimulation for treating post-stroke depression: Study Protocol Clinical Trial (SPIRIT Compliant). Medicine (Baltimore) 2020;99:e19671.

517. Wang J, Wu D, Cheng Y et al. Effects of Transcranial Direct Current Stimulation on Apraxia of Speech and Cortical Activation in Patients With Stroke: A Randomized Sham-Controlled Study. Am J Speech Lang Pathol 2019;28:1625-1637.

518. Wang L, Zhu QX, Zhong MH et al. Effects of corticospinal tract integrity on upper limb motor function recovery in stroke patients treated with repetitive transcranial magnetic stimulation. J Integr Neurosci 2022;21:50.

519. Wang Q, Zhang D, Zhao YY, Hai H, Ma YW. Effects of high-frequency repetitive transcranial magnetic stimulation over the contralesional motor cortex on motor recovery in severe hemiplegic stroke: A randomized clinical trial. Brain Stimul 2020;13:979-986.

520. Wang QM, Cui H, Han SJ et al. Combination of transcranial direct current stimulation and methylphenidate in subacute stroke. Neurosci Lett 2014;569:6-11.

521. Wang RY, Tseng HY, Liao KK, Wang CJ, Lai KL, Yang YR. rTMS combined with task-oriented training to improve symmetry of interhemispheric corticomotor excitability and gait performance after stroke: a randomized trial. Neurorehabil Neural Repair 2012;26:222-30.

522. Wang RY, Wang FY, Huang SF, Yang YR. High-frequency repetitive transcranial magnetic stimulation enhanced treadmill training effects on gait performance in individuals with chronic stroke: A double-blinded randomized controlled pilot trial. Gait Posture 2019;68:382-387.

523. Wang Y, Li F, He MJ, Chen SJ. The effects and mechanisms of transcranial ultrasound stimulation combined with cognitive rehabilitation on post-stroke cognitive impairment. Neurol Sci 2022;43:4315-4321.

524. Wang Z, Li J, Wang X, Liu S, Wu W. Effect of transcranial direct-current stimulation on executive function and resting EEG after stroke: A pilot randomized controlled study. J Clin Neurosci 2022;103:141-147.

525. Wang ZY, Chen JM, Lin ZK, Ni GX. Transcranial direct current stimulation improves the swallowing function in patients with cricopharyngeal muscle dysfunction following a brainstem stroke. Neurol Sci 2020;41:569-574.

526. Watanabe K, Kudo Y, Sugawara E et al. Comparative study of ipsilesional and contralesional repetitive transcranial magnetic stimulations for acute infarction. J Neurol Sci 2018;384:10-14.

527. Wei X, Xia N, Li YA et al. Immediate and short-term effects of continuous theta burst transcranial magnetic stimulation over contralesional premotor area on post-stroke spasticity in patients with severe hemiplegia: Study protocol for a randomized controlled trial. Front Neurol 2022;13:895580.

528. Wei YY, Koh CL, Hsu MJ, Lo SK, Chen CH, Lin JH. Effects of Transcranial Direct Current Stimulation Combined With Neuromuscular Electrical Stimulation on Upper Extremity Motor Function in Patients With Stroke. Am J Phys Med Rehabil 2022;101:145-151.

529. Welsby E, Ridding M, Hillier S, Hordacre B. Connectivity as a Predictor of Responsiveness to Transcranial Direct Current Stimulation in People with Stroke: Protocol for a Double-Blind Randomized Controlled Trial. JMIR Res Protoc 2018;7:e10848.

530. Wilkinson G, Sasegbon A, Smith CJ, Rothwell J, Bath PM, Hamdy S. An Exploration of the Application of Noninvasive Cerebellar Stimulation in the Neuro-rehabilitation of Dysphagia after Stroke (EXCITES) Protocol. J Stroke Cerebrovasc Dis 2020;29:104586.

531. Wong MN, Baig FN, Chan YK, Ng ML, Zhu FF, Kwan JSK. Transcranial direct current stimulation over the primary motor cortex improves speech production in post-stroke dysarthric speakers: A randomized pilot study. PLoS One 2022;17:e0275779.

532. Wong PL, Yang YR, Tang SC, Huang SF, Wang RY. Comparing different montages of transcranial direct current stimulation on dual-task walking and cortical activity in chronic stroke: double-blinded randomized controlled trial. BMC Neurol 2022;22:119.

533. Woodhead ZVJ, Kerry SJ, Aguilar OM et al. Randomized trial of iReadMore word reading training and brain stimulation in central alexia. Brain 2018;141:2127-2141.

534. Wu D, Qian L, Zorowitz RD, Zhang L, Qu Y, Yuan Y. Effects on decreasing upper-limb poststroke muscle tone using transcranial direct current stimulation: a randomized sham-controlled study. Arch Phys Med Rehabil 2013;94:1-8.

535. Wu JF, Wang HJ, Wu Y et al. Efficacy of transcranial alternating current stimulation over bilateral mastoids (tACS(bm)) on enhancing recovery of subacute post-stroke patients. Top Stroke Rehabil 2016;23:420-429.

536. Wu Q, Peng T, Liu L et al. The Effect of Constraint-Induced Movement Therapy Combined With Repetitive Transcranial Magnetic Stimulation on Hand Function in Preschool Children With Unilateral Cerebral Palsy: A Randomized Controlled Preliminary Study. Front Behav Neurosci 2022;16:876567.

537. Xu AH, Sun YX. Research hotspots and effectiveness of repetitive transcranial magnetic stimulation in stroke rehabilitation. Neural Regen Res 2020;15:2089-2097.

538. Xu J, Wu Z, Nurnberger A, Sabel BA. Reorganization of Brain Functional Connectivity Network and Vision Restoration Following Combined tACS-tDCS Treatment After Occipital Stroke. Front Neurol 2021;12:729703.

539. Xu M, Zi Y, Wu J et al. Effect of opposing needling on motor cortex excitability in healthy participants and in patients with post-stroke hemiplegia: study protocol for a single-blind, randomised controlled trial. Trials 2021;22:481.

540. Xu R, Zhu GY, Zhu J et al. Using Hebbian-Type Stimulation to Rescue Arm Function After Stroke: Study Protocol for a Randomized Clinical Trial. Front Neural Circuits 2021;15:789095.

541. Yague S, Veciana M, Martinez-Yelamos A et al. Effects of Bihemispheric Transcranial Direct Current Stimulation Combined With Repetitive Peripheral Nerve Stimulation in Acute Stroke Patients. J Clin Neurophysiol 2023;40:63-70.

542. Yamada N, Kakuda W, Kondo T, Mitani S, Shimizu M, Abo M. Local muscle injection of botulinum toxin type a synergistically improves the beneficial effects of repetitive transcranial magnetic stimulation and intensive occupational therapy in post-stroke patients with spastic upper limb hemiparesis. Eur Neurol 2014;72:290-8.

543. Yamada N, Kashiwabara K, Takekawa T et al. Comparison of the effect and treatment sequence between a 2-week parallel repetitive transcranial magnetic stimulation and rehabilitation and a 2-week rehabilitation-only intervention during a 4-week hospitalization for upper limb paralysis after stroke: An open-label, crossover observational study. J Cent Nerv Syst Dis 2022;14:11795735211072731.

544. Yang EJ, Baek SR, Shin J et al. Effects of transcranial direct current stimulation (tDCS) on post-stroke dysphagia. Restor Neurol Neurosci 2012;30:303-11.

545. Yang K, Xi X, Wang T et al. Effects of transcranial direct current stimulation on brain network connectivity and complexity in motor imagery. Neurosci Lett 2021;757:135968.

546. Yang NY, Fong KN, Li-Tsang CW, Zhou D. Effects of repetitive transcranial magnetic stimulation combined with sensory cueing on unilateral neglect in subacute patients with right hemispheric stroke: a randomized controlled study. Clin Rehabil 2017;31:1154-1163.

547. Yang W, Liu TT, Song XB et al. Comparison of different stimulation parameters of repetitive transcranial magnetic stimulation for unilateral spatial neglect in stroke patients. J Neurol Sci 2015;359:219-25.

548. Yang Y, Pan H, Pan W et al. Repetitive Transcranial Magnetic Stimulation on the Affected Hemisphere Enhances Hand Functional Recovery in Subacute Adult Stroke Patients: A Randomized Trial. Front Aging Neurosci 2021;13:636184.

549. Yang Z, Qiao L, He J, Zhao X, Zhang M. Effects of repetitive transcranial magnetic stimulation combined with functional electrical stimulation on hand function of stroke: A randomized controlled trial. NeuroRehabilitation 2022;51:283-289.

550. Yao X, Cui L, Wang J, Feng W, Bao Y, Xie Q. Effects of transcranial direct current stimulation with virtual reality on upper limb function in patients with ischemic stroke: a randomized controlled trial. J Neuroeng Rehabil 2020;17:73.

551. Yi YG, Chun MH, Do KH, Sung EJ, Kwon YG, Kim DY. The Effect of Transcranial Direct Current Stimulation on Neglect Syndrome in Stroke Patients. Ann Rehabil Med 2016;40:223-9.

552. Yin M, Liu Y, Zhang L et al. Effects of rTMS Treatment on Cognitive Impairment and Resting-State Brain Activity in Stroke Patients: A Randomized Clinical Trial. Front Neural Circuits 2020;14:563777.

553. Yu C, Wang W, Zhang Y et al. The Effects of Modified Constraint-Induced Movement Therapy in Acute Subcortical Cerebral Infarction. Front Hum Neurosci 2017;11:265.

554. Yu H, Liu S, Dai P, Wang Z, Liu C, Zhang H. Effects of Repetitive Transcranial Magnetic Stimulation on Gait and Postural Control Ability of Patients with Executive Dysfunction after Stroke. Brain Sci 2022;12.

555. Yu P, Wang Y, Yuan J et al. Observation for the effect of rTMS combined with magnetic stimulation at Neiguan (PC6) and Sanyinjiao (SP6) points on limb function after stroke: A study protocol. Medicine (Baltimore) 2020;99:e22207.

556. Yu-Lei X, Shan W, Ju Y, Yu-Han X, Wu Q, Yin-Xu W. Theta burst stimulation versus high-frequency repetitive transcranial magnetic stimulation for poststroke dysphagia: A randomized, double-blind, controlled trial. Medicine (Baltimore) 2022;101:e28576.

557. Yuan X, Yang Y, Cao N, Jiang C. Promotion of Poststroke Motor-Function Recovery with Repetitive Transcranial Magnetic Stimulation by Regulating the Interhemispheric Imbalance. Brain Sci 2020;10.

558. Yun GJ, Chun MH, Kim BR. The Effects of Transcranial Direct-Current Stimulation on Cognition in Stroke Patients. J Stroke 2015;17:354-8.

559. Zandvliet SB, Meskers CG, Nijland RH, Daffertshofer A, Kwakkel G, van Wegen EE. The effect of cerebellar transcranial direct current stimulation to improve standing balance performance early post-stroke, study protocol of a randomized controlled trial. Int J Stroke 2019;14:650-657.

560. Zandvliet SB, Meskers CGM, Kwakkel G, van Wegen EEH. Short-Term Effects of Cerebellar tDCS on Standing Balance Performance in Patients with Chronic Stroke and Healthy Age-Matched Elderly. Cerebellum 2018;17:575-589.

561. Zhang C, Zheng X, Lu R, Yun W, Yun H, Zhou X. Repetitive transcranial magnetic stimulation in combination with neuromuscular electrical stimulation for treatment of post-stroke dysphagia. J Int Med Res 2019;47:662-672.

562. Zhang D, Wang Y, Li H et al. The central-peripheral coupling effect of ocular acupuncture kinesitherapy in post-stroke dyskinesia: A functional neuroimaging and neurotic electrophysiology study protocol. Front Neurol 2022;13:977112.

563. Zhang JJ, Fong KNK. Effects of priming intermittent theta burst stimulation on upper limb motor recovery after stroke: study protocol for a proof-of-concept randomised controlled trial. BMJ Open 2020;10:e035348.

564. Zhang M, Wei J, Wu X. Effects of whole-body vibration training on lower limb motor function and neural plasticity in patients with stroke: protocol for a randomised controlled clinical trial. BMJ Open 2022;12:e060796.

565. Zhang RG, Liu SX, Wang FY, Ma XC, Yang YH. [Treatment of Unilateral Neglect using Repetitive Transcranial Magnetic Stimulation (rTMS) and Sensory Cueing (SC) in Stroke Patients]. Sichuan Da Xue Xue Bao Yi Xue Ban 2017;48:309-313.

566. Zhang XH, Gu T, Liu XW et al. The Effect of Transcranial Direct Current Stimulation and Functional Electrical Stimulation on the Lower Limb Function of Stroke Patients. Front Neurosci 2021;15:685931.

567. Zhao CG, Sun W, Ju F et al. Analgesic Effects of Navigated Repetitive Transcranial Magnetic Stimulation in Patients With Acute Central Poststroke Pain. Pain Ther 2021;10:1085-1100.

568. Zhao J, Li Y, Zhang X et al. Alteration of network connectivity in stroke patients with apraxia of speech after tDCS: A randomized controlled study. Front Neurol 2022;13:969786.

569. Zhao L, Liu Z, Sun Q, Li H. Effect of transcranial direct current stimulation combined with a smart hand joint training device on hand dysfunction in patients with early stroke. Folia Neuropathol 2022;60:177-184.

570. Zhao N, Zhang J, Qiu M et al. Scalp acupuncture plus low-frequency rTMS promotes repair of brain white matter tracts in stroke patients: A DTI study. J Integr Neurosci 2018;17:61-69.

571. Zhao Q, Wang J, Li Z, Song L, Li X. Effect of Anodic Transcranial Direct Current Stimulation Combined With Speech Language Therapy on Nonfluent Poststroke Aphasia. Neuromodulation 2021;24:923-929.

572. Zhao S, Dou Z, Wei X et al. Task-concurrent anodal tDCS modulates bilateral plasticity in the human suprahyoid motor cortex. Front Hum Neurosci 2015;9:370.

573. Zheng CJ, Liao WJ, Xia WG. Effect of combined low-frequency repetitive transcranial magnetic stimulation and virtual reality training on upper limb function in subacute stroke: a double-blind randomized controlled trail. J Huazhong Univ Sci Technolog Med Sci 2015;35:248-254.

574. Zheng K, Chen M, Shen Y et al. Cerebellar Continuous Theta Burst Stimulation for Aphasia Rehabilitation: Study Protocol for a Randomized Controlled Trial. Front Aging Neurosci 2022;14:909733.

575. Zheng MX, Hua XY, Feng JT et al. Trial of Contralateral Seventh Cervical Nerve Transfer for Spastic Arm Paralysis. N Engl J Med 2018;378:22-34.

576. Zhong L, Rao J, Wang J et al. Repetitive Transcranial Magnetic Stimulation at Different Sites for Dysphagia After Stroke: A Randomized, Observer-Blind Clinical Trial. Front Neurol 2021;12:625683.

577. Zhou J, Fan L, Hu H et al. The Efficacy of Integrated Rehabilitation for Post-Stroke Anxiety: Study Protocol for a Prospective, Multicenter, Randomized Controlled Trial. Int J Gen Med 2022;15:7101-7111.

578. Zimerman M, Heise KF, Hoppe J, Cohen LG, Gerloff C, Hummel FC. Modulation of training by single-session transcranial direct current stimulation to the intact motor cortex enhances motor skill acquisition of the paretic hand. Stroke 2012;43:2185-91.

579. Zult T, Goodall S, Thomas K, Solnik S, Hortobagyi T, Howatson G. Mirror Training Augments the Cross-education of Strength and Affects Inhibitory Paths. Med Sci Sports Exerc 2016;48:1001-13.

580. Zumbansen A, Black SE, Chen JL et al. Non-invasive brain stimulation as add-on therapy for subacute post-stroke aphasia: a randomized trial (NORTHSTAR). Eur Stroke J 2020;5:402-413.

581. Zumbansen A, Kneifel H, Lazzouni L et al. Differential Effects of Speech and Language Therapy and rTMS in Chronic Versus Subacute Post-stroke Aphasia: Results of the NORTHSTAR-CA Trial. Neurorehabil Neural Repair 2022;36:306-316.
